# Effects of intensive insulin therapy combined with non-insulin antihyperglycemic agents on glycemic control, variability, and metabolic outcomes in patients with type 2 diabetes mellitus: a systematic review and meta-analysis

**DOI:** 10.3389/fendo.2026.1948361

**Published:** 2026-10-05

**Authors:** Zixuan Shen, Jiawei Li, Ziyun Zhao, Ziang Chen, Xiaosen Ma, Jing Zhou, Lijing Jiao, Jing Wang

**Affiliations:** 1 The Second Hospital of Hebei Medical University, Shijiazhuang, China; 2 Department of Endocrinology, The Second Hospital of Hebei Medical University, Shijiazhuang, China

**Keywords:** glycemic variability, intensive insulin therapy, meta-analysis, type 2 diabetes, weight

## Abstract

**Background:**

Intensive insulin therapy (IIT) induces clinical remission in newly diagnosed or poorly controlled type 2 diabetes mellitus (T2DM), but is limited by hypoglycemia risk and weight gain. Combining IIT with non-insulin antihyperglycemic agents may mitigate these drawbacks while improving glycemic variability.

**Methods:**

This review was registered with PROSPERO (CRD42024582974). We searched PubMed, Embase, Cochrane, Web of Science, ClinicalTrials.gov, and Chinese databases (CNKI, VIP, Wanfang, and Chinese Medical Association journals) for randomized controlled trials (RCTs) comparing intensive insulin therapy (IIT) alone with IIT plus metformin, dipeptidyl peptidase-4 inhibitors (DPP-4i), sodium-glucose cotransporter-2 inhibitors (SGLT2i), or glucagon-like peptide-1 receptor agonists (GLP-1RA) in adults with type 2 diabetes mellitus (T2DM). The primary outcomes were glycemic variability indices, including mean amplitude of glycemic excursions (MAGE), standard deviation of blood glucose (SDBG), and largest amplitude of glycemic excursions (LAGE). Secondary outcomes included fasting plasma glucose (FPG), 2-h postprandial glucose (PPG-2h), mean blood glucose (MBG), glycated hemoglobin (HbA1c), homeostasis model assessment of β-cell function (HOMA-β), homeostasis model assessment of insulin resistance (HOMA-IR), and insulin requirements, including total daily insulin dose (TDD), basal insulin dose, and bolus insulin dose. Time in range (TIR), body weight, hypoglycemia, and gastrointestinal adverse events (GI-AEs) were analyzed as *post hoc* exploratory outcomes.

**Results:**

Thirty-nine RCTs were included. For the primary glycemic-variability outcomes, GRADE assessments indicated that IIT plus DPP-4i likely reduced MAGE (MD −1.39 mmol/L, 95% CI −1.77 to −1.01) and LAGE (MD −1.20 mmol/L, 95% CI −1.66 to −0.75), while IIT plus SGLT2i likely reduced SDBG (MD −0.28 mmol/L, 95% CI −0.44 to −0.11). Evidence was very uncertain for the effects of IIT plus GLP-1RA on MAGE and IIT plus DPP-4i on SDBG. Among secondary outcomes, IIT plus DPP-4i likely reduced FPG (MD −0.44 mmol/L, 95% CI −0.67 to −0.21), while IIT plus GLP-1RA likely reduced HbA1c (MD −0.73%, 95% CI −1.01 to −0.45). IIT plus GLP-1RA, metformin, or SGLT2i may reduce FPG; IIT plus DPP-4i, GLP-1RA, or metformin may reduce PPG-2h; and IIT plus SGLT2i may reduce HbA1c. IIT plus DPP-4i and IIT plus GLP-1RA may also modestly reduce total daily insulin dose. Evidence for the effect of IIT plus SGLT2i on PPG-2h was very uncertain. Among *post hoc* exploratory outcomes, IIT plus SGLT2i likely increased TIR (MD 8.23 percentage points, 95% CI 4.13 to 12.32), while IIT plus DPP-4i may reduce hypoglycemia risk (RR 0.53, 95% CI 0.31 to 0.91) and IIT plus GLP-1RA may increase GI-AEs (RR 5.28, 95% CI 1.81 to 15.40). Longer-duration IIT plus GLP-1RA may be associated with greater weight loss (MD −3.63 kg, 95% CI −4.94 to −2.32) in a *post hoc* exploratory subgroup analysis, whereas evidence for the effect of IIT plus SGLT2i on hypoglycemia was very uncertain.

**Conclusions:**

Adjunctive non-insulin antihyperglycemic agents may improve glycemic variability and overall glycemic control in adults with T2DM receiving intensive insulin therapy. The most consistent evidence supported reductions in MAGE and LAGE with DPP-4i and in SDBG with SGLT2i. Reductions in FPG and PPG-2h were observed across several drug classes, while HbA1c was reduced with GLP-1RA and SGLT2i. *Post hoc* analyses suggested improved TIR with SGLT2i, reduced body weight with longer-duration GLP-1RA therapy, a lower risk of hypoglycemia with DPP-4i, and a higher risk of gastrointestinal adverse events with GLP-1RA; however, these exploratory findings should be interpreted cautiously.

**Systematic Review registration:**

https://www.crd.york.ac.uk/PROSPERO/, identifier CRD42024582974.

## 1 Introduction

Intensive insulin therapy (IIT) regimens include basal-bolus insulin, and continuous subcutaneous insulin infusion (CSII). It was used for patients with newly diagnosed or poorly controlled type 2 diabetes mellitus (T2DM). And short-term IIT can rapidly alleviate glucose toxicity, improve β-cell function, and induce clinical remission in some individuals (1). Despite its potent efficacy in controlling hyperglycemia, insulin therapy carries risks of hypoglycemia and weight gain that may heighten cardiovascular event risk. These risks can be attenuated through combination therapy with oral antidiabetic agents (2). Common non-insulin antihyperglycemic agents include Metformin (Met), dipeptidyl peptidase-4 inhibitors (DPP-4i), sodium-glucose co-transporter 2 inhibitors (SGLT2i), and glucagon-like peptide-1 receptor agonists (GLP-1RA). Met, a classic first-line medication, improves hepatic and peripheral insulin sensitivity. DPP-4i enhances insulin secretion and suppress glucagon in a glucose-dependent manner, featuring low hypoglycemia risk. SGLT2i lowers blood glucose by promoting urinary glucose excretion via an insulin-independent mechanism, offering additional benefits of weight reduction and blood pressure lowering that may theoretically decrease insulin dosage (3). GLP-1RA potently lower glucose while significantly reducing body weight through central appetite suppression, delayed gastric emptying, and other mechanisms, acting in a glucose-dependent manner with minimal hypoglycemia risk (3).

Long-term, stable glycemic control is widely recognized as the cornerstone for preventing diabetic microvascular and macrovascular complications. In clinical practice, glycated hemoglobin (HbA1c) has long served as the “gold standard” for assessing long-term glycemic status. However, as an indicator reflecting average glucose levels over the preceding 2–3 months, HbA1c possesses inherent limitations: it cannot capture glycemic variability. With the widespread adoption of continuous glucose monitoring (CGM) technology, novel metrics such as time in range (TIR), standard deviation of blood glucose(SDBG), mean amplitude of glycemic excursions(MAGE) have garnered increasing attention. TIR refers to the duration or percentage of time within a 24-hour period that glucose levels remain within the target range (typically 3.9–10.0 mmol/L), offering a more intuitive and comprehensive assessment of glycemic control quality (4).

Current evidence suggests that CSII combined with some of the aforementioned agents is superior to IIT alone in reducing HbA1c (5). Previous evidence, including a network meta-analysis by Li et al. (5), has evaluated the efficacy of continuous subcutaneous insulin infusion (CSII) combined with oral glucose-lowering agents, primarily focusing on HbA1c reduction. However, these studies did not specifically address glycemic variability (GV) metrics derived from continuous glucose monitoring, such as TIR, MAGE, and SDBG.

In contrast, the present study places particular emphasis on glycemic variability while also evaluating conventional glycemic, metabolic, insulin-dose, and safety outcomes. and includes both CSII and multiple daily injection (MDI) regimens, thereby providing a more comprehensive and clinically generalizable assessment of combination strategies.

This study aimed to comprehensively evaluate, through a systematic review and meta-analysis, the effects of IIT combined with DPP-4i, metformin, SGLT2i, or GLP-1RA, compared with IIT alone, in adults with T2DM. The primary outcome domain comprised glycemic variability indices, specifically mean amplitude of glycemic excursions (MAGE), standard deviation of blood glucose (SDBG), and largest amplitude of glycemic excursions (LAGE). Secondary outcomes included fasting plasma glucose (FPG), 2-h postprandial glucose (PPG-2h), mean blood glucose (MBG), glycated hemoglobin (HbA1c), homeostasis model assessment of β-cell function (HOMA-β), homeostasis model assessment of insulin resistance (HOMA-IR), and insulin requirements, including total daily insulin dose (TDD), basal insulin dose, and bolus insulin dose. Time in range (TIR), body weight, hypoglycemia, and gastrointestinal adverse events (GI-AEs) were evaluated as *post hoc* exploratory outcomes.

## 2 Materials and methods

### 2.1 Protocol registration and amendments

This review was prospectively registered in PROSPERO (CRD42024582974) and was conducted in accordance with the Preferred Reporting Items for Systematic Reviews and Meta-Analyses (PRISMA) guidelines. The original registration prespecified the efficacy outcomes but did not define a formal primary-versus-secondary outcome hierarchy. To ensure consistent reporting across the Abstract, Introduction, Methods, and Results, a hierarchical outcome framework was subsequently adopted. Glycemic variability indices, including mean amplitude of glycemic excursions (MAGE), standard deviation of blood glucose (SDBG), and largest amplitude of glycemic excursions (LAGE), were designated as the primary outcome domain, whereas the other registered metabolic and insulin-dose outcomes were classified as secondary outcomes for reporting purposes. Time in range (TIR), body weight, hypoglycemia, and gastrointestinal adverse events (GI-AEs) were analyzed as *post hoc* exploratory outcomes.The originally registered search end date of 10 August 2024 was subsequently extended to 7 November 2025. Chinese bibliographic databases were added to broaden the coverage of the literature search. The eligibility criteria were clarified to exclude trials in which the randomized insulin regimen itself consisted exclusively of premixed insulin, while allowing previous use of premixed insulin when participants were switched to an eligible intensive insulin therapy (IIT) regimen during the randomized intervention period. Risk of bias was reassessed using the Cochrane Risk of Bias 2 (RoB 2) tool, the certainty of evidence was evaluated using the Grading of Recommendations Assessment, Development and Evaluation (GRADE) approach, and random-effects models were used for the primary meta-analyses. These post-registration amendments are reported transparently to distinguish them from the procedures specified in the original PROSPERO registration.

### 2.2 Inclusion and exclusion criteria

Inclusion criteria: (1) Adults (≥18 years) with T2DM meeting one of the following treatment-context categories: P1, previously untreated/treatment-naïve; P2, previously diagnosed but inadequately controlled T2DM initiating corrective IIT; or P3, previously diagnosed T2DM receiving maintenance IIT with CSII or basal–bolus MDI, in whom a non-insulin antihyperglycemic agent was added; (2) randomized controlled trials (RCTs); (3) studies comparing IIT alone or continued background IIT with the same IIT regimen combined with a non-insulin antihyperglycemic agent; (4) Trials were eligible when the randomized treatment period used IIT, defined as CSII or MDI. Trials in which the randomized insulin regimen itself consisted exclusively of premixed insulin were excluded. Previous use of premixed insulin before randomization was not an exclusion criterion if participants were switched to an eligible CSII or MDI regimen during the randomized intervention period. In multi-arm studies containing both eligible and ineligible intervention arms, only the eligible pairwise comparison was extracted; if an eligible contrast could not be isolated, the study was excluded from quantitative synthesis.; and (5) Eligible studies were required to report at least one of the following outcomes. The primary outcome domain comprised glycemic variability indices assessed by CGM or SMBG, specifically MAGE, SDBG, and LAGE. Secondary outcomes included fasting FPG, PPG-2h, MBG, HbA1c, HOMA-β, HOMA-IR, and insulin requirements, including TDD, basal insulin dose, and bolus insulin dose. These outcomes were eligible whether reported at the treatment endpoint or as changes from baseline. TIR, body weight, hypoglycemia, and GI-AEs were analyzed as *post hoc* exploratory outcomes. Exclusion criteria: (1) Pregnancy with T2DM; (2) Absence of the aforementioned outcomes; (3) Duplicate publications; (4) Unavailability of the full text.

### 2.3 Literature sources and search strategy

We searched ClinicalTrials.gov, PubMed, Embase, the Cochrane Library, Web of Science, CNKI, VIP, Wanfang, and Chinese Medical Association journals from inception to November 7, 2025. Controlled vocabulary and free-text terms were combined, and database-specific strategies were used to identify RCTs. Detailed search strategies for each database are provided (**Supplementary Material 5**).

### 2.4 Study selection, data extraction, and quality assessment

#### 2.4.1 Study selection

Five investigators independently screened titles and abstracts using Medical.

Reference King 6.0 software. Disagreements were resolved by a third reviewer. We extracted the following information using a pre-designed data collection form: study characteristics (author, publication year and study design); interventions (number of participants in each group, control group, and intervention details); outcome measures (FPG, PPG-2h, HbA1c, HOMA-β,HOMA-IR,or glycemic variability indices obtained via CGM or SMBG such as MBG, MAGE, SDBG and TIR,or body weight, TDD, basal insulin dose, bolus insulin dose); and study bias indicators and quality assessment.

#### 2.4.2 Data extraction

Trial data were extracted using a standardized data extraction form and independently reviewed by two reviewers (Shen and Zhao). We extracted information on study design and population characteristics, allocated sample size, age, diabetes duration and relevant treatment history, HbA1c eligibility criteria and baseline HbA1c, BMI, intervention and comparator regimens, and treatment duration. In addition to the outcomes prespecified in PROSPERO, we also extracted additional metabolic outcomes, including time in range (TIR) and body weight, as well as safety outcomes. HOMA-β was reported in 12 studies but was not pooled because of differences in calculation methods and reporting units.

For safety outcomes, we further extracted trial-level information on hypoglycemia, gastrointestinal adverse events (GI-AEs), diabetic ketoacidosis (DKA), and treatment discontinuation, including hypoglycemia definitions and assessment windows, GI-AE definitions or symptom categories, DKA diagnostic criteria and events, and the frequency and reasons for treatment discontinuation, where available. Quantitative synthesis was performed only when outcome definitions and event metrics were sufficiently comparable across studies; otherwise, safety findings were summarized descriptively.

#### 2.4.3 Quality assessment

Risk of bias was independently assessed by two reviewers using the Cochrane Risk of Bias 2 (RoB 2) tool across five domains: randomization process, deviations from intended interventions, missing outcome data, outcome measurement, and selection of the reported result. Each domain and the overall judgment were rated as low risk, some concerns, or high risk. Disagreements were resolved by discussion or consultation with a third reviewer. Results are shown in **Figure 1**.

**Figure 1:**
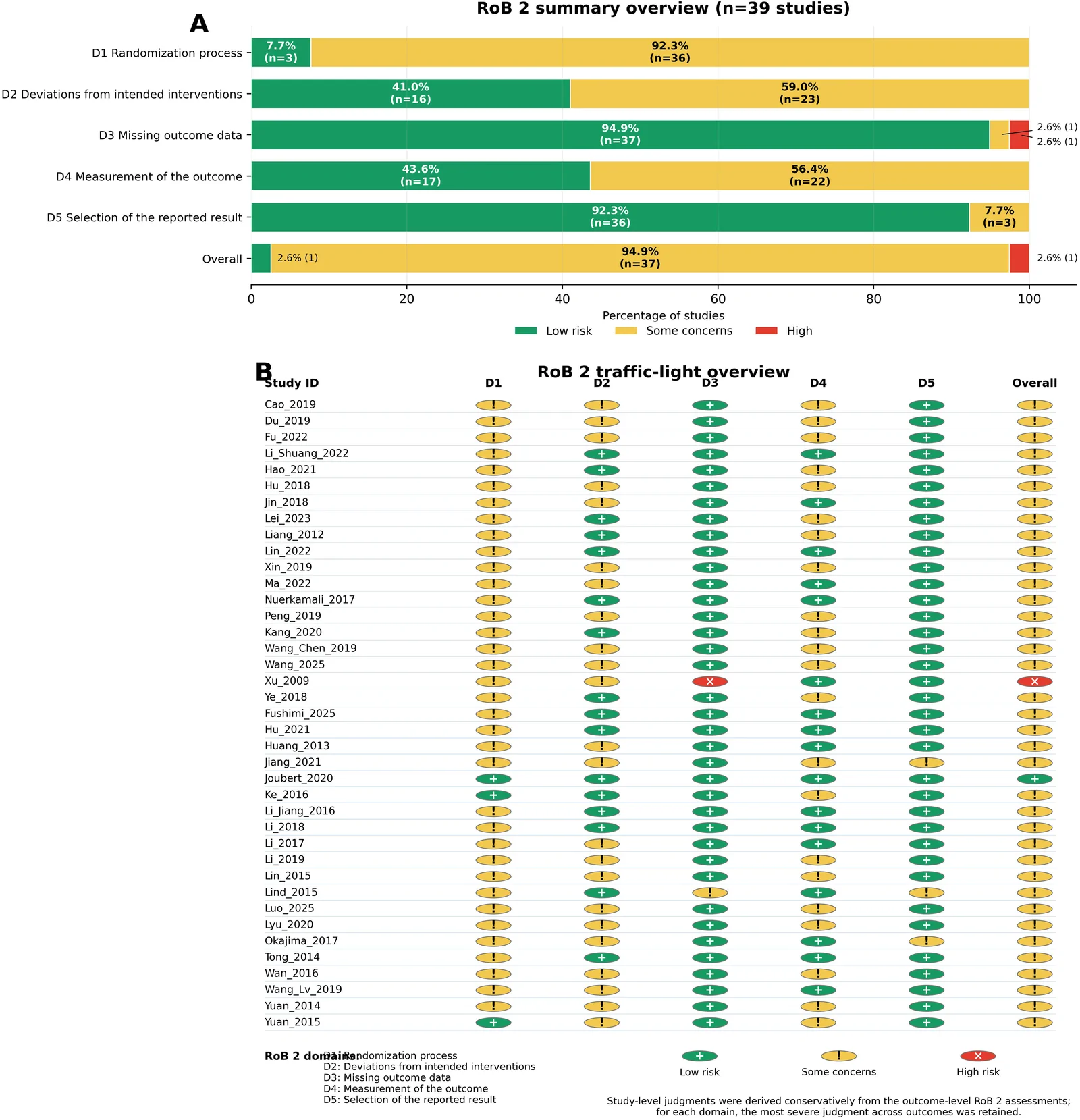
Risk-of-bias assessment of the included studies using the Cochrane Risk of Bias 2 (RoB 2) tool. **(A)** Summary of the proportions of studies rated as low risk, some concerns, or high risk in each domain and overall. **(B)** Traffic-light plot of study-level judgments. D1, randomization process; D2, deviations from intended interventions; D3, missing outcome data; D4, measurement of the outcome; D5, selection of the reported result.

### 2.5 Statistical analysis

Meta-analyses were performed using Stata 18 (StataCorp, College Station, TX, USA). Continuous outcomes were summarized as mean differences (MDs) with 95% confidence intervals (CIs) after harmonizing units where necessary, and dichotomous outcomes as risk ratios (RRs) with 95% CIs. Post-intervention values and change-from-baseline values were pooled using MDs when outcomes were measured on the same scale. Because of the limited number of studies within each data type, subgroup analyses comparing post-intervention values with change-from-baseline values were not performed; instead, sensitivity analyses were conducted to assess whether data type influenced the pooled estimates. When an SD was not reported, it was derived using standard equations from the reported standard error (SE), CI, or P value, or calculated from the reported SDs of the two groups, as appropriate (6, 7).

Random-effects models were used throughout because clinical and methodological heterogeneity was anticipated. Heterogeneity was assessed using I², τ², and Cochran’s Q test. Outcome-specific subgroup analyses were conducted, as clinically appropriate, according to drug class, patient category (P1, P2, or P3), or IIT modality, or treatment duration (Long-term group [>4 weeks], Short-term group [ ≤ 4 weeks]) depending on the most relevant potential source of heterogeneity for each outcome. For glycemic variability outcomes, because the number of studies within individual drug subgroups was limited, additional formal subgroup analyses were generally avoided; instead, sensitivity analyses restricted by IIT modality were conducted where clinically relevant. Leave-one-out sensitivity analyses were also performed to evaluate the influence of individual studies on pooled estimates. Funnel plots and Egger’s test were used to assess small-study effects. All tests were two-sided, with P<0.05 considered statistically significant.

Risk of bias and certainty of evidence. Risk of bias was independently assessed by two reviewers using the Cochrane Risk of Bias 2 (RoB 2) tool across five domains: randomization, deviations from intended interventions, missing outcome data, outcome measurement, and selection of the reported result. Each result was rated as low risk, some concerns, or high risk, with disagreements resolved by discussion or consultation with a third reviewer. Certainty of evidence for key outcomes was assessed using the GRADE approach, considering risk of bias, inconsistency, indirectness, imprecision, and publication bias, and was rated as high, moderate, low, or very low. Summary of Findings tables were prepared for key outcomes.

## 3 Results

### 3.1 Search results

As shown in **Figure 2**, 5,926 records were identified from databases and 76 from trial registries, for a total of 6,002 records. After screening and eligibility assessment, 39 (8–46) independent RCTs were included in the systematic review and quantitative synthesis.

**Figure 2:**
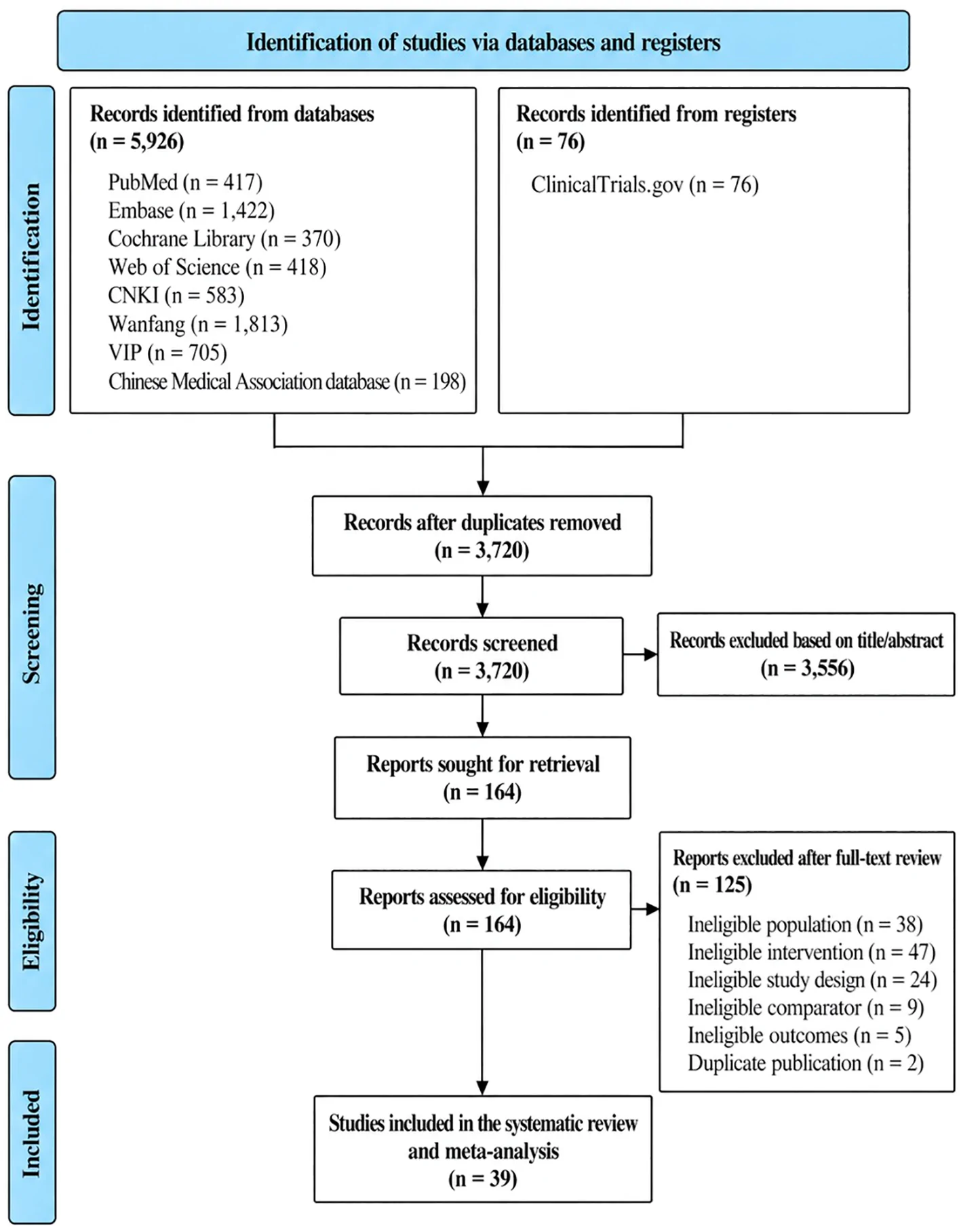
Preferred Reporting Items for Systematic Reviews and Meta-Analyses (PRISMA) 2020 flow diagram of study selection. The diagram shows identification, screening, eligibility assessment, and inclusion of records from international and Chinese databases and a trial registry. Thirty-nine studies were included in the systematic review and meta-analysis.

### 3.2 Included eligible study characteristics

The 39 included studies evaluated DPP-4 inhibitors (7 studies), metformin (12 studies), GLP-1 receptor agonists (11 studies), and SGLT2 inhibitors (9 studies). The randomized intervention duration ranged from 3 days to 6 months.

Reported outcomes included body weight, insulin requirements, measures of glycemic control (MBG, FPG, PPG-2h, and HbA1c), indices of glycemic variability (SDBG, MAGE, and LAGE), time in range (TIR), and measures of β-cell function and insulin resistance (**Supplementary Table 1**).

### 3.3 Risk of bias

Using the Cochrane RoB 2 tool, most of the 39 included studies were judged as having some concerns overall (94.9%, n=37), while one study was rated as low risk (2.6%, n=1) and one as high risk (2.6%, n=1). Most studies had some concerns regarding the randomization process (92.3%, n=36), deviations from intended interventions (59.0%, n=23), and measurement of the outcome (56.4%, n=22). In contrast, missing outcome data and selection of the reported result were predominantly rated as low risk, at 94.9% (n=37) and 92.3% (n=36), respectively (**Figure 1**). Outcome-level RoB 2 assessments are provided in **Supplementary Material 1**.

### 3.4 Effectiveness

#### 3.4.1 Core glycemic variability metrics

##### 3.4.1.1 SDBG

A total of 10 studies showed that IIT combined with non-insulin antihyperglycemic agents significantly reduced SDBG (MD −0.31mmol/L, 95% CI −0.47 to −0.16, P<0.001), with substantial heterogeneity (I²=67.1%) (**Figure 3a**). Subgroup analysis by drug class showed significant reductions in the SGLT2i subgroup (3 studies, MD −0.28mmol/L, 95% CI −0.44 to −0.11, P=0.001); based on very low certainty evidence, DPP-4i might be associated with a reduction in SDBG (2 studies, MD −0.46mmol/L, 95% CI −0.75 to −0.17, P=0.002); and data from a single trial suggested that metformin might be associated with a reduction in SDBG (1 study, MD −0.59mmol/L, 95% CI −0.77 to −0.41, P<0.001), whereas the effect of GLP-1RA was not statistically significant (4 studies, MD −0.13mmol/L, 95% CI −0.46 to 0.20, P=0.43). A significant difference was observed across drug subgroups (P=0.03).

**Figure 3:**
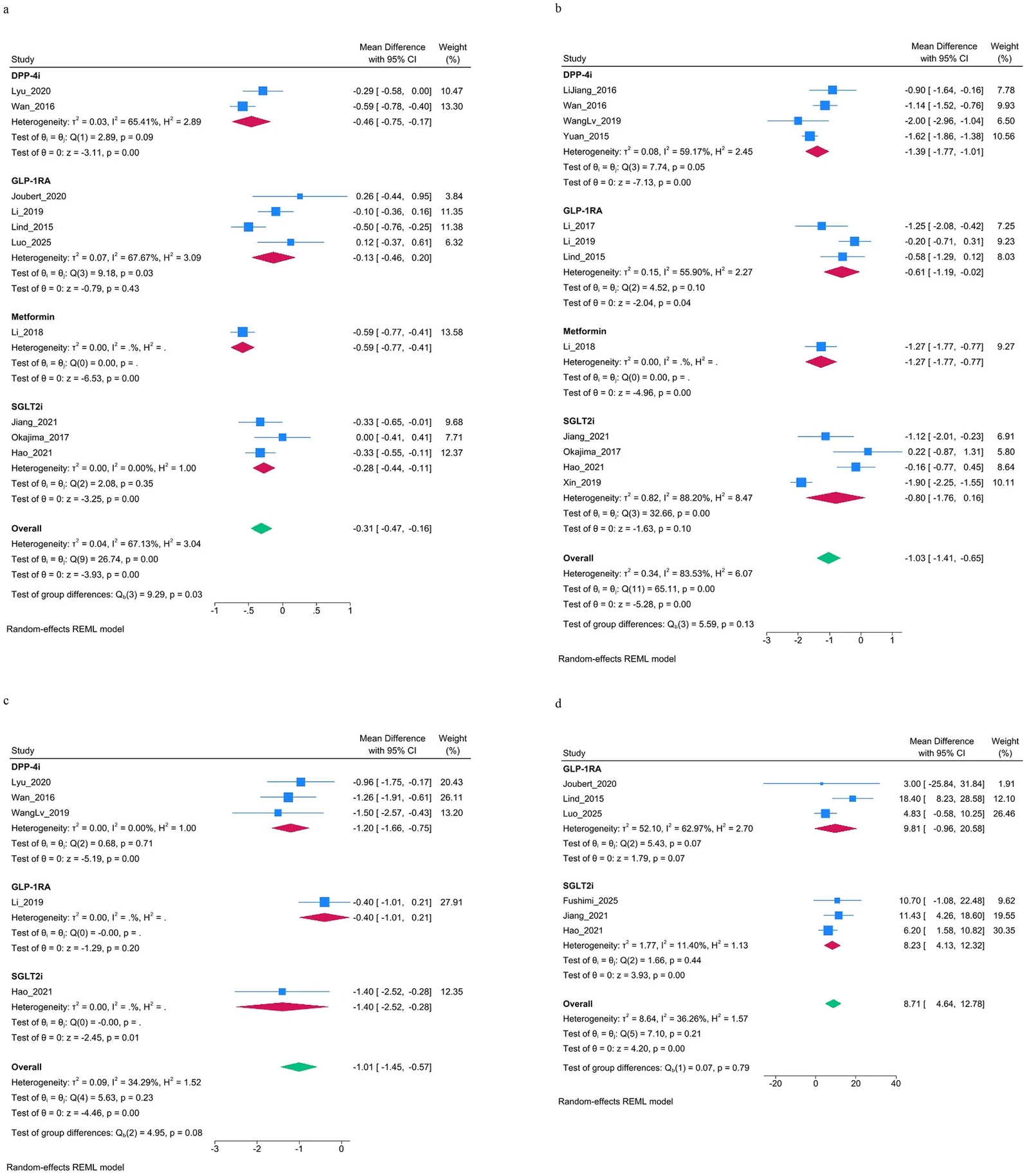
Forest plots of glycemic variability outcomes. **(a)** Standard deviation of blood glucose (SDBG); **(b)** mean amplitude of glycemic excursions (MAGE); **(c)** largest amplitude of glycemic excursions (LAGE); and **(d)** time in range (TIR). Pooled estimates were obtained using random-effects models.

Sensitivity analyses were subsequently performed within drug subgroups where applicable. In the GLP-1RA subgroup, the pooled effect remained non-significant after excluding the study with unclear IIT modality (MD −0.20mmol/L, 95% CI −0.55 to 0.15, P=0.27) and after excluding the MDI study (MD −0.02mmol/L, 95% CI −0.24 to 0.19, P=0.84). In the SGLT2i subgroup, exclusion of the CSII study attenuated the effect and rendered it non-significant (MD −0.21mmol/L, 95% CI −0.52 to 0.10, P=0.18), suggesting that the pooled estimate should be interpreted cautiously.

Sensitivity analysis stratified by data type also identified a statistically significant difference between endpoint-value and change-from-baseline estimates within the GLP-1RA subgroup (endpoint values: 3 studies, MD −0.02 mmol/L, 95% CI −0.24 to 0.20; change from baseline: 1 study, MD −0.50 mmol/L, 95% CI −0.76 to −0.25; P for subgroup difference=0.005). Because the change-from-baseline estimate was derived from a single study, this comparison was considered exploratory and should not be interpreted as definitive evidence of effect modification by data type (**Supplementary Material 4**).

Egger’s test indicated evidence of small-study effects (P<0.001). Trim-and-fill analysis imputed three potentially missing studies; however, the adjusted pooled effect remained statistically significant (MD −0.40mmol/L, 95% CI −0.57 to −0.23), suggesting that the overall direction of the effect was robust to this adjustment(**Supplementary Figures 2a, b**).

##### 3.4.1.2 MAGE

Compared with IIT alone, IIT combined with non-insulin antihyperglycemic agents significantly reduced MAGE (MD −1.03mmol/L, 95% CI −1.41 to −0.65, P<0.001), with substantial heterogeneity (I²=83.5%) (**Figure 3b**). Subgroup analysis by drug class showed significant reductions with DPP-4i (4 studies, MD −1.39mmol/L, 95% CI −1.77 to −1.01, P<0.001); based on very low certainty evidence, GLP-1RA might be associated with a reduction in MAGE (3 studies, MD −0.61mmol/L, 95% CI −1.19 to −0.02, P=0.04); and data from a single trial suggested that metformin might be associated with a reduction in MAGE (1 study, MD −1.27mmol/L, 95% CI −1.77 to −0.77, P<0.001), whereas the effect of SGLT2i was not statistically significant (4 studies, MD −0.80mmol/L, 95% CI −1.76 to 0.16, P=0.10). No significant difference was observed between drug subgroups (P=0.13).

Sensitivity analyses were performed within subgroups where applicable. In the DPP-4i subgroup, exclusion of the study with unclear IIT modality yielded a similar significant effect (MD −1.20mmol/L, 95% CI −1.55 to −0.85, P<0.001). In the GLP-1RA subgroup, exclusion of the MDI study attenuated the pooled effect and rendered it non-significant (MD −0.67mmol/L, 95% CI −1.70 to 0.35, P=0.20). In the SGLT2i subgroup, exclusion of the CSII study also resulted in a non-significant effect (MD −0.68mmol/L, 95% CI −1.99 to 0.64, P=0.31). Egger’s test showed no evidence of small-study effects (P=0.230) (**Supplementary Figure 2c**).

##### 3.4.1.3 LAGE

Compared with IIT alone, IIT combined with non-insulin antihyperglycemic agents significantly reduced LAGE (MD −1.01mmol/L, 95% CI −1.45 to −0.57, P<0.001), with moderate heterogeneity (I²=34.3%) (**Figure 3c**). Subgroup analysis by drug class showed a significant reduction with DPP-4i (3 studies, MD −1.20mmol/L, 95% CI −1.66 to −0.75, P<0.001), whereas the effect of GLP-1RA was not statistically significant (1 study, MD −0.40mmol/L, 95% CI −1.01 to 0.21, P=0.20). Data from a single trial suggested that SGLT2i might be associated with a reduction in LAGE (1 study, MD −1.40mmol/L, 95% CI −2.52 to −0.28, P=0.01).No significant difference was observed between drug subgroups (P=0.08). Egger’s test showed no evidence of small-study effects (P=0.175) (**Supplementary Figure 2d**).

#### 3.4.2 TIR

Overall analysis showed that combination therapy significantly increased TIR (MD 8.71%, 95% CI 4.64 to 12.78, P<0.001) (**Figure 3d**), with moderate heterogeneity (I²=36.3%). Subgroup analysis showed a significant improvement with SGLT2i (3 studies, MD 8.23%, 95% CI 4.13 to 12.32, P<0.001), whereas the effect of GLP-1RA did not reach statistical significance (3 studies, MD 9.81%, 95% CI −0.96 to 20.58, P=0.07). No significant difference was observed between subgroups (P=0.79). Egger’s test showed no evidence of small-study effects (P=0.229) (**Supplementary Figure 2e**).

#### 3.4.3 Glycemic control parameters

##### 3.4.3.1 FPG

Compared with IIT alone, IIT combined with non-insulin antihyperglycemic agents significantly reduced FPG (MD −1.35mmol/L, 95% CI −1.81 to −0.89, P<0.001), with substantial heterogeneity (I²=96.8%) (**Supplementary Figure 1a**). Subgroup analysis by drug class showed significant reductions with DPP-4i (5 studies, MD −0.44mmol/L, 95% CI −0.67 to −0.21, P<0.001), GLP-1RA (5 studies, MD −0.93mmol/L, 95% CI −1.32 to −0.54, P<0.001), metformin (9 studies, MD −2.20mmol/L, 95% CI −3.04 to −1.36, P<0.001), and SGLT2i (6 studies, MD −1.14mmol/L, 95% CI −2.09 to −0.20, P=0.02). A significant difference was observed between drug subgroups (P<0.001).

Given the substantial heterogeneity within the SGLT2i subgroup, a further exploratory analysis according to IIT modality was performed (**Supplementary Figure 1b**). FPG was significantly reduced in studies using CSII (2 studies, MD −1.11mmol/L, 95% CI −2.05 to −0.17, P=0.02), whereas the effect was not statistically significant in studies using MDI (4 studies, MD −1.16mmol/L, 95% CI −2.59 to 0.27, P=0.11). No significant difference was observed between the CSII and MDI subgroups (P=0.95), and substantial residual heterogeneity remained, particularly in the MDI subgroup (I²=96.2%). Egger’s test showed no evidence of small-study effects in the overall FPG analysis (P=0.451) (**Supplementary Figure 2f**) or in the SGLT2i analysis stratified by IIT modality (P=0.436) (**Supplementary Figure 2g**).

##### 3.4.3.2 PPG-2h

Compared with IIT alone, IIT combined with non-insulin antihyperglycemic agents significantly reduced PPG-2h (MD −2.35mmol/L, 95% CI −3.01 to −1.69, P<0.001), with substantial heterogeneity (I²=95.7%) (**Supplementary Figure 1c**). Subgroup analysis by drug class showed significant reductions with DPP-4i (5 studies, MD −1.37mmol/L, 95% CI −2.18 to −0.57, P<0.001), GLP-1RA (4 studies, MD −1.30mmol/L, 95% CI −1.84 to −0.76, P<0.001), metformin (8 studies, MD −2.92mmol/L, 95% CI −3.72 to −2.13, P<0.001), whereas, based on very low certainty evidence, SGLT2i might be associated with a reduction in PPG-2h (3 studies, MD −3.85mmol/L, 95% CI −6.67 to −1.03, P=0.01). A significant difference was observed between drug subgroups (P<0.001). Egger’s test showed no evidence of small-study effects (P=0.525) (**Supplementary Figure 2h**).

##### 3.4.3.3 HbA1c

Compared with IIT alone, IIT combined with non-insulin antihyperglycemic agents significantly reduced HbA1c (MD −1.04%, 95% CI −1.60 to −0.48, P<0.001), with substantial heterogeneity (I²=95.3%) (**Supplementary Figure 1d**). Subgroup analysis by drug class showed a significant reduction with GLP-1RA (4 studies, MD −0.73%, 95% CI −1.01 to −0.45, P<0.001) and SGLT2i (5 studies, MD −1.50%, 95% CI −2.42 to −0.58, P=0.001), whereas metformin showed no significant effect (1 study, MD 0.10%, 95% CI −0.52 to 0.72, P=0.75). A significant difference was observed between drug subgroups (P=0.01).

Given the substantial heterogeneity within the SGLT2i subgroup, a further exploratory subgroup analysis according to patient category was performed(**Supplementary Figure 1e**). HbA1c was significantly reduced in both P1 (2 studies, MD −0.73%, 95% CI −1.42 to −0.03, P=0.04) and P2 patients (2 studies, MD −1.74%, 95% CI −3.42 to −0.07, P=0.04), although substantial heterogeneity remained, particularly in P2 (I²=96.3%). One study with an unclear patient category also showed a significant reduction (MD −2.60%, 95% CI −2.97 to −2.23, P<0.001). A significant difference was observed among patient-category subgroups (P<0.001). Egger’s test showed no evidence of small-study effects in the overall HbA1c analysis (P=0.457) (**Supplementary Figure 2i**) or in the SGLT2i patient-category analysis (P=0.175) (**Supplementary Figure 2j**).

##### 3.4.3.4 MBG

Compared with IIT alone, IIT combined with non-insulin antihyperglycemic agents significantly reduced MBG (MD −0.76mmol/L, 95% CI −1.10 to −0.41, P<0.001), with substantial heterogeneity (I²=80.1%) (**Supplementary Figure 1f**). Subgroup analysis by drug class showed significant reductions with DPP-4i (5 studies, MD −0.86mmol/L, 95% CI −1.21 to −0.51, P<0.001), GLP-1RA (5 studies, MD −0.88mmol/L, 95% CI −1.60 to −0.17, P=0.02), and metformin (1 study, MD −1.27mmol/L, 95% CI −1.63 to −0.91, P<0.001), whereas SGLT2i showed no significant effect (2 studies, MD −0.02mmol/L, 95% CI −1.14 to 1.10, P=0.97). No significant difference was observed between drug subgroups (P=0.13).

Additional sensitivity analysis stratified by data type showed a statistically significant difference between endpoint-value and change-from-baseline estimates within the GLP-1RA subgroup (endpoint values: 4 studies, MD −0.49 mmol/L, 95% CI −0.96 to −0.02; change from baseline: 1 study, MD −1.98 mmol/L, 95% CI −2.75 to −1.21; P for subgroup difference=0.0012). However, because the change-from-baseline subgroup comprised only one study, this finding was considered exploratory and should not be interpreted as definitive evidence that data type modified the treatment effect (**Supplementary Material 4**).

Sensitivity analyses were performed within subgroups where applicable. In the DPP-4i subgroup, exclusion of the study with unclear IIT modality yielded a similar significant effect (MD −0.89mmol/L, 95% CI −1.35 to −0.43, P<0.001). In the GLP-1RA subgroup, exclusion of the study with unclear IIT modality produced a similar result (MD −0.87mmol/L, 95% CI −1.64 to −0.10, P=0.03), while exclusion of the MDI study attenuated the pooled effect but remained statistically significant (MD −0.49mmol/L, 95% CI −0.95 to −0.02, P=0.04). Egger’s test showed no evidence of small-study effects (P=0.558) (**Supplementary Figure 2k**).

#### 3.4.4 Insulin dosage metrics

##### 3.4.4.1 TDD

Compared with IIT alone, IIT combined with non-insulin antihyperglycemic agents significantly reduced weight-adjusted total daily insulin dose (MD −0.09U/kg/day, 95% CI −0.14 to −0.04, P<0.001), with substantial heterogeneity (I²=73.5%) (**Supplementary Figure 1g**). Subgroup analysis by drug class showed significant reductions with DPP-4i (2 studies, MD −0.05U/kg/day, 95% CI −0.10 to −0.01, P=0.03), GLP-1RA (5 studies, MD −0.10U/kg/day, 95% CI −0.19 to −0.02, P=0.02), whereas data from a single trial suggested that metformin might be associated with a reduction in weight-adjusted total daily insulin dose (1 study, MD −0.12U/kg/day, 95% CI −0.21 to −0.03, P=0.01), and data from another single trial suggested that SGLT2i might be associated with a reduction (1 study, MD −0.07U/kg/day, 95% CI −0.12 to −0.02, P<0.01). Heterogeneity was negligible in the DPP-4i subgroup (I²≈0%) but substantial in the GLP-1RA subgroup (I²=81.1%). No significant difference was observed between drug subgroups (P=0.52). Egger’s test showed no evidence of small-study effects (P=0.797) (**Supplementary Figure 2l**).

##### 3.4.4.2 Basal insulin dose

A total of 4 studies reported basal insulin dose (U/kg). Overall, combination therapy did not significantly reduce basal insulin requirements compared with IIT alone (MD −0.02U/kg/day, 95% CI −0.05 to 0.00, P=0.09), with moderate heterogeneity (I²=41.0%) (**Supplementary Figure 1h**). Subgroup analysis by drug class showed no significant effect with GLP-1RA (3 studies, MD −0.01U/kg/day, 95% CI −0.06 to 0.04, P=0.64), with moderate heterogeneity (I²=61.2%), whereas data from a single trial suggested that SGLT2i might be associated with a reduction in basal insulin dose (1 study, MD −0.04U/kg/day, 95% CI −0.07 to −0.01, P=0.01). No significant difference was observed between drug subgroups (P=0.32). Egger’s test showed no evidence of small-study effects (P=0.227) (**Supplementary Figure 2m**).

##### 3.4.4.3 Bolus insulin dose

A total of 4 studies reported bolus insulin dose (U/kg). Overall, combination therapy significantly reduced bolus insulin requirements compared with IIT alone (MD −0.04U/kg/day, 95% CI −0.05 to −0.02, P<0.001), with negligible heterogeneity (I²=0.07%) (**Supplementary Figure 1i**). Subgroup analysis by drug class showed a significant reduction with GLP-1RA (3 studies, MD −0.04U/kg/day, 95% CI −0.06 to −0.02, P<0.001), with virtually no heterogeneity (I²=0.09%). Data from a single trial suggested that SGLT2i might also be associated with a reduction in bolus insulin dose (1 study, MD −0.03U/kg/day, 95% CI −0.06 to 0.00, P=0.03). No significant difference was observed between drug subgroups (P=0.44). Egger’s test showed no evidence of small-study effects (P=0.497) (**Supplementary Figure 2n**).

#### 3.4.5 Additional glycemic outcomes

##### 3.4.5.1 Body weight

Compared with IIT alone, IIT combined with non-insulin antihyperglycemic agents showed a borderline significant reduction in body weight (MD −2.12kg, 95% CI −4.21 to −0.02, P=0.05), with substantial heterogeneity (I²=99.4%) (**Supplementary Figure 1j**). Subgroup analysis by drug class showed significant weight reductions with GLP-1RA (5 studies, MD −3.48kg, 95% CI −6.61 to −0.35, P=0.03) and metformin (4 studies, MD −2.08kg, 95% CI −2.47 to −1.70, P<0.001). In contrast, no significant effects were observed with DPP-4i (3 studies, MD −0.13kg, 95% CI −0.42 to 0.16, P=0.38) or SGLT2i (4 studies, MD −3.04kg, 95% CI −10.67 to 4.59, P=0.43). Heterogeneity was negligible in the metformin subgroup (I²=0%) and low in the DPP-4i subgroup (I²=19.3%), but remained substantial for GLP-1RA (I²=96.4%) and SGLT2i (I²=94.1%). Sensitivity analyses suggested that treatment duration may partly explain the heterogeneity within the GLP-1RA subgroup. After excluding the shorter-duration studies by Luo_2025 (4 weeks) and Li_2019 (2 weeks), heterogeneity decreased to below 50%. In contrast, exclusion of Lin_2022 from the SGLT2i subgroup did not materially reduce heterogeneity, suggesting that treatment duration was unlikely to be the major source of heterogeneity in this subgroup. A sensitivity analysis stratified by data type showed a statistically significant difference between endpoint-value and change-from-baseline estimates within the metformin subgroup (endpoint value: 1 study, MD 2.30 kg, 95% CI −2.10 to 6.70; change from baseline: 3 studies, MD −2.12 kg, 95% CI −2.50 to −1.73; P for subgroup difference=0.0498). However, because the endpoint-value subgroup was represented by only one study, this finding was considered exploratory and should not be interpreted as definitive evidence that the method of outcome expression modified the treatment effect (**Supplementary Material 4**). For body weight, the pooled effect in the SGLT2i subgroup was not statistically significant (MD −3.04 kg, 95% CI −10.67 to 4.59), with substantial heterogeneity (I²=94.1%). Du 2019 showed a markedly larger treatment effect than the other studies (MD −13.20 kg). Leave-one-out sensitivity analysis showed that exclusion of Du 2019 substantially reduced the between-study heterogeneity(I2 66.73), indicating that this study was an important contributor to the observed heterogeneity (**Supplementary Material 3**). A significant difference was observed between drug subgroups (P<0.001). Egger’s test showed no evidence of small-study effects (P=0.197) (**Supplementary Figure 2o**).

##### 3.4.5.2 HOMA-IR

Compared with IIT alone, IIT combined with non-insulin antihyperglycemic agents significantly reduced HOMA-IR (MD −0.85, 95% CI −1.34 to −0.36, P<0.001), with substantial heterogeneity (I²=98.5%) (**Supplementary Figure 1k**). Subgroup analysis by drug class showed significant reductions with GLP-1RA (4 studies, MD −0.65, 95% CI −1.20 to −0.11, P=0.02), metformin (3 studies, MD −0.80, 95% CI −1.40 to −0.20, P=0.01), and SGLT2i (1 study, MD −2.70, 95% CI −2.99 to −2.41, P<0.001), whereas the effect of DPP-4i was not statistically significant (2 studies, MD −0.42, 95% CI −0.98 to 0.14, P=0.14). A significant difference was observed between drug subgroups (P<0.001).

Given the substantial heterogeneity within the GLP-1RA subgroup, a further exploratory subgroup analysis according to patient category was performed(**Supplementary Figure 1l**). The pooled effect was not statistically significant in either P1 (2 studies, MD −0.83, 95% CI −1.89 to 0.23, P=0.12) or P2 patients (2 studies, MD −0.47, 95% CI −1.08 to 0.15, P=0.14), and substantial heterogeneity remained in both subgroups (I²=96.1% and 95.6%, respectively). No significant difference was observed between P1 and P2 subgroups (P=0.56), indicating that patient category did not explain the observed heterogeneity. Egger’s test showed no evidence of small-study effects in the overall HOMA-IR analysis (P=0.875) (**Supplementary Figure 2p**) or in the GLP-1RA patient-category analysis (P=0.524) (**Supplementary Figure 2q**).

### 3.5 Safety and tolerability

#### 3.5.1 Hypoglycemia

Compared with IIT alone, IIT combined with non-insulin antihyperglycemic agents was associated with a lower risk of hypoglycemia (RR 0.70, 95% CI 0.50 to 0.96, P=0.03), with no observed heterogeneity (I²=0%) (**Supplementary Figure 1m**). IIT plus DPP4i may reducethe risk of hypoglycemia (4 studies, RR 0.53, 95% CI 0.31 to 0.91, P=0.02). Very-low-certainty evidence suggested a lower risk of hypoglycemia with SGLT2i (5 studies, RR 0.30, 95% CI 0.09 to 0.94, P=0.04). In contrast, no significant effects were observed with GLP-1RA (3 studies, RR 1.00, 95% CI 0.59 to 1.68, P=1.00) or metformin (4 studies, RR 0.83, 95% CI 0.35 to 1.93, P=0.66). No significant difference was observed between drug subgroups (P=0.17). Egger’s test showed no evidence of small-study effects (P=0.092) (**Supplementary Figure 2r**).

#### 3.5.2 GI-AE

Compared with IIT alone, IIT combined with non-insulin antihyperglycemic agents was associated with a higher risk of GI-AEs (RR 2.79, 95% CI 1.07 to 7.27, P=0.04), with moderate heterogeneity (I²=57.3%)(**Supplementary Figure 1n**). Subgroup analysis by drug class showed a significantly increased risk of GI-AEs with GLP-1RA (7 studies, RR 5.28, 95% CI 1.81 to 15.40, P=0.002), whereas no significant effect was observed with metformin (3 studies, RR 0.87, 95% CI 0.27 to 2.85, P=0.82). A significant difference was observed between drug subgroups (P=0.03). Egger’s test indicated evidence of small-study effects (P=0.010), whereas Harbord’s test showed no evidence of small-study effects (P=0.854) (**Supplementary Figure 2s**).

Trial-level definitions, ascertainment methods, assessment windows, and reporting of hypoglycemia, gastrointestinal adverse events (GI-AEs), diabetic ketoacidosis (DKA), and treatment discontinuation are summarized in **Supplementary Table 2**.

The definition and completeness of hypoglycemia reporting varied across trials. Some trials explicitly prespecified an operational definition of hypoglycemia, most commonly using a blood glucose threshold of ≤3.9 mmol/L (70 mg/dL). Several studies further defined severe hypoglycemia using a lower glucose threshold or the requirement for assistance from another person. In contrast, some studies reported hypoglycemic events without providing an explicit glucose threshold or operational definition. Trials using continuous glucose monitoring (CGM) or flash glucose monitoring (FGM) sometimes assessed hypoglycemia primarily using metrics such as time below range (TBR), area under/below a prespecified glucose threshold, or sensor-detected low-glucose readings rather than conventional participant-level hypoglycemic event counts. When neither a hypoglycemia definition nor a clearly identifiable ascertainment criterion was available from the original publication, the corresponding item was recorded as NR in **Supplementary Table 2** rather than being inferred retrospectively from the study results. Trial-specific definitions, assessment windows, and event reporting are detailed in **Supplementary Table 2**.

Reporting of GI-AEs was even less standardized. Trials did not consistently report a composite outcome of “any GI-AE”; instead, individual symptoms such as nausea, vomiting, diarrhea, abdominal pain or fullness, anorexia, and nonspecific digestive symptoms were reported. Accordingly, the specific gastrointestinal symptom categories reported in each original study were retained in **Supplementary Table 2** rather than being treated as an identical composite outcome across trials.

DKA was rarely explicitly reported as a treatment-emergent safety outcome. Among the trials with explicit DKA assessment, Fushimi et al. defined DKA as a β-hydroxybutyrate concentration ≥3.0 mmol/L and reported no DKA events in either group (0/18 vs 0/17). In several other SGLT2 inhibitor trials, DKA was mentioned only as a baseline exclusion criterion, without a prespecified treatment-emergent DKA definition or active ketone-monitoring strategy.

Reporting of treatment discontinuation also varied across studies. Where available, discontinuations were generally uncommon, and many studies reported no participant withdrawals. Reported reasons for discontinuation were frequently unrelated to adverse events and included withdrawal of consent, protocol deviation, sensor detachment, poor adherence, loss to follow-up, and failure to achieve the prespecified glycemic target. In contrast, Lind et al. reported that two liraglutide-treated participants discontinued the intervention because of gastrointestinal symptoms. **Supplementary Table 2** further distinguishes all-cause withdrawal, adverse-event-related discontinuation, and other post-randomization exclusions.

## 4 Discussion

This systematic review and meta-analysis extends previous evidence by evaluating not only conventional glycemic control but also glycemic variability and clinically relevant drug-specific outcomes associated with the addition of non-insulin antihyperglycemic agents to intensive insulin therapy (IIT) in adults with type 2 diabetes mellitus. Among the primary glycemic-variability outcomes, relatively consistent evidence supported reductions in MAGE and LAGE with DPP-4i and in SDBG with SGLT2i, whereas findings for other drug–outcome combinations were more uncertain.

For the prespecified secondary glycemic outcomes, reductions in FPG and PPG-2h were observed with DPP-4i, GLP-1RA, metformin, and SGLT2i. Reductions in HbA1c were also observed with GLP-1RA and SGLT2i, with the evidence being more consistent for GLP-1RA. Evidence regarding insulin requirements and other metabolic outcomes was less definitive.

*Post hoc* exploratory analyses suggested that SGLT2i may improve TIR, longer-duration GLP-1RA therapy may be associated with greater weight reduction, DPP-4i may reduce the risk of hypoglycemia, and GLP-1RA may increase the risk of gastrointestinal adverse events. However, these findings should be interpreted cautiously because of their exploratory nature and, for several outcomes, imprecision or substantial heterogeneity.

Overall, the effects of adding non-insulin antihyperglycemic agents during IIT appear to be drug- and outcome-specific rather than uniform across drug classes. Conventional markers such as HbA1c, FPG, and PPG-2h primarily reflect average or point-specific glycemic exposure and do not fully characterize short-term glucose fluctuations. In contrast, TIR, SDBG, MAGE, and LAGE provide complementary information regarding the stability and amplitude of daily glucose excursions. Previous evidence on IIT combination strategies has focused predominantly on HbA1c, whereas glycemic-variability outcomes have been less comprehensively evaluated. The moderate-certainty improvement in TIR and SDBG observed with IIT plus SGLT2i therefore suggests that adjunctive therapy may improve not only mean glycemic control but also the stability of the glucose profile. Similarly, the reduction in LAGE with IIT plus DPP-4i indicates attenuation of large glucose excursions, while the accompanying reduction in MAGE should be interpreted more cautiously because of the lower certainty of evidence.

The observed drug-specific patterns are biologically plausible given the complementary mechanisms of these agents and exogenous insulin. DPP-4i enhances endogenous incretin activity, thereby promoting glucose-dependent insulin secretion and suppressing glucagon secretion (47–49). This glucose-dependent action may help smooth excessive glucose excursions while limiting hypoglycemia, providing a plausible explanation for the observed improvements in LAGE and MAGE and the lower hypoglycemia risk. SGLT2i, in contrast, lowers glucose through an insulin-independent increase in urinary glucose excretion, which may reduce sustained glucose exposure and contribute to improvements in TIR and SDBG without requiring additional insulin. GLP-1RA also acts through glucose-dependent insulinotropic and glucagon-suppressive mechanisms and may reduce insulin requirements through improved glycemic control and reduced caloric intake. These mechanistic differences may explain why no single adjunctive drug class demonstrated superiority across all outcomes.

The insulin-sparing effects are also clinically relevant. IIT plus DPP-4i and IIT plus GLP-1RA both reduced total insulin requirements, although the certainty of evidence was low. Reduced insulin exposure may potentially lessen treatment burden and mitigate insulin-associated adverse effects. In addition, DPP-4i was associated with a lower risk of hypoglycemia, which is consistent with its glucose-dependent mechanism of action. Conversely, the reduction in insulin requirements with GLP-1RA should be balanced against the increased risk of gastrointestinal adverse events.

The body-weight findings require particular caution. Although longer-duration IIT plus GLP-1RA was associated with greater weight reduction, this finding was derived from a *post hoc* exploratory subgroup analysis and was supported by low-certainty evidence. The observed pattern is biologically plausible because GLP-1RA reduces appetite, delays gastric emptying, and lowers energy intake (50); however, the available data are insufficient to establish a specific duration threshold for clinically meaningful weight loss. Rather than demonstrating a definitive duration-dependent effect, these findings should be regarded as hypothesis-generating and warrant confirmation in prospective trials with prespecified treatment-duration strata.Previous monotherapy trials demonstrated early weight reduction: LEAD-3 showed significant weight loss within 16 weeks that persisted through 52 weeks (51), while SUSTAIN 1 reported early-onset, sustained reduction over 30 weeks (52).

GLP-1RA formulation and administration route also influence outcomes. While Ke et al. (34) reported no weight difference with CSII plus liraglutide (0.6 mg/d) at 2 weeks, and Li et al. (35) found neutral effects with exenatide (5-10 μg BID) at 5 weeks, but Luo et al. (39) demonstrated significant weight reduction at 4 weeks using beinaglutide via continuous subcutaneous GLP-1RA infusion (CSGI). Differences in formulation, dose, and route of administration may have contributed to between-study variation.

## 5 Limitations

Several limitations should be acknowledged. First, substantial heterogeneity remained for several outcomes despite subgroup and sensitivity analyses, likely reflecting differences in patient characteristics, IIT regimens, treatment duration, drug class, and outcome measurement. Second, some drug-specific subgroup analyses included only a small number of trials, limiting precision and the reliability of interaction testing. Third, differences in the definitions and measurement methods of glycemic-variability indices may have reduced comparability across studies. Fourth,Many included studies had at least some risk-of-bias concerns, which, together with inconsistency and imprecision, contributed to low or very low certainty for several findings. Fifth, HOMA-β could not be quantitatively synthesized because of differences in calculation methods and reporting units. Finally, although no language restrictions were applied and both international and major Chinese databases were searched, residual language-related and database-coverage bias cannot be completely excluded. We searched PubMed, Embase, the Cochrane Library, Web of Science, ClinicalTrials.gov, CNKI, Wanfang, VIP, and CMA, which provided broad coverage of both English- and Chinese-language literature. Nevertheless, eligible studies indexed only in databases not searched, unpublished studies, grey literature, or reports with limited indexing may have been missed. Therefore, although our comprehensive multilingual search strategy was designed to minimize language and database-selection bias, some residual risk of incomplete retrieval remains.

## 6 Conclusion

In adults with T2DM receiving intensive insulin therapy, the addition of non-insulin antihyperglycemic agents may provide additional benefits in glycemic variability and overall glycemic control; however, the magnitude and consistency of these effects varied across drug classes and outcomes. Among the primary glycemic-variability outcomes, relatively consistent evidence supported reductions in MAGE and LAGE with DPP-4i and in SDBG with SGLT2i, whereas findings for other drug–outcome combinations were more uncertain.

For the secondary glycemic outcomes, DPP-4i, GLP-1RA, metformin, and SGLT2i were associated with reductions in FPG and PPG-2h, although substantial between-study heterogeneity in some drug subgroups reduced confidence in these effect estimates. Reductions in HbA1c were also observed with GLP-1RA and SGLT2i, with the evidence being more consistent for GLP-1RA. Evidence regarding insulin requirements and other metabolic outcomes remained less definitive and should be interpreted cautiously.

*Post hoc* exploratory analyses suggested that SGLT2i may improve TIR, while longer-duration GLP-1RA therapy may reduce body weight. In addition, DPP-4i may reduce hypoglycemia, whereas GLP-1RA may increase gastrointestinal adverse events. However, because these analyses were *post hoc* and several outcomes were limited by imprecision or substantial heterogeneity, these findings should not be considered confirmatory. Overall, the findings suggest a potential clinical role for selected non-insulin antihyperglycemic agents as adjuncts to intensive insulin therapy, while highlighting the need for larger, rigorously designed trials using standardized glycemic-variability and safety outcomes to further clarify drug-specific effects.

## Data Availability

The original contributions presented in the study are included in the article/**Supplementary Material**. Further inquiries can be directed to the corresponding author.

## Supplementary material

Supplementary Figure 1 Forest plots of glycemic, metabolic, insulin-dose, and safety outcomes. **(a)** Fasting plasma glucose (FPG); **(b)** FPG stratified by intensive insulin therapy (IIT) administration method; **(c)** 2-hour postprandial glucose (PPG-2h); (d) glycated hemoglobin (HbA1c); **(e)** HbA1c stratified by patient category; **(f)** mean blood glucose (MBG); **(g)** total daily insulin dose (TDD); **(h)** basal insulin dose; **(i)** bolus insulin dose; **(j)** body weight; **(k)** homeostasis model assessment of insulin resistance (HOMA-IR); **(l)** HOMA-IR stratified by patient category; **(m)** hypoglycemia; and **(n)** gastrointestinal adverse events (GI-AEs). Pooled estimates were obtained using random-effects models.

Supplementary Figure 2 Funnel plots used to assess small-study effects. **(a)** Standard deviation of blood glucose (SDBG); **(b)** SDBG after trim-and-fill analysis; **(c)** mean amplitude of glycemic excursions (MAGE); **(d)** largest amplitude of glycemic excursions (LAGE); **(e)** time in range (TIR); **(f)** fasting plasma glucose (FPG); **(g)** FPG stratified by intensive insulin therapy (IIT) administration method; **(h)** 2-hour postprandial glucose (PPG-2h); **(i)** glycated hemoglobin (HbA1c); **(j)** HbA1c stratified by patient category; **(k)** mean blood glucose (MBG); **(l)** total daily insulin dose (TDD); **(m)** basal insulin dose; **(n)** bolus insulin dose; **(o)** body weight; **(p)** homeostasis model assessment of insulin resistance (HOMA-IR); **(q)** HOMA-IR stratified by patient category; **(r)** hypoglycemia; and **(s)** gastrointestinal adverse events (GI-AEs). Trim-and-fill analysis was performed for SDBG following evidence of funnel-plot asymmetry.

Supplementary Table 1 Characteristics of the included studies and participants. The table presents country, study design, patient category, allocated participants, age, diabetes duration and treatment history, glycated hemoglobin (HbA1c) eligibility criterion and baseline value, body mass index (BMI), intervention and comparator, and treatment and follow-up duration. Patient categories are P1, previously untreated or treatment-naive; P2, previously diagnosed with inadequate control and initiating corrective intensive insulin therapy (IIT); and P3, previously diagnosed and receiving maintenance IIT. Abbreviations used in the table include randomized controlled trial (RCT), continuous subcutaneous insulin infusion (CSII), multiple daily injections (MDI), extended release (ER), oral glucose tolerance test (OGTT), fasting blood glucose (FBG), neutral protamine Hagedorn (NPH), not reported (NR), and not confirmed (NC). Li 2018 is one pooled report of two underlying RCTs. Beinaglutide is the corrected spelling of benalutide.

Supplementary Table 2 Trial-level definitions, ascertainment, and reporting of safety outcomes. Definitions, assessment windows, and event counts are presented as reported in the original trials and were not retrospectively harmonized. Abbreviations used in the table include continuous glucose monitoring (CGM), flash glucose monitoring (FGM), time below range (TBR), area under the curve (AUC), area over the curve (AOC), blood glucose (BG), plasma glucose (PG), self-monitoring of blood glucose (SMBG), beta-hydroxybutyrate (beta-OHB), adverse event (AE), and not reported (NR).

Supplementary Table 3 Summary of findings and Grading of Recommendations Assessment, Development and Evaluation (GRADE) certainty for drug-specific effects. The population comprises adults with type 2 diabetes mellitus (T2DM) receiving intensive insulin therapy (IIT). The intervention is IIT plus a non-insulin antihyperglycemic agent; the comparator is IIT alone or IIT plus matching placebo. Abbreviations used in the table include confidence interval (CI), mean difference (MD), risk ratio (RR), dipeptidyl peptidase-4 inhibitor (DPP-4i), glucagon-like peptide-1 receptor agonist (GLP-1RA), and sodium-glucose cotransporter-2 inhibitor (SGLT2i).

Supplementary Table 4 Grading of Recommendations Assessment, Development and Evaluation (GRADE) certainty for drug-specific effects on fasting plasma glucose (FPG), 2-hour postprandial glucose (PPG-2h), and glycated hemoglobin (HbA1c). Certainty was assessed across risk of bias, inconsistency, indirectness, imprecision, and publication bias. Randomized trials started at high certainty and were downgraded for serious or very serious concerns. Abbreviations used in the table include confidence interval (CI), mean difference (MD), and I-squared (I²), the proportion of variability attributable to between-study heterogeneity.

Supplementary Material 1 Outcome-level judgments using the Cochrane Risk of Bias 2 (RoB 2) tool. The five domains are D1, randomization process; D2, deviations from intended interventions; D3, missing outcome data; D4, measurement of the outcome; and D5, selection of the reported result. Domain-level and overall judgments, with supporting rationales, are presented for each study, outcome, and data type.

Supplementary Material 2 Detailed Grading of Recommendations Assessment, Development and Evaluation (GRADE) assessment of drug-specific findings. Certainty was assessed across risk of bias, inconsistency, indirectness, imprecision, and publication bias. Randomized trials started at high certainty and were downgraded for serious or very serious concerns. Abbreviations used in the material: confidence interval (CI); dipeptidyl peptidase-4 inhibitor (DPP-4i); gastrointestinal adverse events (GI-AEs); glucagon-like peptide-1 receptor agonist (GLP-1RA); largest amplitude of glycemic excursions (LAGE); mean amplitude of glycemic excursions (MAGE); mean difference (MD); risk ratio (RR); standard deviation of blood glucose (SDBG); sodium-glucose cotransporter-2 inhibitor (SGLT2i); and time in range (TIR).

Supplementary Material 3 Leave-one-out sensitivity analyses by outcome and drug subgroup. Continuous outcomes were analyzed using mean differences (MDs); hypoglycemia and gastrointestinal adverse events (GI-AEs) were analyzed using risk ratios (RRs). For each outcome and drug subgroup, the primary estimate is shown alongside estimates obtained after omitting one study at a time. Reported statistics include the number of studies (k), pooled effect, 95% confidence interval (CI), P value, between-study variance (tau-squared), I-squared (I²), H-squared (H²), Cochran’s Q, degrees of freedom (df), and the P value for Q. Changes in effect, I², and tau-squared are relative to the primary analysis. Leave-one-out analysis was not performed when fewer than three studies contributed. Outcomes include fasting plasma glucose (FPG), glycated hemoglobin (HbA1c), homeostasis model assessment of beta-cell function (HOMA-beta), homeostasis model assessment of insulin resistance (HOMA-IR), mean blood glucose (MBG), and 2-hour postprandial glucose (PPG-2h).

Supplementary Material 4 Random-effects analyses stratified by drug class and data type. Continuous outcomes were summarized as mean differences (MDs; intervention minus control). Standard errors (SEs) allowed for unequal group variances. Restricted maximum likelihood (REML) was used to estimate between-study variance (tau-squared); 95% confidence intervals (CIs) and overall-effect tests used the z method. Where both endpoint values and changes from baseline were available within a drug class, the difference between stratum-specific MDs, its SE and 95% CI, the between-stratum Q statistic, and its P value were reported. Outcomes include fasting plasma glucose (FPG), 2-hour postprandial glucose (PPG-2h), glycated hemoglobin (HbA1c), mean blood glucose (MBG), mean amplitude of glycemic excursions (MAGE), standard deviation of blood glucose (SDBG), largest amplitude of glycemic excursions (LAGE), time in range (TIR), and homeostasis model assessment of insulin resistance (HOMA-IR). Degrees of freedom (df) and not applicable (NA) are also used in the material.

Supplementary Material 5 Full electronic search strategies for the updated literature search. The Cochrane Central Register of Controlled Trials (CENTRAL), Web of Science Core Collection, PubMed, Embase, ClinicalTrials.gov, China National Knowledge Infrastructure (CNKI), Wanfang Data, VIP Database, and the Chinese Medical Association database were searched from inception to 7 November 2025. No language restriction or additional methodological randomized controlled trial (RCT) text filter was applied at retrieval; randomized allocation was verified during eligibility screening. Where an exact day-level limit was unavailable, records were restricted to publication year 2025 or earlier, then records published after 7 November 2025 were excluded manually. The exact executed searches and numbers of records retrieved are provided for each source. Search terms include type 2 diabetes mellitus (T2DM), intensive insulin therapy (IIT), continuous subcutaneous insulin infusion (CSII), multiple daily injections (MDI), dipeptidyl peptidase-4 inhibitor (DPP-4i), sodium-glucose cotransporter-2 inhibitor (SGLT2i), glucagon-like peptide-1 receptor agonist (GLP-1RA), and Medical Subject Headings (MeSH).
