# Supplementary material for: Effects of intensive insulin therapy combined with non-insulin antihyperglycemic agents on glycemic control, variability, and metabolic outcomes in patients with type 2 diabetes mellitus: a systematic review and meta-analysis
Source: Front Endocrinol (Lausanne). 2026 Oct 5;17:1948361. doi: 10.3389/fendo.2026.1948361 (PMC13677461; doi:10.3389/fendo.2026.1948361)
Supplement: Supplementary file 1 [file Image1.pdf]

a

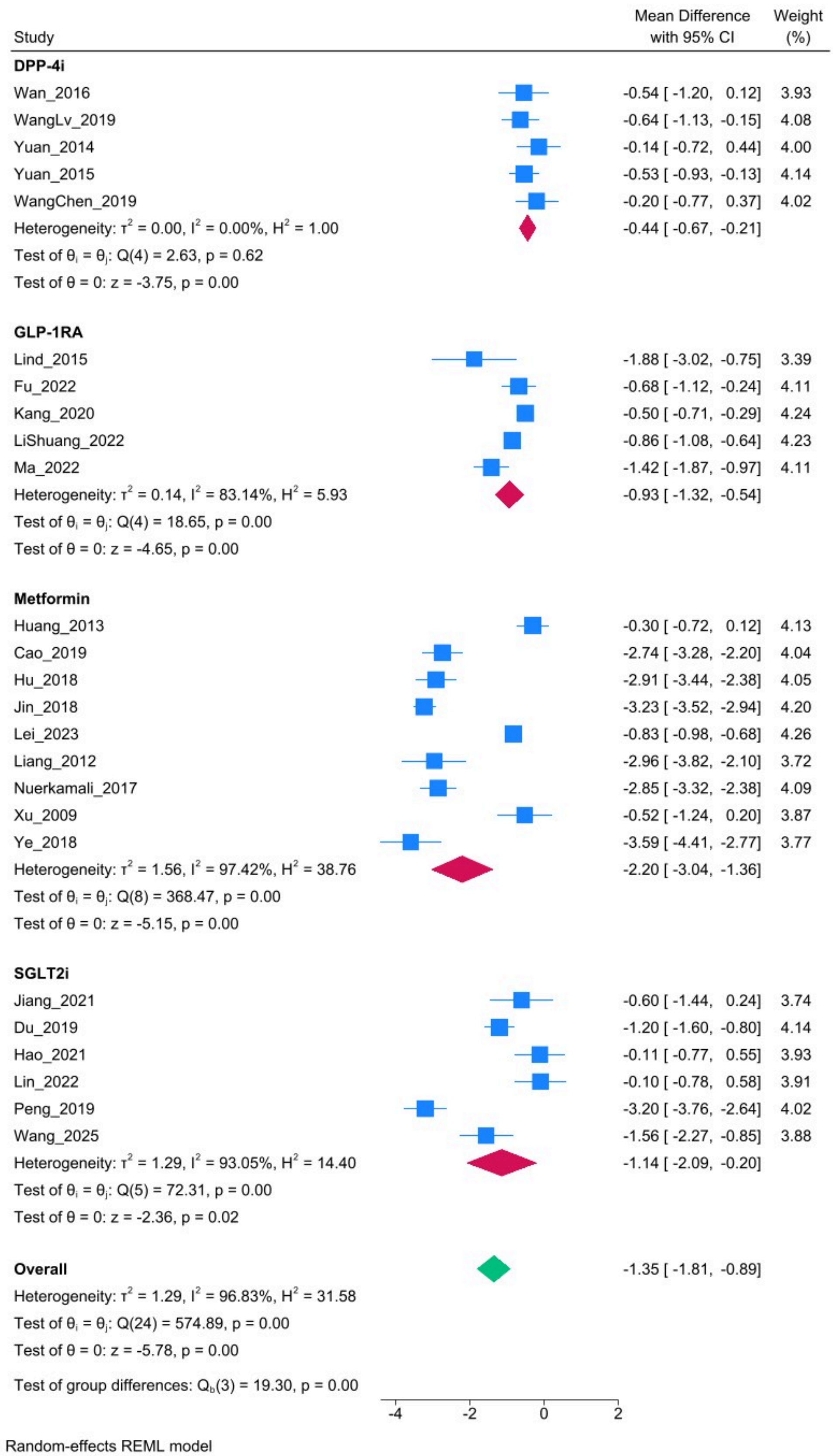

Supplementary Fig. 1. Forest plots of (a) FPG, (b) FPG administration-method subgroup, (c) PPG-2h, (d) HbA1c, (e) HbA1c population subgroup, (f) MBG, (g) TDD, (h) Basal insulin dose, (i) Bolus insulin dose, (j) Body weight, (k) HOMA-IR, (l) HOMA-IR population subgroup, (m) Hypoglycemia, and (n) GI-AE.

b

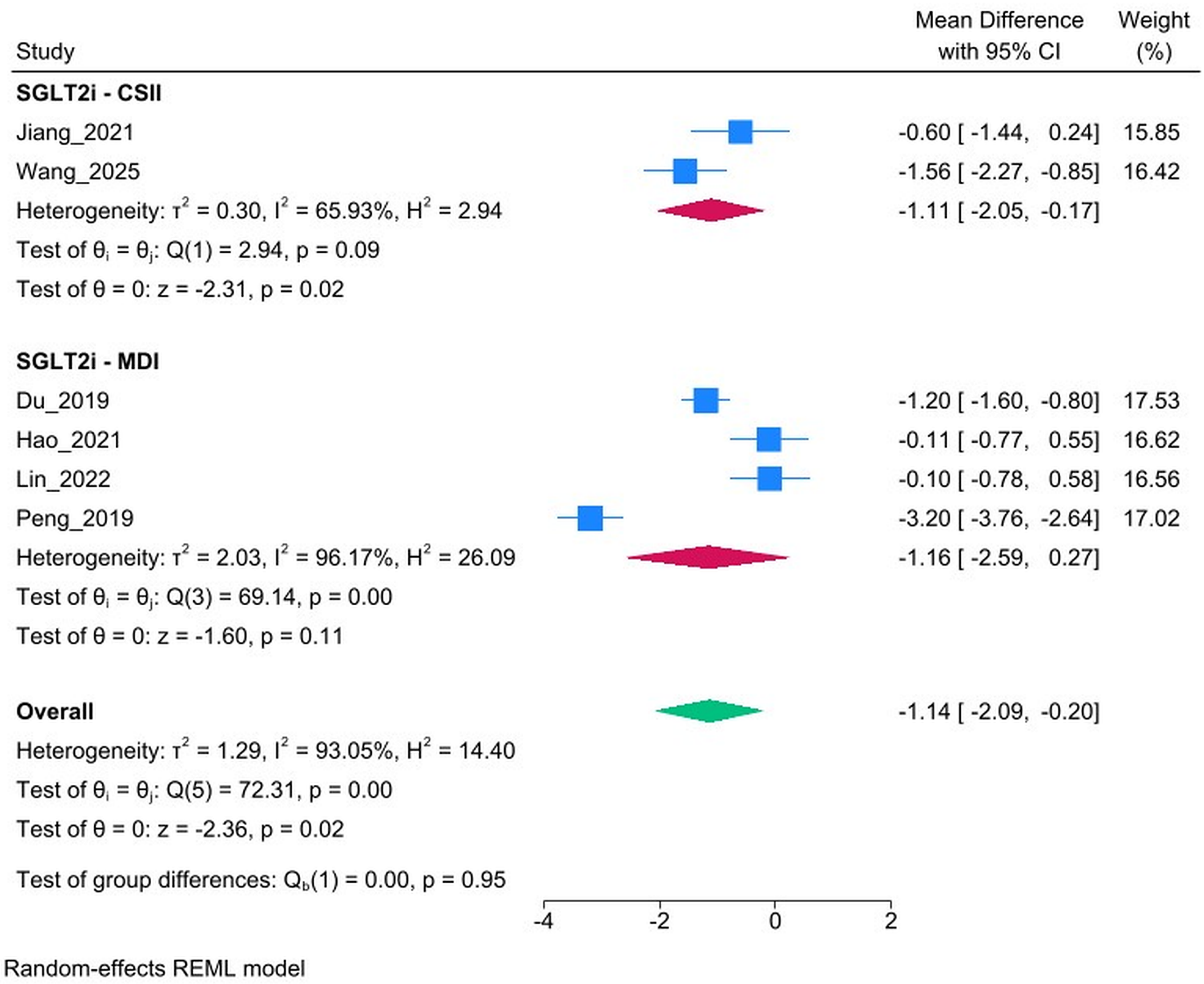

Supplementary Fig. 1 (Continued)

c

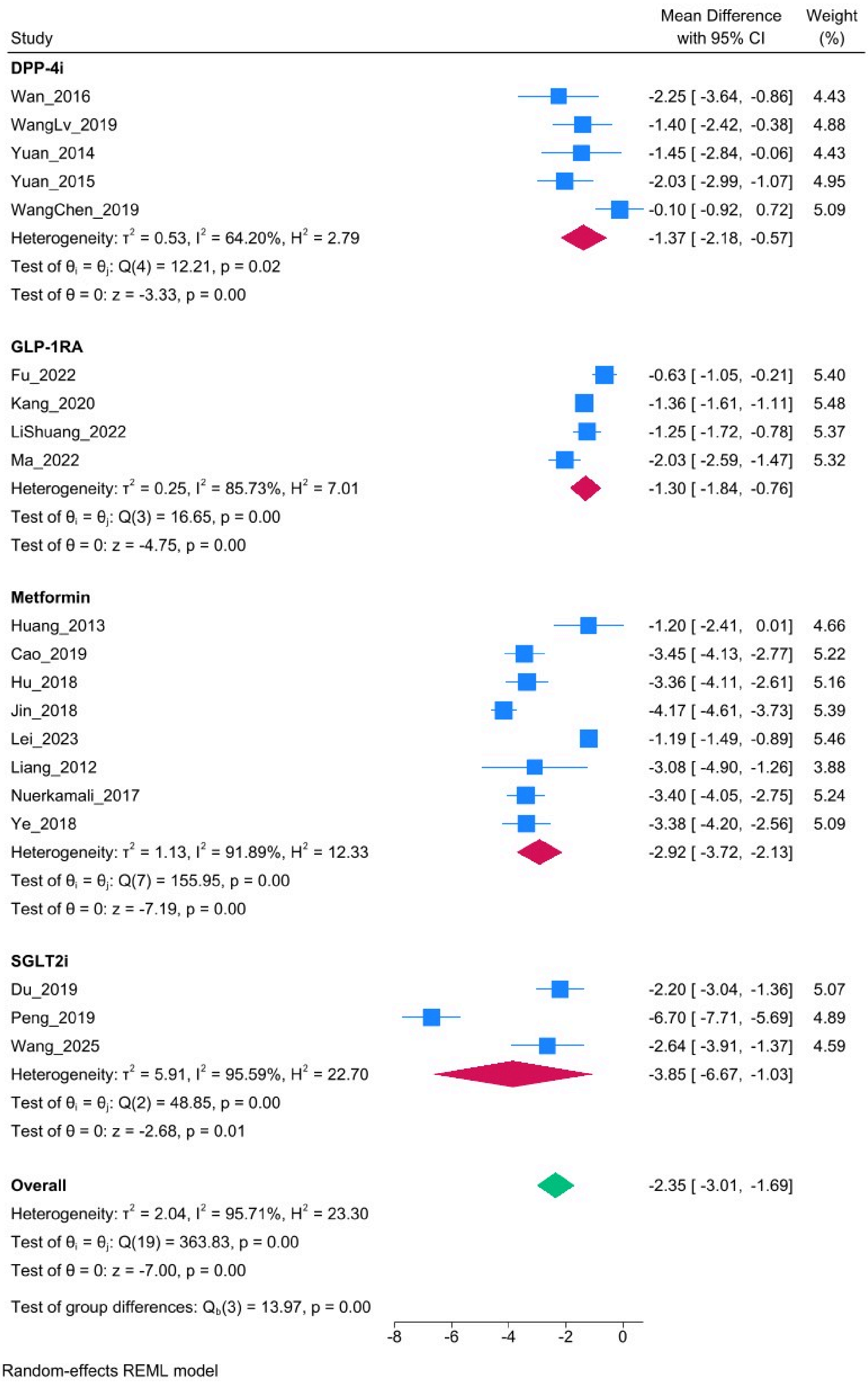

Supplementary Fig. 1 (Continued)

d

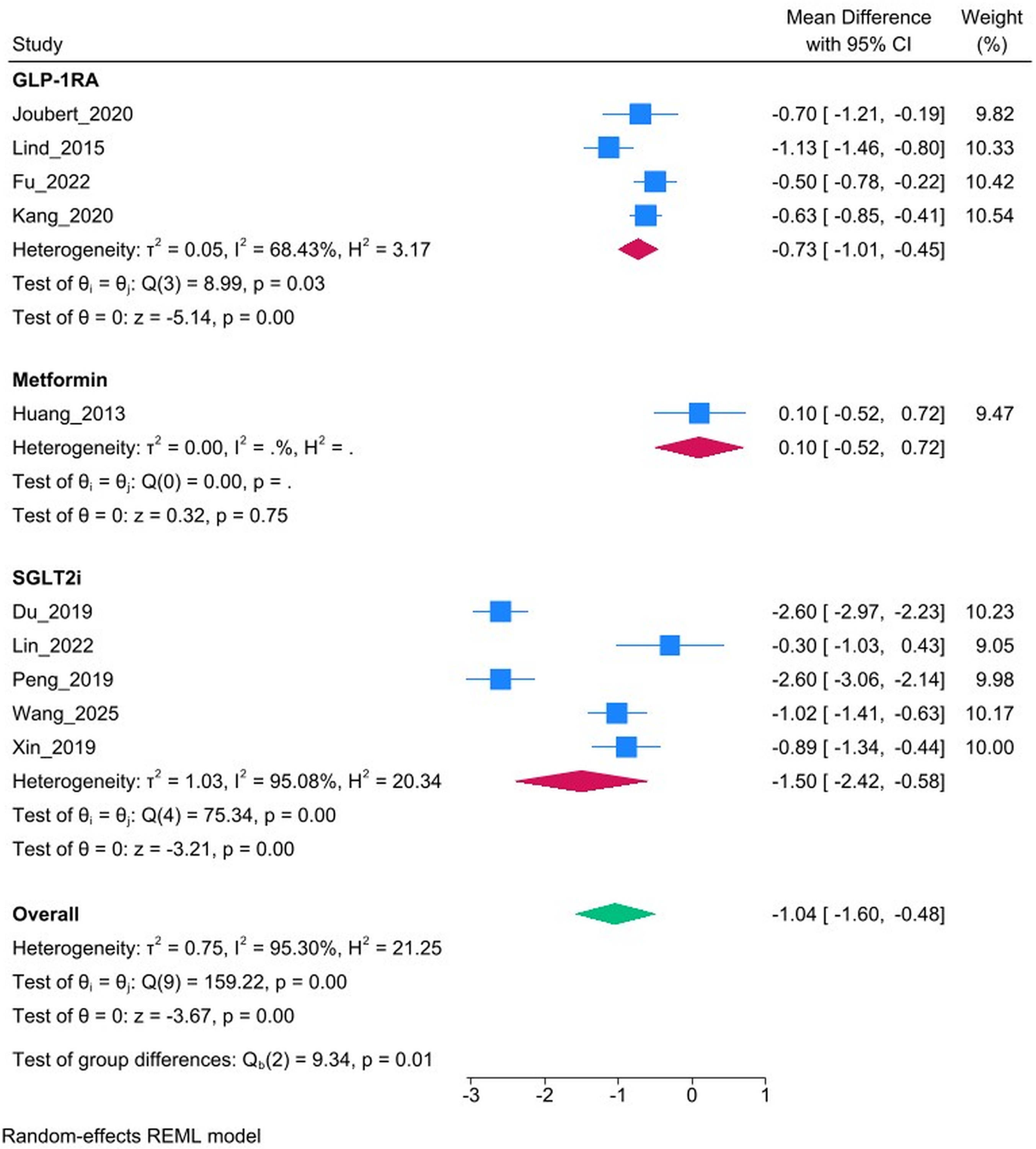

Supplementary Fig. 1 (Continued)

e

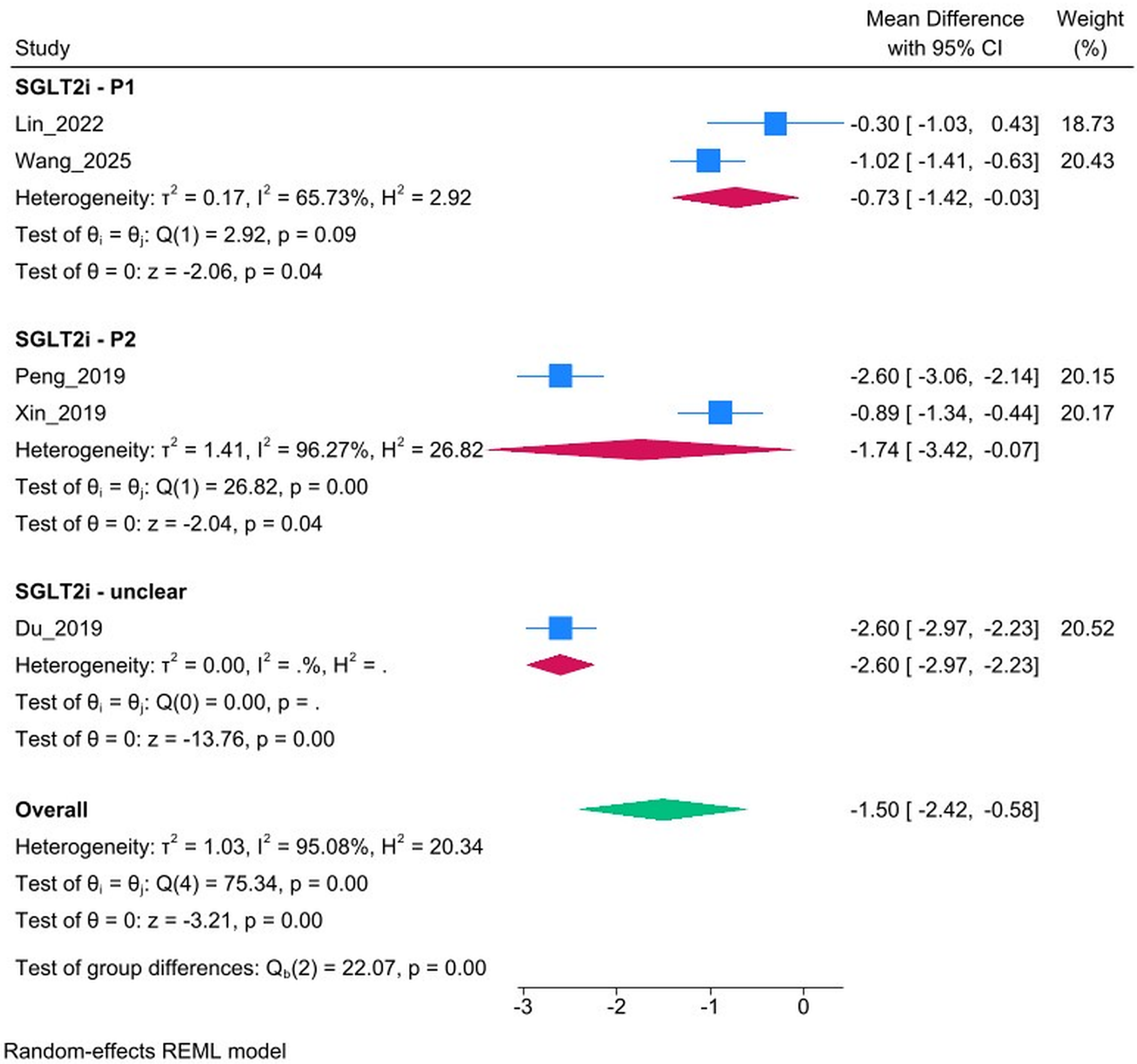

Supplementary Fig. 1 (Continued)

f

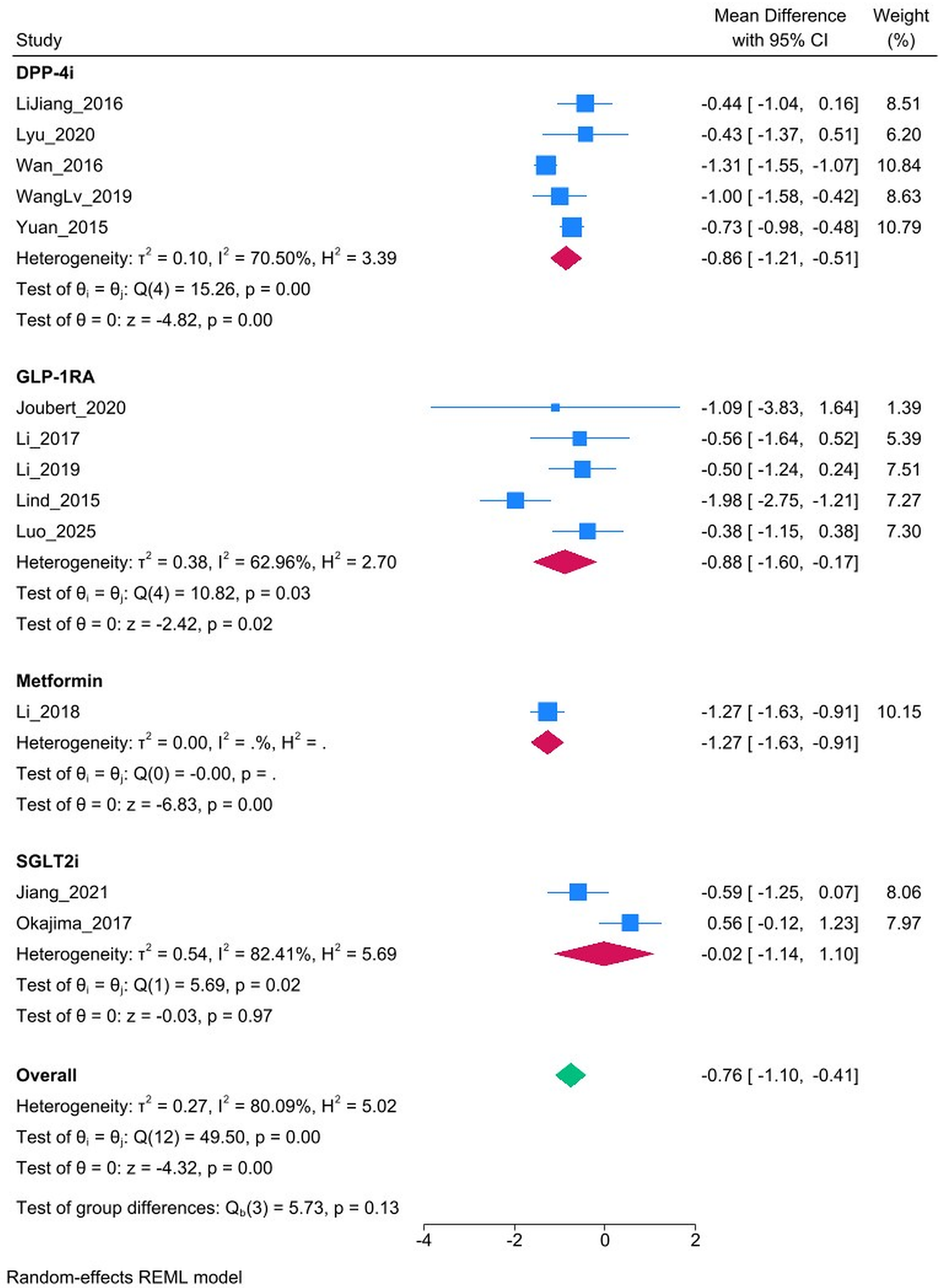

Supplementary Fig. 1 (Continued)

g

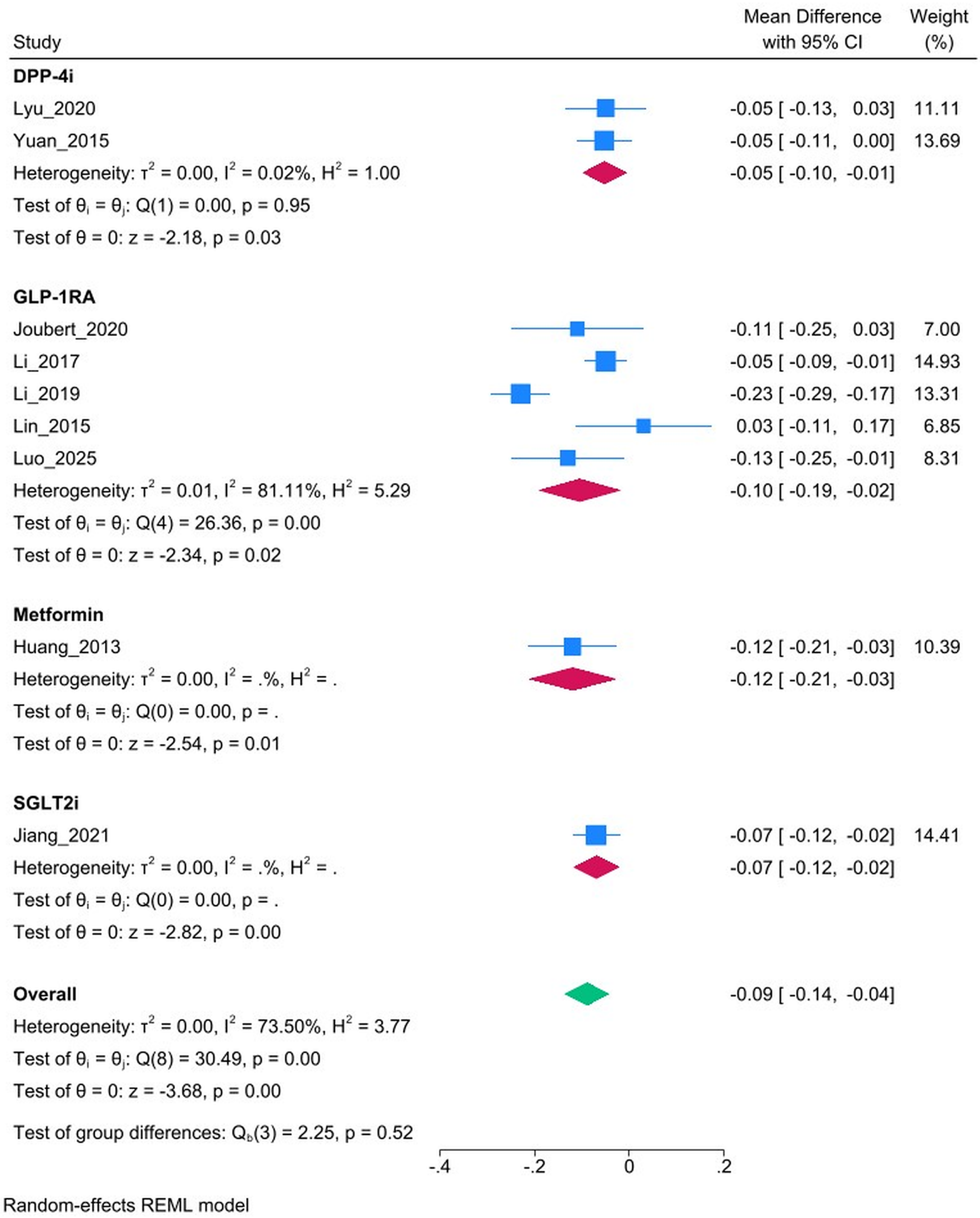

Supplementary Fig. 1 (Continued)

h

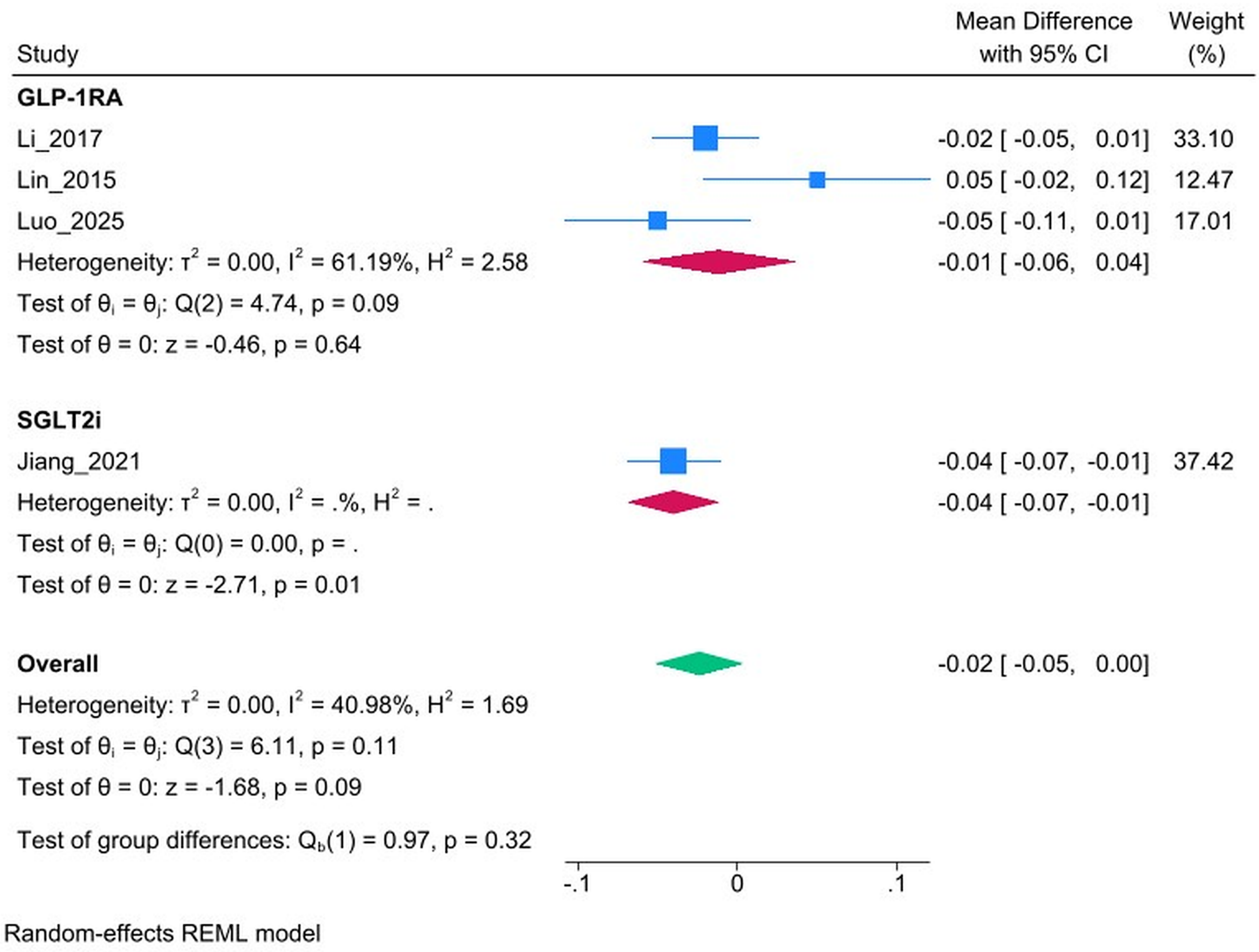

Supplementary Fig. 1 (Continued)

i

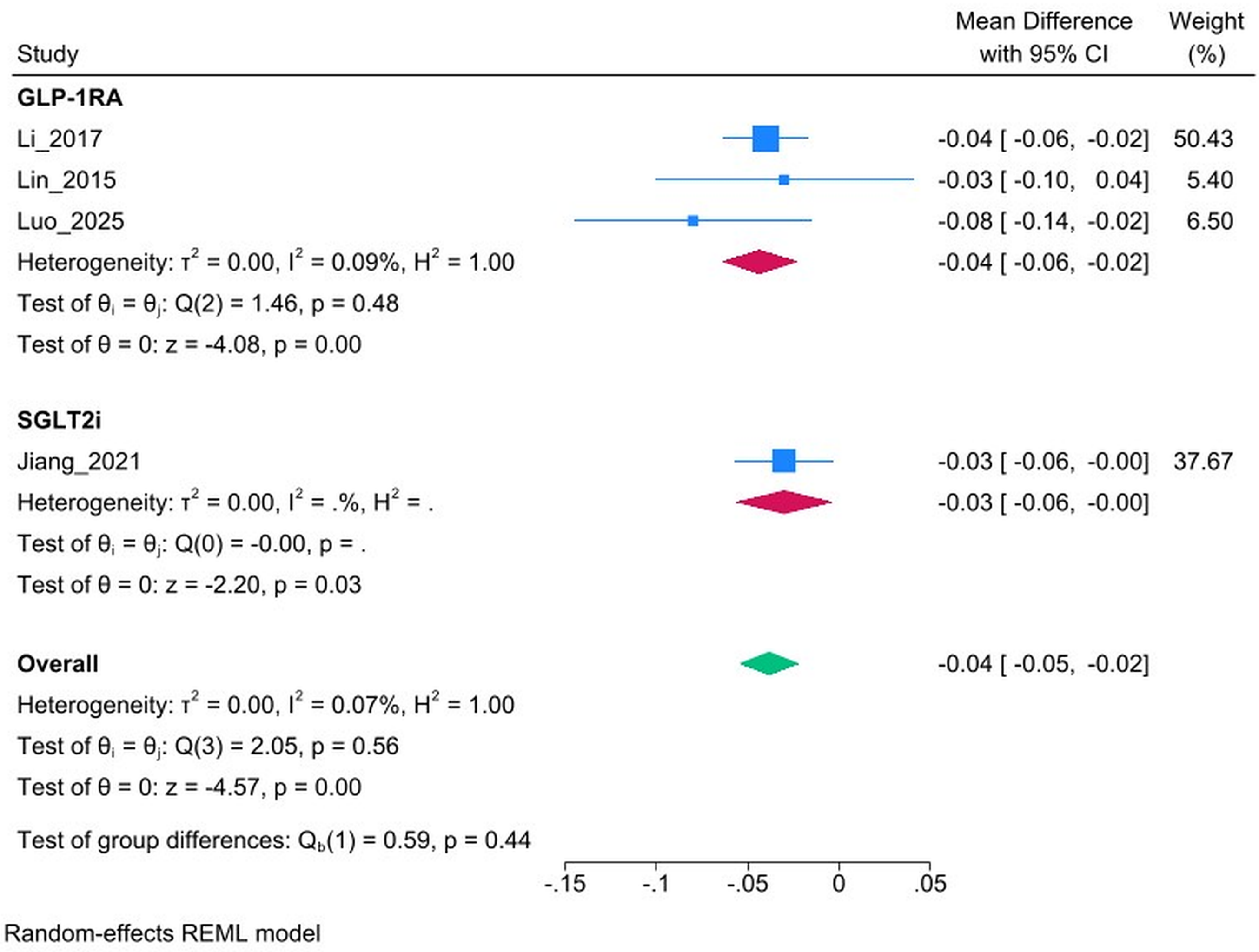

Supplementary Fig. 1 (Continued)

j

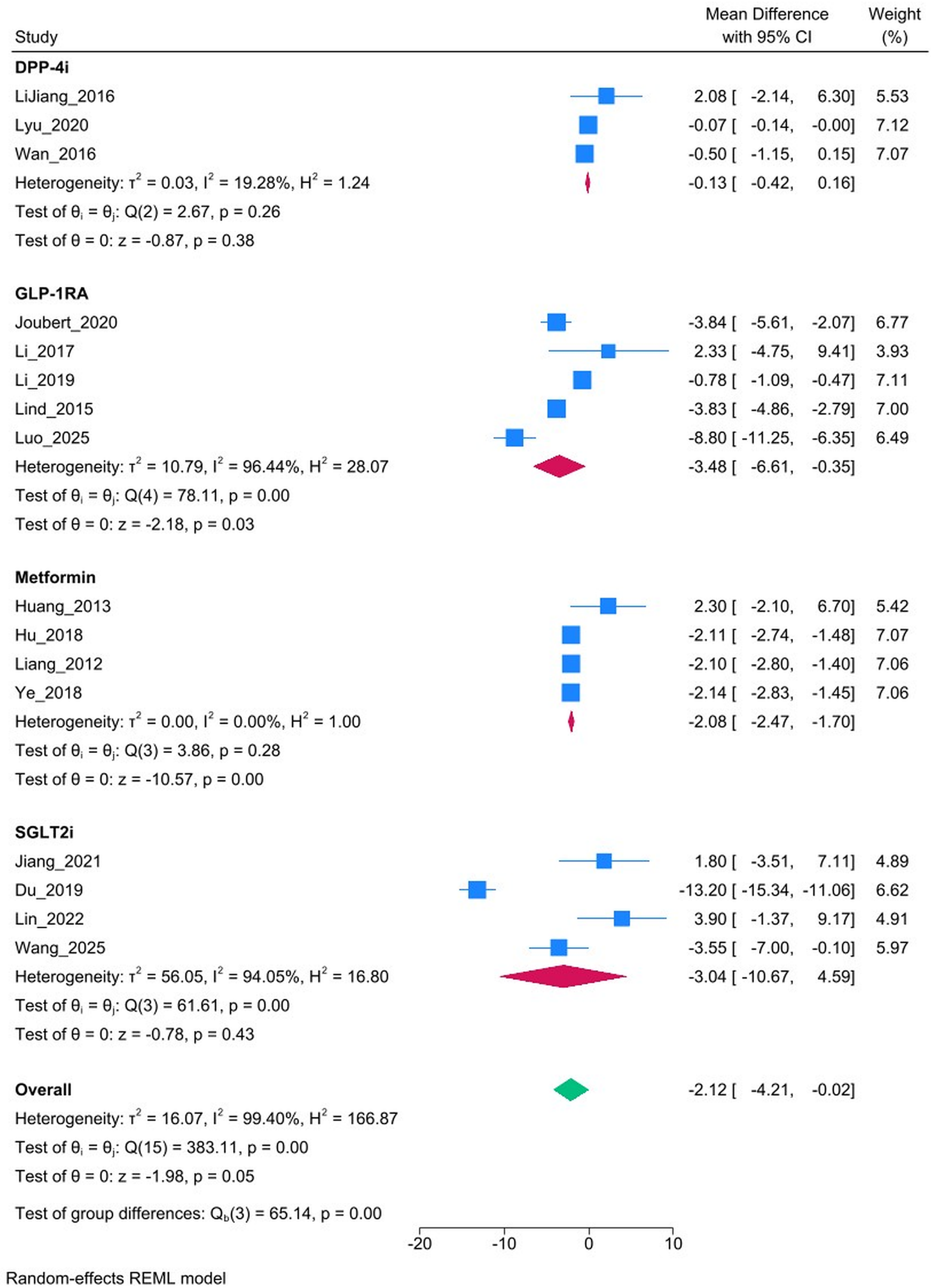

Supplementary Fig. 1 (Continued)

k

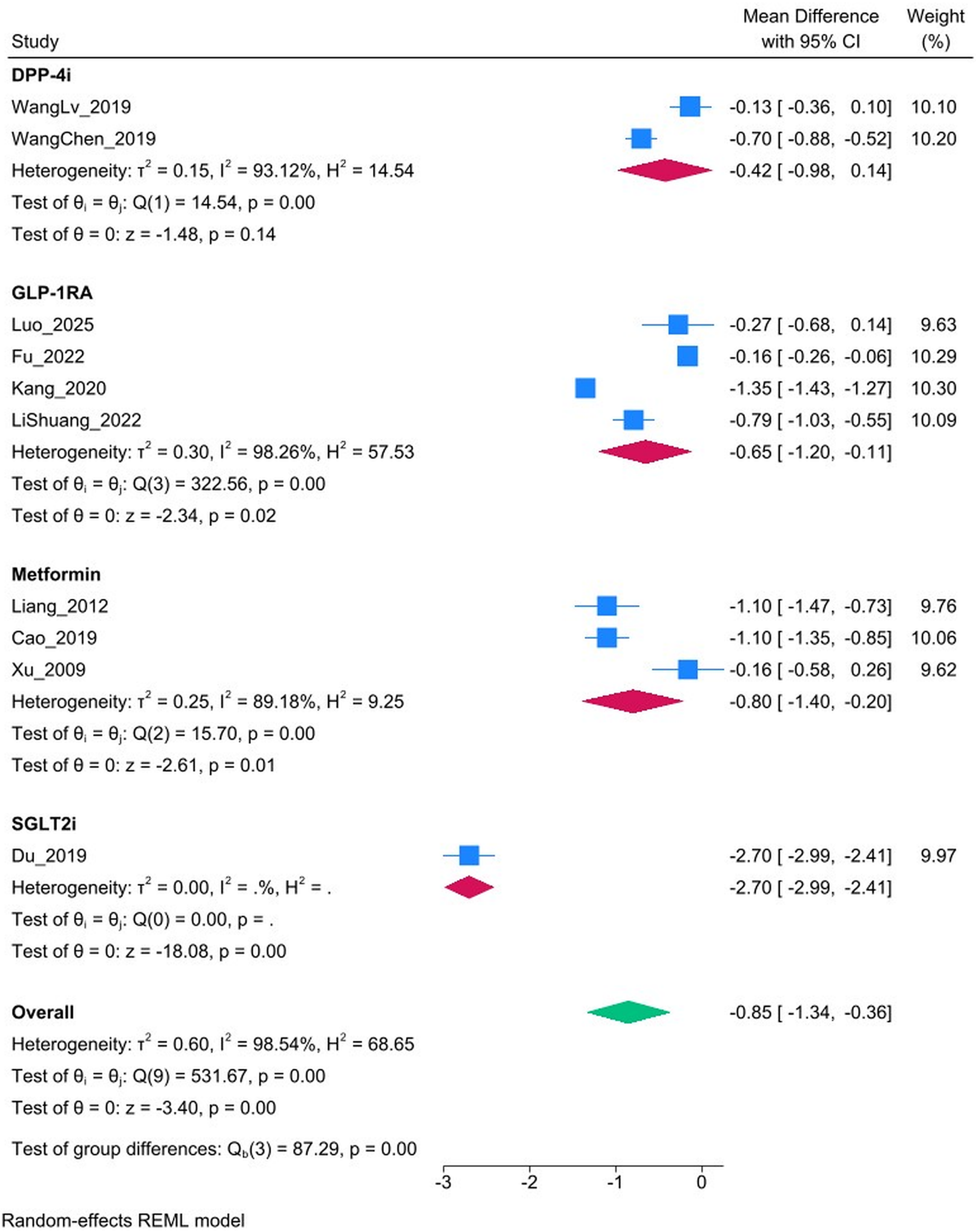

Supplementary Fig. 1 (Continued)

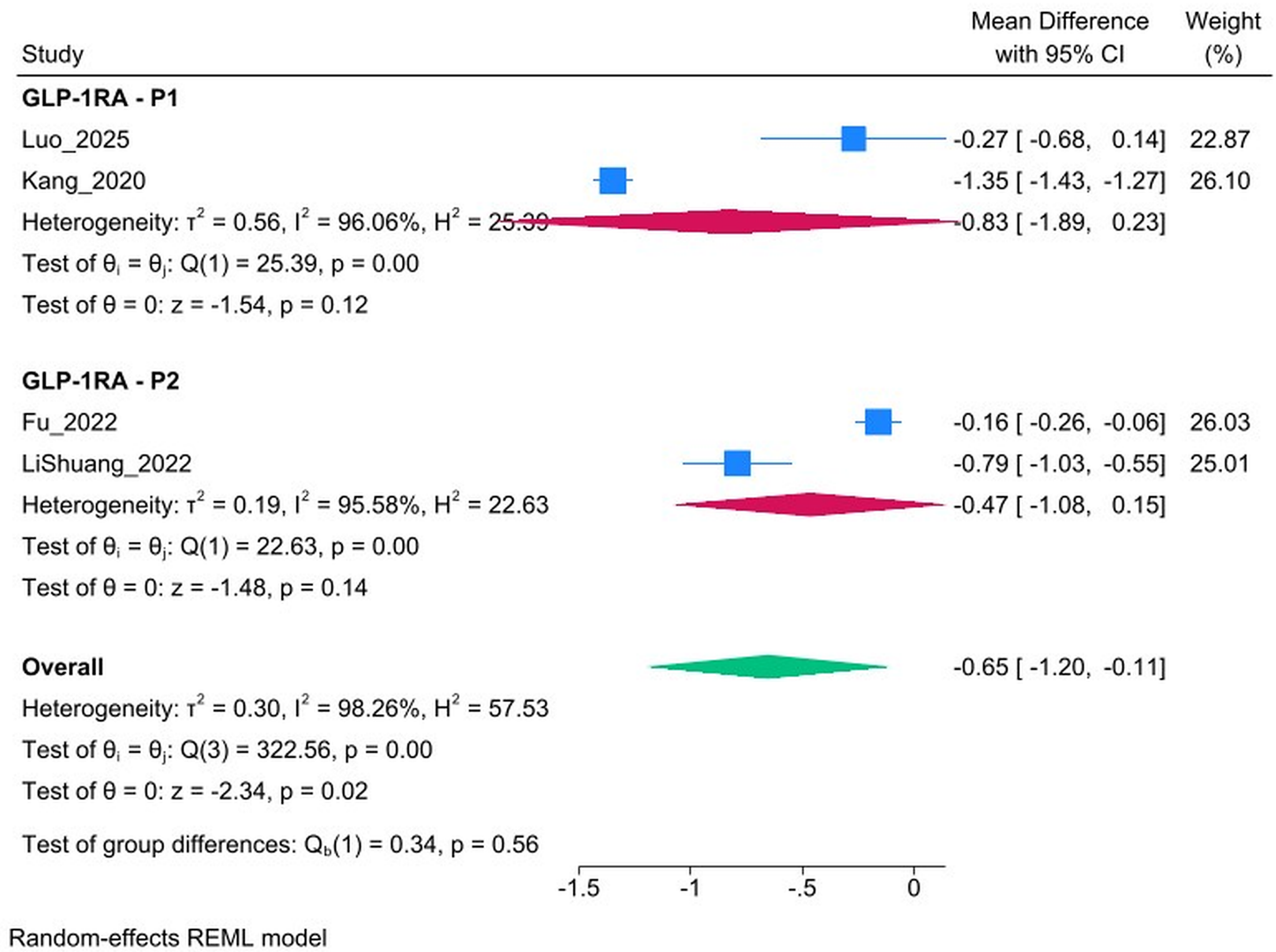

Supplementary Fig. 1 (Continued)

m

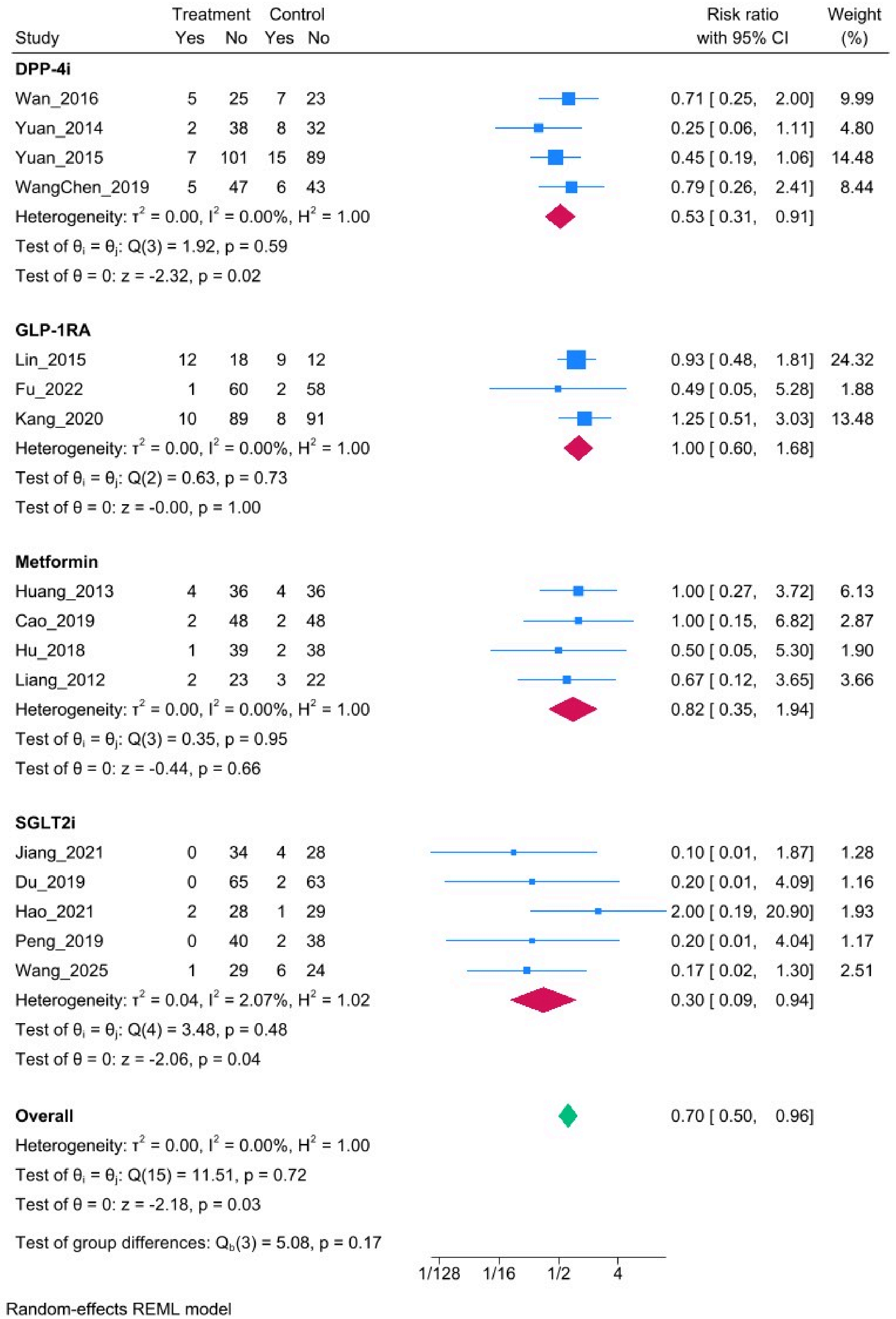

Supplementary Fig. 1 (Continued)

n

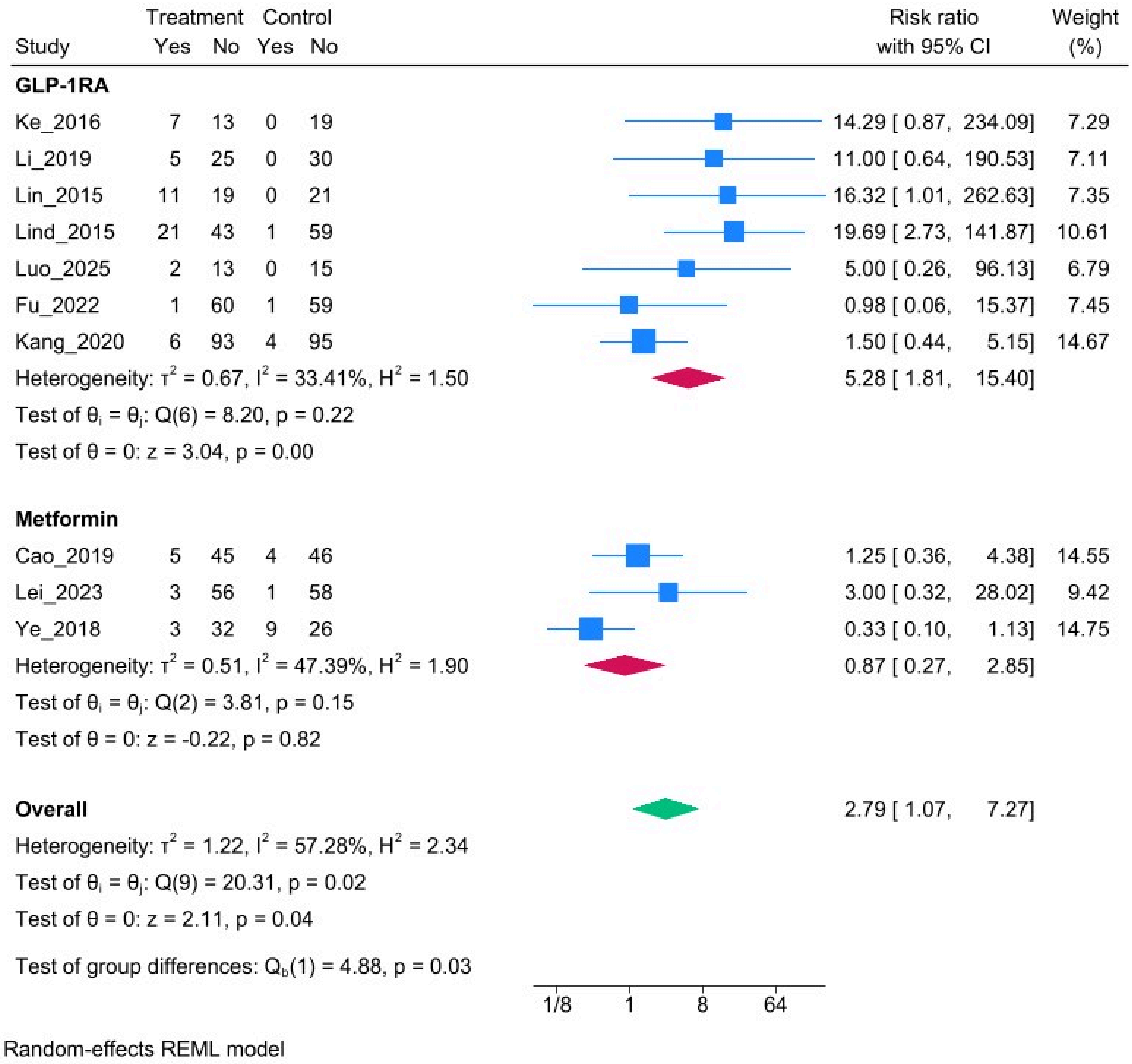

Supplementary Fig. 1 (Continued)
