# Supplementary material for: Effects of intensive insulin therapy combined with non-insulin antihyperglycemic agents on glycemic control, variability, and metabolic outcomes in patients with type 2 diabetes mellitus: a systematic review and meta-analysis
Source: Front Endocrinol (Lausanne). 2026 Oct 5;17:1948361. doi: 10.3389/fendo.2026.1948361 (PMC13677461; doi:10.3389/fendo.2026.1948361)
Supplement: Supplementary file 2 [file Image2.pdf]

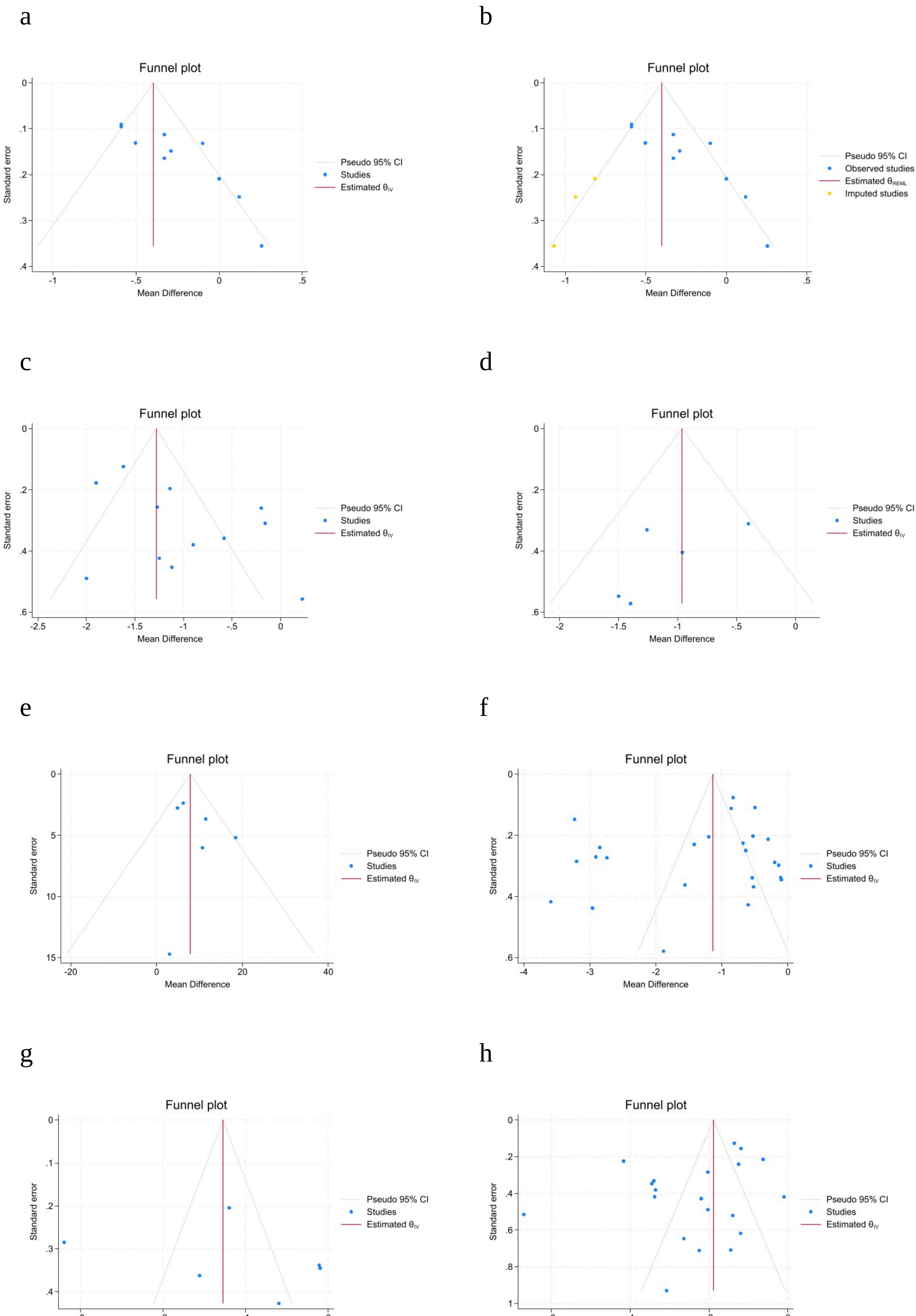

Supplementary Fig. 2. Funnel plots of (a) SDBG, (b) SDBG trim-and-fill, (c) MAGE, (d) LAGE, (e) TIR, (f) FPG, (g) FPG administration-method subgroup, (h) PPG-2h, (i) HbA1c, (j) HbA1c population subgroup, (k) MBG, (l) TDD, (m) Basal insulin dose, (n) Bolus insulin dose, (o) Body weight, (p) HOMA-IR, (q) HOMA-IR population subgroup, (r) hypoglycemia, (s) GI-AE.

i

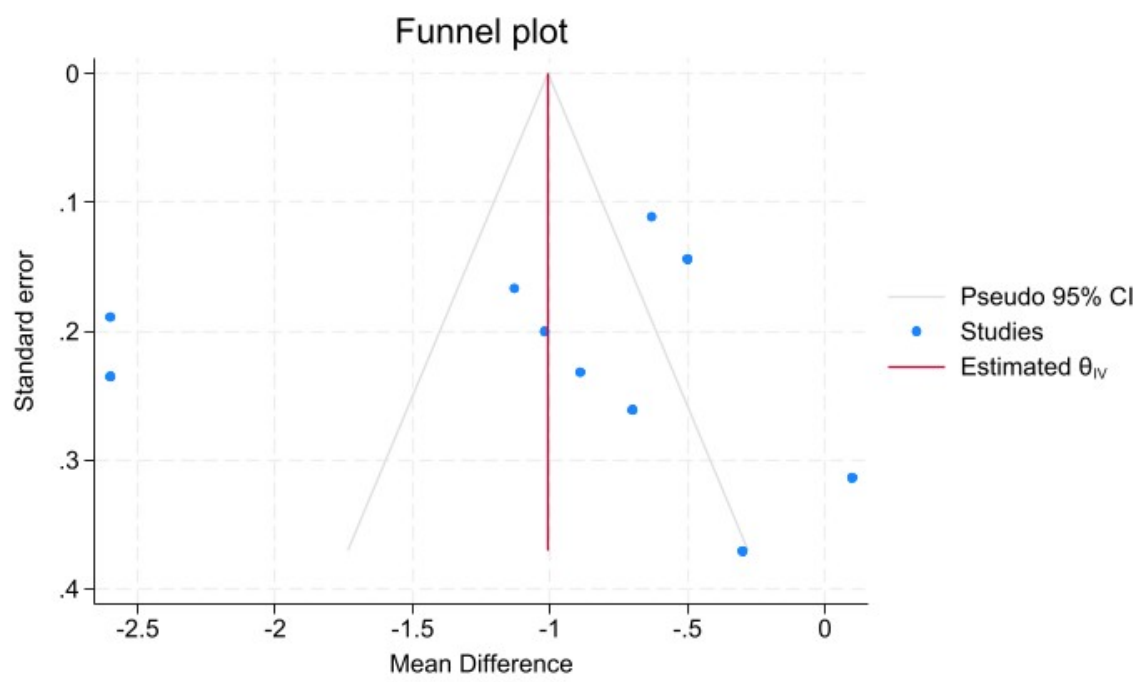

j

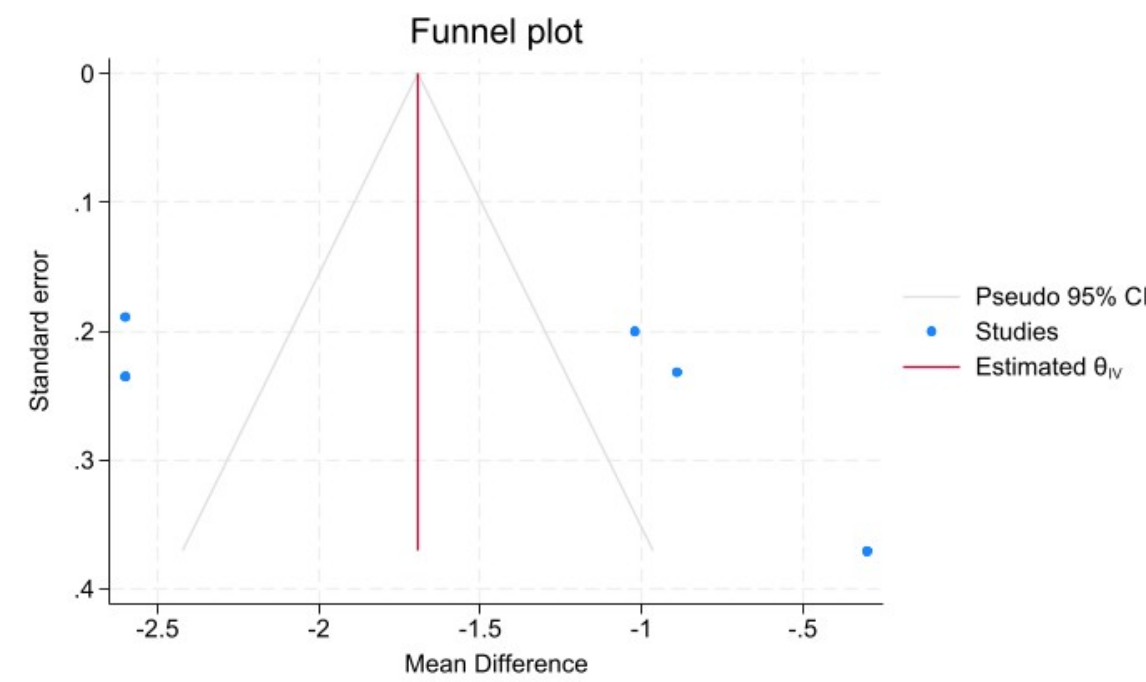

k

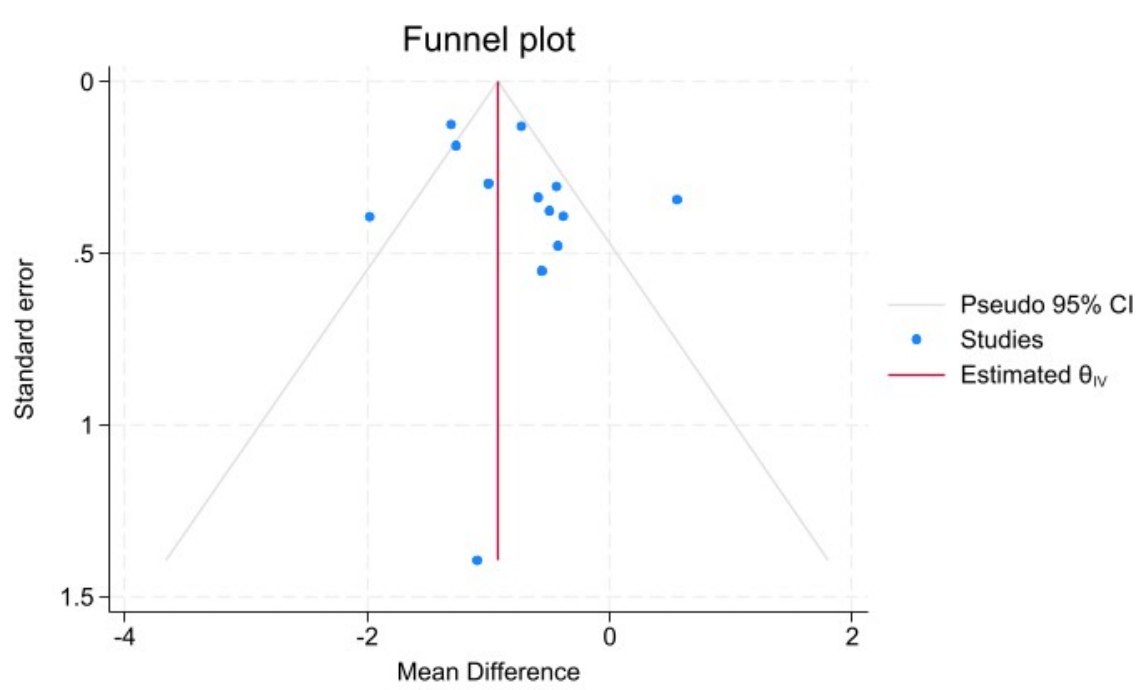

l

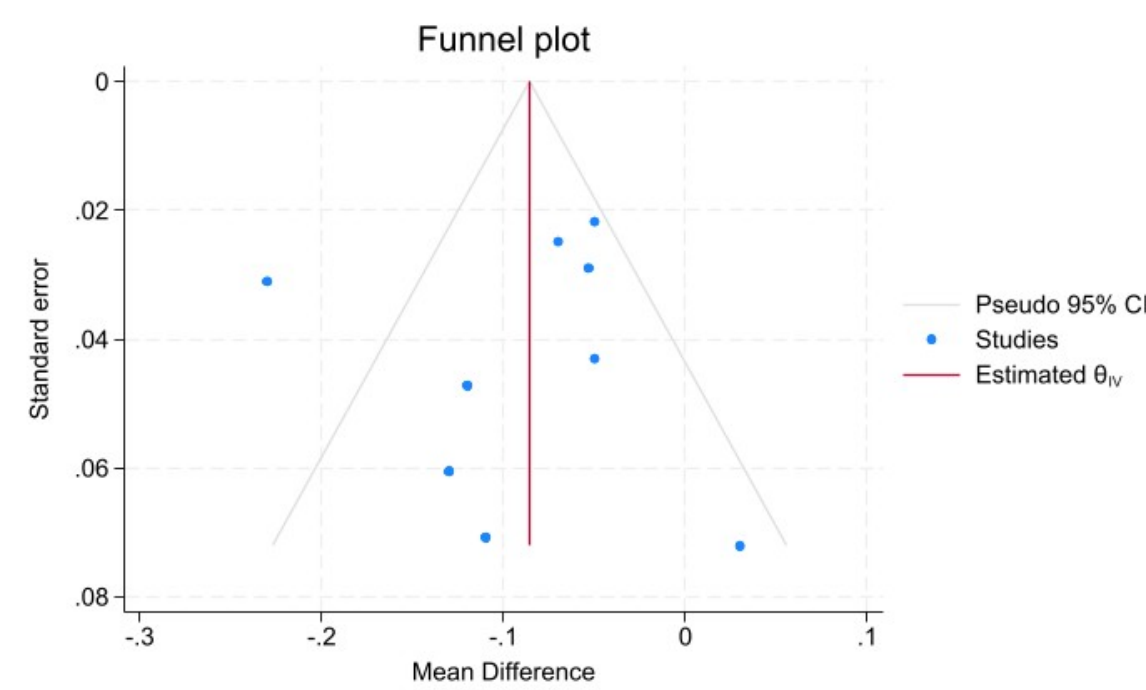

m

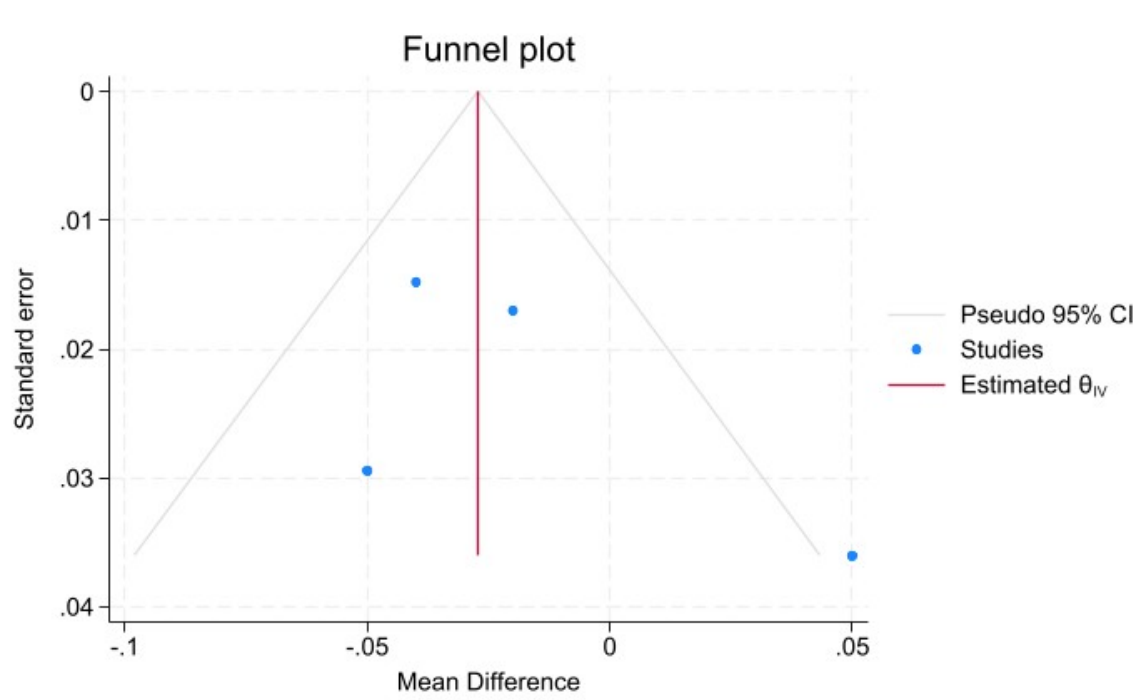

n

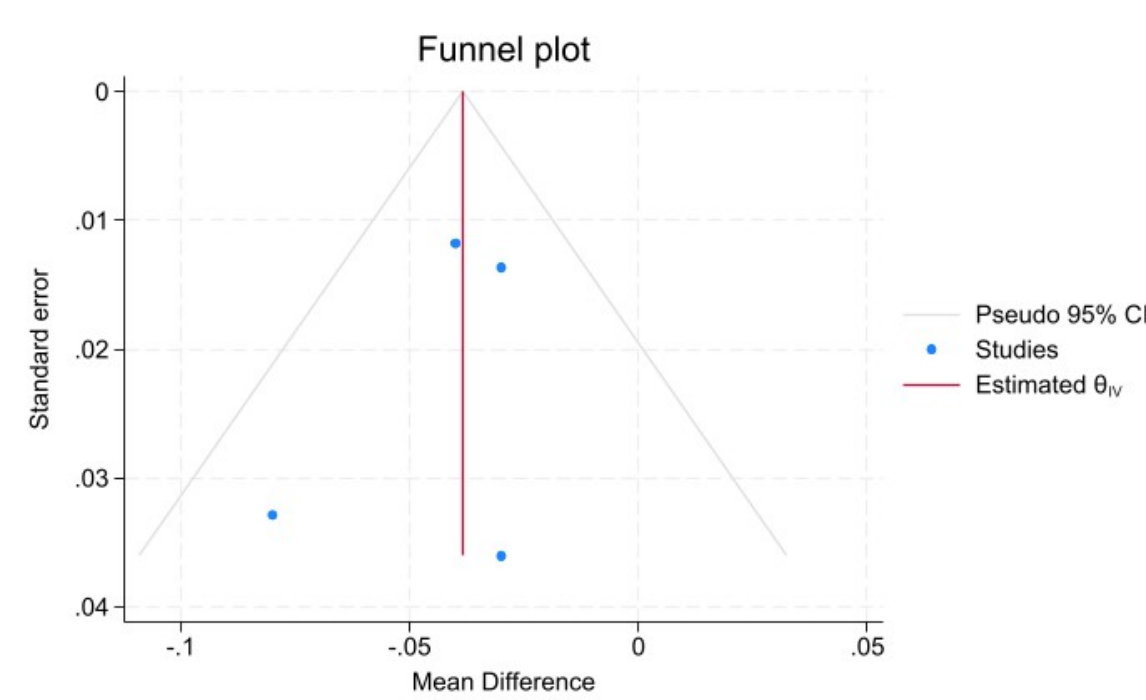

o

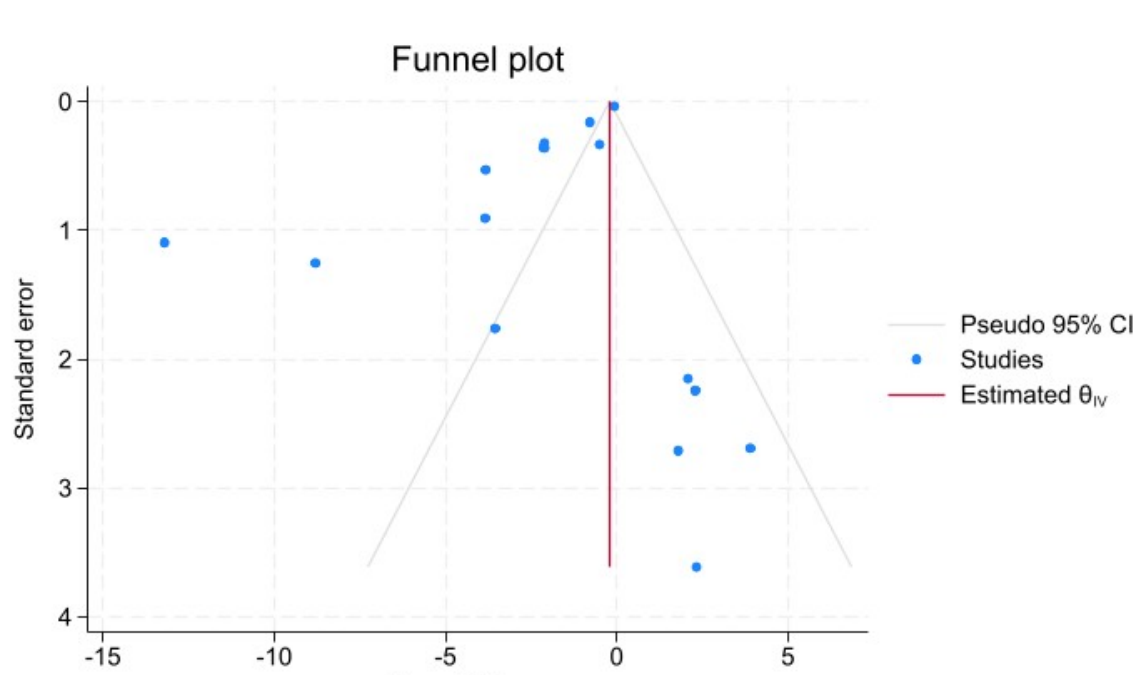

p

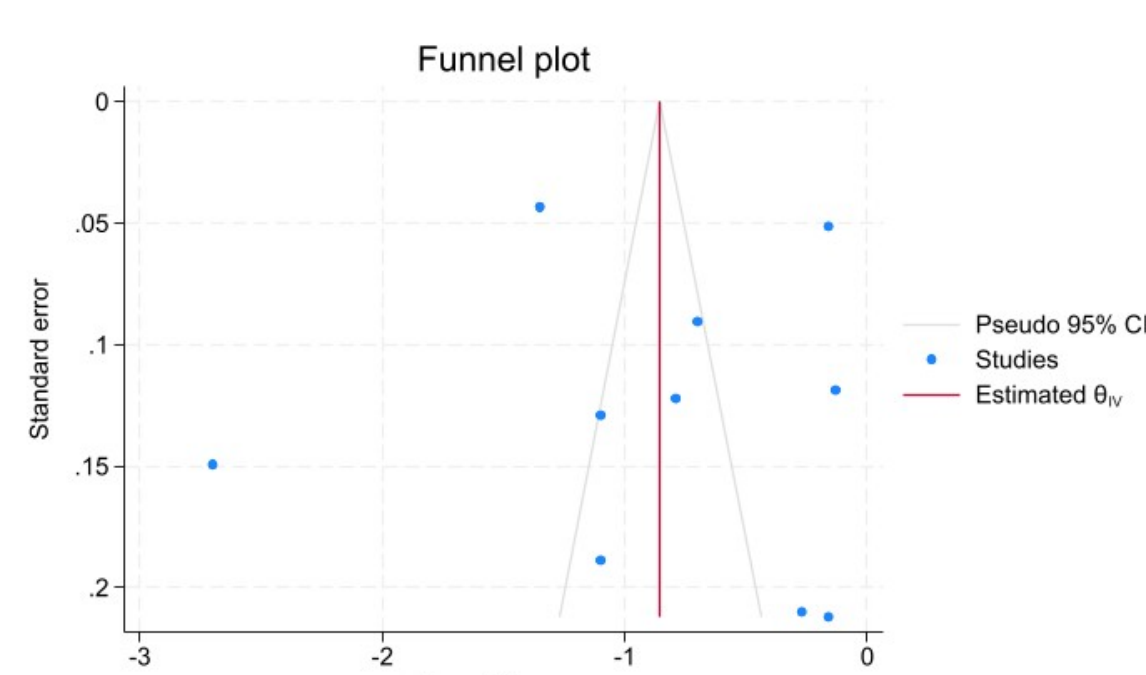

Supplementary Fig. 2 (Continued)

q

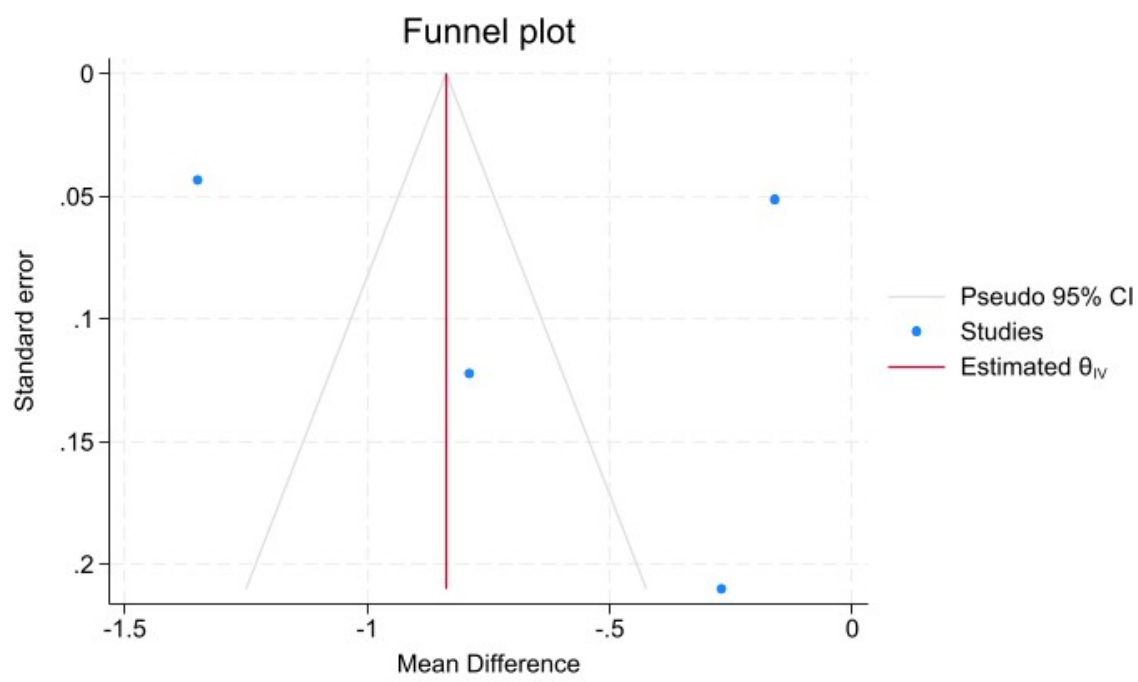

r

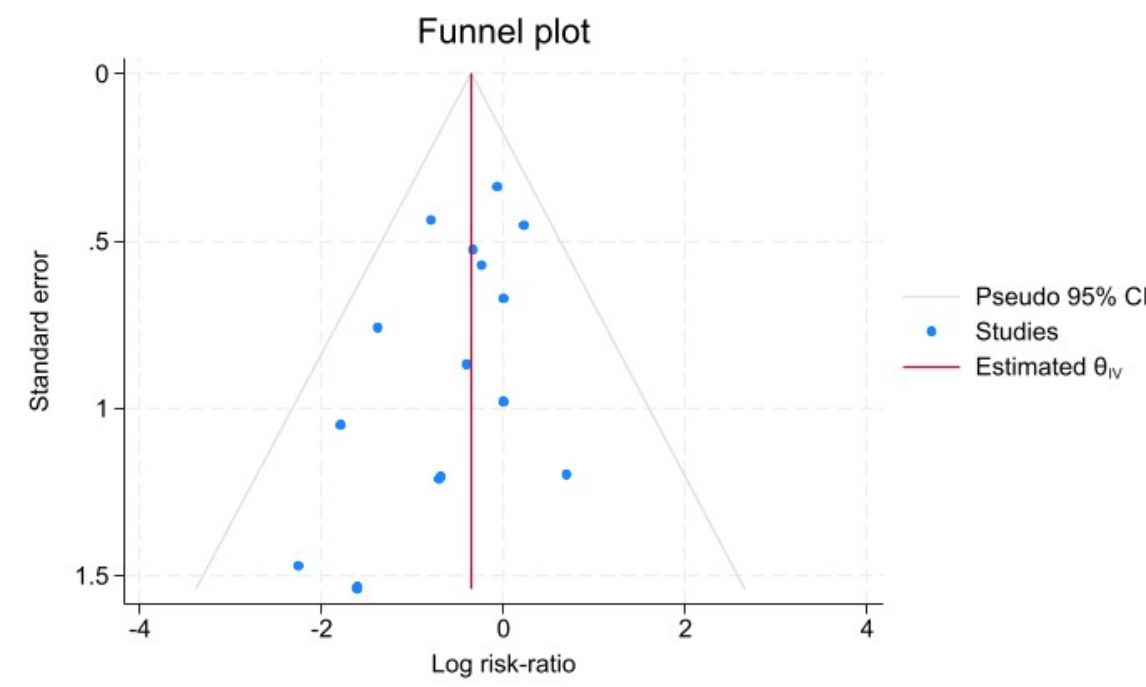

s

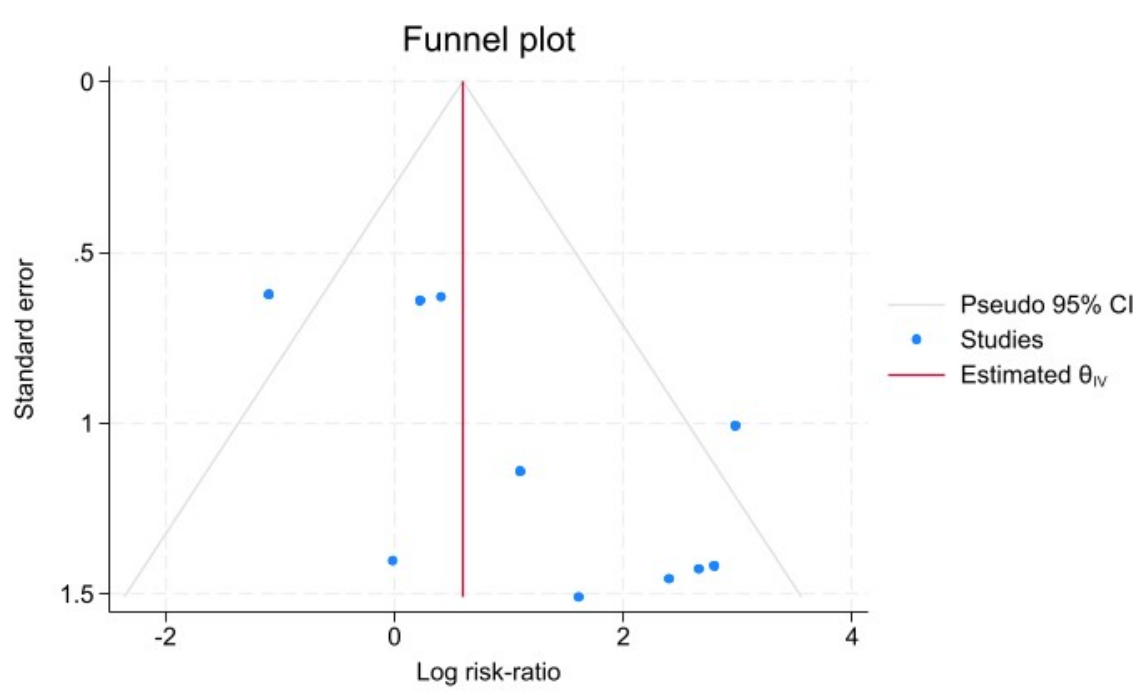

Supplementary Fig. 2 (Continued)
