# Supplementary material for: Effects of intensive insulin therapy combined with non-insulin antihyperglycemic agents on glycemic control, variability, and metabolic outcomes in patients with type 2 diabetes mellitus: a systematic review and meta-analysis
Source: Front Endocrinol (Lausanne). 2026 Oct 5;17:1948361. doi: 10.3389/fendo.2026.1948361 (PMC13677461; doi:10.3389/fendo.2026.1948361)
Supplement: Supplementary file 3 [file Table4.pdf]

Supplementary Table 4. GRADE assessment of certainty of evidence for drug-specific effects on FPG, 2-h PPG, and HbA1c

Drug-specific finding	Effect estimate (95% CI)	Studies	GRADE certainty assessment					Overall certainty	Key justification
			Risk of bias	Inconsistency	Indirectness	Imprecision	Publication bias		
**DPP-4i - FPG**	MD -0.44 mmol/L (95% CI -0.67 to -0.21)	5	Serious (-1)	Not serious	Not serious	Not serious	Not assessable	**Moderate**	Five trials contributed. Heterogeneity was absent (I^2=0%), and the pooled CI clearly excluded the null. Outcome-level RoB 2 was Some concerns in four assessed trials and Low in one.
**GLP-1RA - FPG**	MD -0.93 mmol/L (95% CI -1.32 to -0.54)	5	Serious (-1)	Serious (-1)	Not serious	Not serious	Not assessable	**Low**	The pooled effect favored lower FPG, but substantial heterogeneity was present (I^2=83.1%), with material variation in effect magnitude. Outcome-level RoB 2 assessments were predominantly Some concerns; Lind_2015 was rated Some concerns overall for FPG.
**Metformin - FPG**	MD -2.20 mmol/L (95% CI -3.04 to -1.36)	9	Serious (-1)	Serious (-1)	Not serious	Not serious	Not assessable	**Low**	The pooled CI excluded the null, but heterogeneity was extreme (I^2=97.4%) and study effects varied markedly in magnitude. Outcome-level RoB 2 was predominantly Some concerns, with Xu_2009 rated High.
**SGLT2i - FPG**	MD -1.14 mmol/L (95% CI -2.09 to -0.20)	6	Serious (-1)	Serious (-1)	Not serious	Not serious	Not assessable	**Low**	The pooled effect favored lower FPG, but heterogeneity was very high (I^2=93.1%) and individual effects ranged from near-null to large reductions. Outcome-level RoB 2 assessments were predominantly Some concerns; Du_2019 was rated Some concerns overall for FPG.
**DPP-4i - PPG-2h**	MD -1.37 mmol/L (95% CI -2.18 to -0.57)	5	Serious (-1)	Serious (-1)	Not serious	Not serious	Not assessable	**Low**	The pooled CI excluded the null, but heterogeneity was substantial (I^2=64.2%) and one study showed a much smaller, non-significant effect than the others. RoB 2 was Some concerns in four studies and Low in one.
**GLP-1RA - PPG-2h**	MD -1.30 mmol/L (95% CI -1.84 to -0.76)	4	Serious (-1)	Serious (-1)	Not serious	Not serious	Not assessable	**Low**	All four studies favored lower PPG-2h, but substantial heterogeneity remained (I^2=85.7%), indicating important variation in effect magnitude. Outcome-level RoB 2 was Some concerns across the contributing studies.
**Metformin - PPG-2h**	MD -2.92 mmol/L (95% CI -3.72 to -2.13)	8	Serious (-1)	Serious (-1)	Not serious	Not serious	Not assessable	**Low**	The pooled CI was clearly separated from the null, but heterogeneity was very high (I^2=91.9%) and the magnitude of reduction differed substantially among studies. Outcome-level RoB 2 was predominantly Some concerns.
**SGLT2i - PPG-2h**	MD -3.85 mmol/L (95% CI -6.67 to -1.03)	3	Serious (-1)	Serious (-1)	Not serious	Serious (-1)	Not assessable	**Very low**	Only three trials contributed. Heterogeneity was extreme (I^2=95.6%), and the pooled CI was wide (-6.67 to -1.03 mmol/L), leaving substantial uncertainty about effect magnitude. Outcome-level RoB 2 was Some concerns in the assessed contributing studies.
**GLP-1RA - HbA1c**	MD -0.73% (95% CI -1.01 to -0.45)	4	Serious (-1)	Not serious	Not serious	Not serious	Not assessable	**Moderate**	Although I^2 was 68.4%, all four studies favored lower HbA1c and their CIs showed substantial overlap; heterogeneity was judged to mainly reflect variation in effect magnitude. Outcome-level RoB 2 assessments included Low and Some concerns judgments; Lind_2015 was rated Some concerns overall for HbA1c.
**SGLT2i - HbA1c**	MD -1.50% (95% CI -2.42 to -0.58)	5	Serious (-1)	Serious (-1)	Not serious	Not serious	Not assessable	**Low**	The pooled CI excluded the null, but heterogeneity was extreme (I^2=95.1%) and study effects varied materially, including one estimate close to the null. Outcome-level RoB 2 was predominantly Some concerns.

GRADE interpretation: Serious (-1), downgraded by one level; Not serious, no downgrade; Not assessable, publication bias could not be evaluated. **Abbreviations:** CI, confidence interval; DPP-4i, dipeptidyl peptidase-4 inhibitor; FPG, fasting plasma glucose; GLP-1RA, glucagon-like peptide-1 receptor agonist; HbA1c, glycated hemoglobin; MD, mean difference; PPG-2h, 2-h postprandial glucose; SGLT2i, sodium-glucose cotransporter-2 inhibitor.
