# Supplementary material for: Effects of intensive insulin therapy combined with non-insulin antihyperglycemic agents on glycemic control, variability, and metabolic outcomes in patients with type 2 diabetes mellitus: a systematic review and meta-analysis
Source: Front Endocrinol (Lausanne). 2026 Oct 5;17:1948361. doi: 10.3389/fendo.2026.1948361 (PMC13677461; doi:10.3389/fendo.2026.1948361)
Supplement: Supplementary file 4 [file SupplementaryFile2.docx]

Supplementary Material 2. Detailed GRADE assessment and justifications

| **Drug-specific finding** | **Effect estimate (95% CI)** | **Studies** | **Risk of bias** | **Inconsistency** | **Indirectness** | **Imprecision** | **Publication bias** | **Overall certainty** | **Key justification** |
| --- | --- | --- | --- | --- | --- | --- | --- | --- | --- |
| **DPP-4i – MAGE** | MD −1.39 mmol/L (95% CI −1.77 to −1.01) | 4 | Serious ↓1 | Not serious | Not serious | Not serious | Not assessable | **Moderate** | Although I² was 59.2%, all four studies favored lower MAGE and their CIs showed substantial overlap. The heterogeneity was therefore judged to reflect variation in effect magnitude rather than clinically important inconsistency. RoB 2 was “Some concerns” in three studies and Low in one. |
| **GLP-1RA – MAGE** | MD −0.61 mmol/L (95% CI −1.19 to −0.02) | 3 | Serious ↓1 | Serious ↓1 | Not serious | Serious ↓1 | Not assessable | **Very low** | Study effects varied materially and the pooled CI approached the null. |
| **DPP-4i – SDBG** | MD −0.46 mmol/L (95% CI −0.75 to −0.17) | 2 | Serious ↓1 | Serious ↓1 | Not serious | Serious ↓1 | Not assessable | **Very low** | Only two trials contributed; I² was 65.4% and the study-specific effect sizes differed. The small evidence base also limits precision despite the pooled CI not crossing the null. |
| **SGLT2i – SDBG** | MD −0.28 mmol/L (95% CI −0.44 to −0.11) | 3 | Serious ↓1 | Not serious | Not serious | Not serious | Not assessable | **Moderate** | The three-study subgroup showed consistent effects with I²=0%. RoB 2 was predominantly “Some concerns,” but no additional downgrade was supported for inconsistency or imprecision. |
| **DPP-4i – LAGE** | MD −1.20 mmol/L (95% CI −1.66 to −0.75) | 3 | Serious ↓1 | Not serious | Not serious | Not serious | Not assessable | **Moderate** | The three studies were directionally consistent with I²=0% and the pooled CI was clearly separated from the null. The main limitation was outcome-level RoB 2. |
| **SGLT2i – TIR** | MD +8.23 percentage points (95% CI 4.13 to 12.32) | 3 | Serious ↓1 | Not serious | Not serious | Not serious | Not assessable | **Moderate** | Outcome-level RoB 2 was predominantly “Some concerns,” mainly from incomplete allocation-concealment reporting. Heterogeneity was low (I²=11.4%), the direction of effect was consistent, and the CI did not cross the null. |
| **GLP-1RA, longer duration – Body weight** | MD −3.63 kg (95% CI −4.94 to −2.32) | 3 | Serious ↓1 | Not serious | Serious ↓1 (post hoc subgroup) | Not serious | Not assessable | Low | The contributing trials directly evaluated GLP-1RA added to IIT, and the pooled CI was clearly separated from the null. However, the long- versus short-duration body-weight subgroup was defined post hoc. Because the subgroup-specific inference depends on a post hoc study-level duration categorization, certainty was downgraded one level for indirectness/credibility. Outcome-level RoB 2 for Lind_2015–Body weight was present and judged overall “Some concerns”; together with the class-level RoB downgrade, the final certainty is Low. |
| **DPP-4i – Total insulin dose** | MD −0.05 (95% CI −0.10 to −0.01; pooled insulin-dose scale) | 2 | Serious ↓1 | Not serious | Not serious | Serious ↓1 | Not assessable | **Low** | Only two studies contributed and the CI lay close to the null, so imprecision was downgraded. |
| **GLP-1RA – Total insulin dose** | MD −0.10 (95% CI −0.19 to −0.02; pooled insulin-dose scale) | 5 | Serious ↓1 | Serious ↓1 | Not serious | Not serious | Not assessable | **Low** | Substantial heterogeneity was present (I²=81.1%). Outcome-level RoB 2 was Low in one study and “Some concerns” in the others, largely because of allocation concealment and treatment-aware insulin titration. |
| **DPP-4i – Hypoglycemia** | RR 0.53 (95% CI 0.31 to 0.90) | 4 | Serious ↓1 | Not serious | Not serious | Serious ↓1 | Not assessable | **Low** | Statistical heterogeneity was absent (I²=0%), but event counts were limited and hypoglycaemia ascertainment was not uniformly device-defined; several trials had D4 “Some concerns.” |
| **SGLT2i – Hypoglycemia** | RR 0.30 (95% CI 0.09 to 0.94) | 5 | Serious ↓1 | Not serious | Not serious | Very serious ↓2 | Not assessable | **Very low** | The CI was very wide and events were sparse despite low heterogeneity (I²=2.1%). Outcome-level RoB 2 was “Some concerns” across contributing studies. This result must not be presented as a firm class benefit. |
| **GLP-1RA – GI-AEs** | RR 5.28 (95% CI 1.81 to 15.40) | 7 | Serious ↓1 | Not serious | Not serious | Serious ↓1 | Not assessable | **Low** | Heterogeneity was modest (I²=33.4%), but the CI was wide, leaving substantial uncertainty about effect magnitude. Outcome-level RoB 2 assessments were available for the contributing trials, including Lind_2015–GI-AEs (overall “Some concerns”; D1 and D5 Some concerns, D2–D4 Low). GI-AEs are partly subjective outcomes, and the evidence base therefore retained a one-level risk-of-bias downgrade plus a one-level imprecision downgrade. |

**Abbreviations:** CI, confidence interval; DPP-4i, dipeptidyl peptidase-4 inhibitor; GI-AEs, gastrointestinal adverse events; GLP-1RA, glucagon-like peptide-1 receptor agonist; LAGE, largest amplitude of glycaemic excursions; MAGE, mean amplitude of glycaemic excursions; MD, mean difference; RR, risk ratio; SDBG, standard deviation of blood glucose; SGLT2i, sodium-glucose cotransporter-2 inhibitor; TIR, time in range.
