# Supplementary material for: Effects of intensive insulin therapy combined with non-insulin antihyperglycemic agents on glycemic control, variability, and metabolic outcomes in patients with type 2 diabetes mellitus: a systematic review and meta-analysis
Source: Front Endocrinol (Lausanne). 2026 Oct 5;17:1948361. doi: 10.3389/fendo.2026.1948361 (PMC13677461; doi:10.3389/fendo.2026.1948361)
Supplement: Supplementary file 5 [file SupplementaryFile5.docx]

Supplementary Material 5

**Full electronic search strategies for the updated literature search**

The literature search was updated to 7 November 2025. The complete database- and registry-specific search strategies are presented below, together with the corresponding search fields, date restrictions, and numbers of records retrieved.

# 1. Cochrane Central Register of Controlled Trials (CENTRAL), Cochrane Library

**Coverage:** Database inception to 7 November 2025

**Language restriction:** None

**RCT identification:** No additional methodological RCT text filter was applied. Randomized allocation was verified during eligibility screening.

**Date restriction:** Cochrane Library publication date, custom range from database inception to 7 November 2025

**Records retrieved:** n = 370

## Exact executed search history

| **ID** | **Search** | **Hits** |
| --- | --- | --- |
| #9 | MeSH descriptor: [Diabetes Mellitus, Type 2] explode all trees | 28,379 |
| #10 | ("type 2 diabetes" OR "type 2 diabetes mellitus" OR "diabetes mellitus, type 2" OR T2DM):ti,ab,kw | 61,213 |
| #11 | #9 OR #10 | 61,214 |
| #12 | ("intensive insulin therapy" OR "intensive insulin treatment" OR "intensive insulin" OR "short-term intensive insulin therapy" OR "short term intensive insulin therapy" OR IIT OR SIIT OR "continuous subcutaneous insulin infusion" OR CSII OR "insulin pump" OR "insulin pumps" OR "multiple daily insulin injections" OR "multiple daily injections" OR MDI OR "basal-bolus insulin" OR "basal bolus insulin"):ti,ab,kw | 7,354 |
| #13 | MeSH descriptor: [Metformin] explode all trees | 5,735 |
| #14 | (metformin OR "metformin hydrochloride"):ti,ab,kw | 15,461 |
| #15 | MeSH descriptor: [Dipeptidyl-Peptidase IV Inhibitors] explode all trees | 850 |
| #16 | ("DPP-4 inhibitor" OR "DPP-4 inhibitors" OR "DPP4 inhibitor" OR "DPP4 inhibitors" OR "dipeptidyl peptidase-4 inhibitor" OR "dipeptidyl peptidase-4 inhibitors" OR sitagliptin OR saxagliptin OR vildagliptin OR linagliptin OR alogliptin OR teneligliptin OR gemigliptin OR anagliptin OR evogliptin OR trelagliptin OR omarigliptin):ti,ab,kw | 5,703 |
| #17 | MeSH descriptor: [Sodium-Glucose Transporter 2 Inhibitors] explode all trees | 1,410 |
| #18 | ("SGLT2 inhibitor" OR "SGLT2 inhibitors" OR "SGLT-2 inhibitor" OR "SGLT-2 inhibitors" OR "sodium-glucose cotransporter 2 inhibitor" OR "sodium-glucose cotransporter 2 inhibitors" OR dapagliflozin OR empagliflozin OR canagliflozin OR ertugliflozin OR ipragliflozin OR luseogliflozin OR tofogliflozin OR remogliflozin OR bexagliflozin):ti,ab,kw | 7,824 |
| #19 | MeSH descriptor: [Glucagon-Like Peptide-1 Receptor Agonists] explode all trees | 449 |
| #20 | ("GLP-1 receptor agonist" OR "GLP-1 receptor agonists" OR "GLP1 receptor agonist" OR "GLP1 receptor agonists" OR "glucagon-like peptide-1 receptor agonist" OR "glucagon-like peptide-1 receptor agonists" OR exenatide OR liraglutide OR lixisenatide OR dulaglutide OR semaglutide OR albiglutide OR beinaglutide OR efpeglenatide OR taspoglutide):ti,ab,kw | 7,878 |
| #21 | #13 OR #14 OR #15 OR #16 OR #17 OR #18 OR #19 OR #20 | 29,362 |
| #22 | #11 AND #12 AND #21 | 370 |

**Limit applied to #22:** Cochrane Library publication date: database inception to 7 November 2025

# 2. Web of Science Core Collection

**Coverage:** Database inception to 7 November 2025

**Records retrieved:** n = 418

```
TS=(
  "type 2 diabetes"
  OR "type 2 diabetes mellitus"
  OR "diabetes mellitus, type 2"
  OR T2DM
)
AND
TS=(
  "intensive insulin therapy"
  OR "intensive insulin treatment"
  OR "intensive insulin"
  OR "short-term intensive insulin therapy"
  OR "short term intensive insulin therapy"
  OR IIT
  OR SIIT
  OR "continuous subcutaneous insulin infusion"
  OR CSII
  OR "insulin pump"
  OR "insulin pumps"
  OR "multiple daily insulin injections"
  OR "multiple daily injections"
  OR MDI
  OR "basal-bolus insulin"
  OR "basal bolus insulin"
)
AND
TS=(
  metformin
  OR "metformin hydrochloride"
  OR "DPP-4 inhibitor*"
  OR "DPP4 inhibitor*"
  OR "dipeptidyl peptidase-4 inhibitor*"
  OR sitagliptin
  OR saxagliptin
  OR vildagliptin
  OR linagliptin
  OR alogliptin
  OR teneligliptin
  OR gemigliptin
  OR anagliptin
  OR evogliptin
  OR trelagliptin
  OR omarigliptin
  OR "SGLT2 inhibitor*"
  OR "SGLT-2 inhibitor*"
  OR "sodium-glucose cotransporter 2 inhibitor*"
  OR dapagliflozin
  OR empagliflozin
  OR canagliflozin
  OR ertugliflozin
  OR ipragliflozin
  OR luseogliflozin
  OR tofogliflozin
  OR remogliflozin
  OR bexagliflozin
  OR "GLP-1 receptor agonist*"
  OR "GLP1 receptor agonist*"
  OR "glucagon-like peptide-1 receptor agonist*"
  OR exenatide
  OR liraglutide
  OR lixisenatide
  OR dulaglutide
  OR semaglutide
  OR albiglutide
  OR beinaglutide
  OR efpeglenatide
  OR taspoglutide
)
AND
DOP=1900-01-01/2025-11-07
```

# 3. PubMed

**Coverage:** Database inception to 7 November 2025

**Records retrieved:** n = 417

```
(
  "Diabetes Mellitus, Type 2"[Mesh]
  OR "type 2 diabetes"[tiab]
  OR "type 2 diabetes mellitus"[tiab]
  OR "diabetes mellitus, type 2"[tiab]
  OR T2DM[tiab]
)
AND
(
  "intensive insulin therapy"[tiab]
  OR "intensive insulin treatment"[tiab]
  OR "intensive insulin"[tiab]
  OR "short-term intensive insulin therapy"[tiab]
  OR "short term intensive insulin therapy"[tiab]
  OR "short-term intensive insulin treatment"[tiab]
  OR IIT[tiab]
  OR SIIT[tiab]
  OR "continuous subcutaneous insulin infusion"[tiab]
  OR CSII[tiab]
  OR "insulin pump"[tiab]
  OR "insulin pumps"[tiab]
  OR "multiple daily insulin injections"[tiab]
  OR "multiple daily injections"[tiab]
  OR MDI[tiab]
  OR "basal-bolus insulin"[tiab]
  OR "basal bolus insulin"[tiab]
  OR "basal-bolus therapy"[tiab]
)
AND
(
  "Metformin"[Mesh]
  OR metformin[tiab]
  OR "metformin hydrochloride"[tiab]
  OR "Dipeptidyl-Peptidase IV Inhibitors"[Mesh]
  OR "DPP-4 inhibitor"[tiab]
  OR "DPP-4 inhibitors"[tiab]
  OR "DPP4 inhibitor"[tiab]
  OR "DPP4 inhibitors"[tiab]
  OR "dipeptidyl peptidase-4 inhibitor"[tiab]
  OR "dipeptidyl peptidase-4 inhibitors"[tiab]
  OR sitagliptin[tiab]
  OR saxagliptin[tiab]
  OR vildagliptin[tiab]
  OR linagliptin[tiab]
  OR alogliptin[tiab]
  OR teneligliptin[tiab]
  OR gemigliptin[tiab]
  OR anagliptin[tiab]
  OR evogliptin[tiab]
  OR trelagliptin[tiab]
  OR omarigliptin[tiab]
  OR "Sodium-Glucose Transporter 2 Inhibitors"[Mesh]
  OR "SGLT2 inhibitor"[tiab]
  OR "SGLT2 inhibitors"[tiab]
  OR "SGLT-2 inhibitor"[tiab]
  OR "SGLT-2 inhibitors"[tiab]
  OR "sodium-glucose cotransporter 2 inhibitor"[tiab]
  OR "sodium-glucose cotransporter 2 inhibitors"[tiab]
  OR dapagliflozin[tiab]
  OR empagliflozin[tiab]
  OR canagliflozin[tiab]
  OR ertugliflozin[tiab]
  OR ipragliflozin[tiab]
  OR luseogliflozin[tiab]
  OR tofogliflozin[tiab]
  OR remogliflozin[tiab]
  OR bexagliflozin[tiab]
  OR "Glucagon-Like Peptide-1 Receptor Agonists"[Mesh]
  OR "GLP-1 receptor agonist"[tiab]
  OR "GLP-1 receptor agonists"[tiab]
  OR "GLP1 receptor agonist"[tiab]
  OR "GLP1 receptor agonists"[tiab]
  OR "glucagon-like peptide-1 receptor agonist"[tiab]
  OR "glucagon-like peptide-1 receptor agonists"[tiab]
  OR exenatide[tiab]
  OR liraglutide[tiab]
  OR lixisenatide[tiab]
  OR dulaglutide[tiab]
  OR semaglutide[tiab]
  OR albiglutide[tiab]
  OR beinaglutide[tiab]
  OR efpeglenatide[tiab]
  OR taspoglutide[tiab]
)
AND
(
  "1900/01/01"[Date - Publication] :
  "2025/11/07"[Date - Publication]
)
```

# 4. Embase

**Records retrieved:** n = 1,422

```
(
  'type 2 diabetes mellitus'/exp
  OR 'type 2 diabetes':ti,ab,kw
  OR 'type 2 diabetes mellitus':ti,ab,kw
  OR 'diabetes mellitus, type 2':ti,ab,kw
  OR t2dm:ti,ab,kw
)
AND
(
  'intensive insulin therapy':ti,ab,kw
  OR 'intensive insulin treatment':ti,ab,kw
  OR 'intensive insulin':ti,ab,kw
  OR 'short-term intensive insulin therapy':ti,ab,kw
  OR 'short term intensive insulin therapy':ti,ab,kw
  OR iit:ti,ab,kw
  OR siit:ti,ab,kw
  OR 'continuous subcutaneous insulin infusion':ti,ab,kw
  OR csii:ti,ab,kw
  OR 'insulin pump':ti,ab,kw
  OR 'insulin pumps':ti,ab,kw
  OR 'multiple daily insulin injections':ti,ab,kw
  OR 'multiple daily injections':ti,ab,kw
  OR mdi:ti,ab,kw
  OR 'basal-bolus insulin':ti,ab,kw
  OR 'basal bolus insulin':ti,ab,kw
)
AND
(
  'metformin'/exp
  OR metformin:ti,ab,kw
  OR 'metformin hydrochloride':ti,ab,kw
  OR 'dipeptidyl peptidase 4 inhibitor'/exp
  OR 'dpp-4 inhibitor':ti,ab,kw
  OR 'dpp-4 inhibitors':ti,ab,kw
  OR 'dipeptidyl peptidase-4 inhibitor':ti,ab,kw
  OR 'dipeptidyl peptidase-4 inhibitors':ti,ab,kw
  OR sitagliptin:ti,ab,kw
  OR saxagliptin:ti,ab,kw
  OR vildagliptin:ti,ab,kw
  OR linagliptin:ti,ab,kw
  OR alogliptin:ti,ab,kw
  OR teneligliptin:ti,ab,kw
  OR gemigliptin:ti,ab,kw
  OR anagliptin:ti,ab,kw
  OR evogliptin:ti,ab,kw
  OR trelagliptin:ti,ab,kw
  OR omarigliptin:ti,ab,kw
  OR 'sodium glucose cotransporter 2 inhibitor'/exp
  OR 'sglt2 inhibitor':ti,ab,kw
  OR 'sglt2 inhibitors':ti,ab,kw
  OR 'sglt-2 inhibitor':ti,ab,kw
  OR 'sglt-2 inhibitors':ti,ab,kw
  OR 'sodium-glucose cotransporter 2 inhibitor':ti,ab,kw
  OR 'sodium-glucose cotransporter 2 inhibitors':ti,ab,kw
  OR dapagliflozin:ti,ab,kw
  OR empagliflozin:ti,ab,kw
  OR canagliflozin:ti,ab,kw
  OR ertugliflozin:ti,ab,kw
  OR ipragliflozin:ti,ab,kw
  OR luseogliflozin:ti,ab,kw
  OR tofogliflozin:ti,ab,kw
  OR remogliflozin:ti,ab,kw
  OR bexagliflozin:ti,ab,kw
  OR 'glucagon like peptide 1 receptor agonist'/exp
  OR 'glp-1 receptor agonist':ti,ab,kw
  OR 'glp-1 receptor agonists':ti,ab,kw
  OR 'glucagon-like peptide-1 receptor agonist':ti,ab,kw
  OR 'glucagon-like peptide-1 receptor agonists':ti,ab,kw
  OR exenatide:ti,ab,kw
  OR liraglutide:ti,ab,kw
  OR lixisenatide:ti,ab,kw
  OR dulaglutide:ti,ab,kw
  OR semaglutide:ti,ab,kw
  OR albiglutide:ti,ab,kw
  OR beinaglutide:ti,ab,kw
  OR efpeglenatide:ti,ab,kw
  OR taspoglutide:ti,ab,kw
)
NOT [08-11-2025]/sd
```

# 5. ClinicalTrials.gov

**Records retrieved:** n = 76

**Date restriction:** Study first-posted date on or before 7 November 2025, as specified in the executed query

( "Type 2 Diabetes" OR "Type 2 Diabetes Mellitus" OR T2DM ) AND ( "intensive insulin therapy" OR "intensive insulin treatment" OR "continuous subcutaneous insulin infusion" OR CSII OR "insulin pump" OR "multiple daily injections" OR MDI OR "basal-bolus insulin" ) AND ( metformin OR sitagliptin OR saxagliptin OR vildagliptin OR linagliptin OR alogliptin OR teneligliptin OR gemigliptin OR anagliptin OR evogliptin OR trelagliptin OR omarigliptin OR dapagliflozin OR empagliflozin OR canagliflozin OR ertugliflozin OR ipragliflozin OR luseogliflozin OR tofogliflozin OR remogliflozin OR bexagliflozin OR exenatide OR liraglutide OR lixisenatide OR dulaglutide OR semaglutide OR albiglutide OR beinaglutide OR efpeglenatide OR taspoglutide ) AND AREA[StudyFirstPostDate]RANGE[MIN, 11/07/2025]

# 6. China National Knowledge Infrastructure (CNKI)

**Search field:** Subject (SU)

**Date coverage:** Database inception to 7 November 2025

**Search settings:** Automatic Chinese-English expansion and synonym expansion were disabled. No methodological RCT filter was applied at retrieval; randomized allocation was verified during screening.

**Date handling:** Because the CNKI professional-search interface did not permit an exact day-level publication-date limit, records were first restricted to publication year ≤2025; records published after 7 November 2025 were then excluded manually based on the publication date shown in the database record.

**Records retrieved:** n = 583 across four drug-class searches; n = 508 unique records after within-CNKI deduplication

## 6.1 GLP-1 receptor agonists

**Records retrieved:** n = 168

(SU%=2型糖尿病 OR SU%=Ⅱ型糖尿病 OR SU%=II型糖尿病 OR SU%=T2DM)
AND
(
SU%=强化胰岛素治疗
OR SU%=胰岛素强化治疗
OR SU%=短期强化胰岛素治疗
OR SU%=短期胰岛素强化治疗
OR SU%=胰岛素泵
OR SU%=持续皮下胰岛素输注
OR SU%=CSII
OR SU%=多次胰岛素注射
OR SU%=MDI
OR SU%=基础餐时胰岛素
)
AND
(
SU%=GLP-1受体激动剂
OR SU%=胰高血糖素样肽-1受体激动剂
OR SU%=艾塞那肽
OR SU%=利拉鲁肽
OR SU%=利司那肽
OR SU%=度拉糖肽
OR SU%=司美格鲁肽
OR SU%=阿必鲁肽
OR SU%=贝那鲁肽
OR SU%=exenatide
OR SU%=liraglutide
OR SU%=lixisenatide
OR SU%=dulaglutide
OR SU%=semaglutide
OR SU%=beinaglutide
)

## 6.2 Metformin

**Records retrieved:** n = 231

```
  (SU%=2型糖尿病 OR SU%=Ⅱ型糖尿病 OR SU%=II型糖尿病 OR SU%=T2DM)
AND
(
SU%=强化胰岛素治疗
OR SU%=胰岛素强化治疗
OR SU%=短期强化胰岛素治疗
OR SU%=短期胰岛素强化治疗
OR SU%=胰岛素泵
OR SU%=持续皮下胰岛素输注
OR SU%=CSII
OR SU%=多次胰岛素注射
OR SU%=MDI
OR SU%=基础餐时胰岛素
)
AND
(
SU%=二甲双胍
OR SU%=盐酸二甲双胍
OR SU%=metformin
)
```

## 6.3 DPP-4 inhibitors

**Records retrieved:** n = 102

(SU%=2型糖尿病 OR SU%=Ⅱ型糖尿病 OR SU%=II型糖尿病 OR SU%=T2DM)
AND
(
SU%=强化胰岛素治疗
OR SU%=胰岛素强化治疗
OR SU%=短期强化胰岛素治疗
OR SU%=短期胰岛素强化治疗
OR SU%=胰岛素泵
OR SU%=持续皮下胰岛素输注
OR SU%=CSII
OR SU%=多次胰岛素注射
OR SU%=MDI
OR SU%=基础餐时胰岛素
)
AND
(
SU%=DPP-4抑制剂
OR SU%=DPP4抑制剂
OR SU%=二肽基肽酶-4抑制剂
OR SU%=西格列汀
OR SU%=沙格列汀
OR SU%=维格列汀
OR SU%=利格列汀
OR SU%=阿格列汀
OR SU%=替格列汀
OR SU%=吉格列汀
OR SU%=sitagliptin
OR SU%=saxagliptin
OR SU%=vildagliptin
OR SU%=linagliptin
OR SU%=alogliptin
)

## 6.4 SGLT2 inhibitors

**Records retrieved:** n = 82

(SU%=2型糖尿病 OR SU%=Ⅱ型糖尿病 OR SU%=II型糖尿病 OR SU%=T2DM)
AND
(
SU%=强化胰岛素治疗
OR SU%=胰岛素强化治疗
OR SU%=短期强化胰岛素治疗
OR SU%=短期胰岛素强化治疗
OR SU%=胰岛素泵
OR SU%=持续皮下胰岛素输注
OR SU%=CSII
OR SU%=多次胰岛素注射
OR SU%=MDI
OR SU%=基础餐时胰岛素
)
AND
(
SU%=SGLT2抑制剂
OR SU%=SGLT-2抑制剂
OR SU%=钠-葡萄糖协同转运蛋白2抑制剂
OR SU%=达格列净
OR SU%=恩格列净
OR SU%=卡格列净
OR SU%=艾托格列净
OR SU%=伊格列净
OR SU%=鲁格列净
OR SU%=托格列净
OR SU%=dapagliflozin
OR SU%=empagliflozin
OR SU%=canagliflozin
OR SU%=ertugliflozin
OR SU%=ipragliflozin
)

# 7. Wanfang Data

**Records retrieved:** n = 1,813

(
2型糖尿病
OR Ⅱ型糖尿病
OR II型糖尿病
OR T2DM
)
AND
(
强化胰岛素治疗
OR 胰岛素强化治疗
OR 短期强化胰岛素治疗
OR 胰岛素泵
OR 持续皮下胰岛素输注
OR CSII
OR 多次胰岛素注射
OR MDI
OR 基础餐时胰岛素
OR 基础-餐时胰岛素
)
AND
(
二甲双胍
OR 盐酸二甲双胍
OR DPP-4抑制剂
OR 二肽基肽酶-4抑制剂
OR 西格列汀
OR 沙格列汀
OR 维格列汀
OR 利格列汀
OR 阿格列汀
OR 替格列汀
OR 吉格列汀
OR SGLT2抑制剂
OR 钠-葡萄糖协同转运蛋白2抑制剂
OR 达格列净
OR 恩格列净
OR 卡格列净
OR 艾托格列净
OR 伊格列净
OR 鲁格列净
OR 托格列净
OR GLP-1受体激动剂
OR 胰高血糖素样肽-1受体激动剂
OR 艾塞那肽
OR 利拉鲁肽
OR 利司那肽
OR 度拉糖肽
OR 司美格鲁肽
OR 阿必鲁肽
OR 贝那鲁肽
)

# 8. VIP Database (China Science and Technology Journal Database)

**Publication-year limit:** Database inception to 2025

**Prespecified evidence cutoff:** 7 November 2025

**Date handling:** Records published after 7 November 2025 were manually excluded during screening

**Records retrieved:** n = 705

**Exact search strategy**

**Search line 1 — Title or Keyword**

2型糖尿病 OR T2DM

**AND**

**Search line 2 — Abstract**

强化胰岛素治疗
OR 胰岛素强化治疗
OR 短期强化胰岛素治疗
OR 胰岛素泵
OR 持续皮下胰岛素输注
OR CSII
OR 多次胰岛素注射
OR MDI
OR 基础餐时胰岛素

**AND**

**Search line 3 — Abstract**

二甲双胍
OR metformin
OR DPP-4抑制剂
OR 西格列汀
OR 沙格列汀
OR 维格列汀
OR 利格列汀
OR 阿格列汀
OR sitagliptin
OR saxagliptin
OR vildagliptin
OR linagliptin
OR alogliptin
OR SGLT2抑制剂
OR 达格列净
OR 恩格列净
OR 卡格列净
OR 艾托格列净
OR 伊格列净
OR dapagliflozin
OR empagliflozin
OR canagliflozin
OR ertugliflozin
OR ipragliflozin
OR GLP-1受体激动剂
OR 艾塞那肽
OR 利拉鲁肽
OR 利司那肽
OR 度拉糖肽
OR 司美格鲁肽
OR 贝那鲁肽
OR exenatide
OR liraglutide
OR lixisenatide
OR dulaglutide
OR semaglutide
OR beinaglutide

# 9. Chinese Medical Association database

**Records retrieved:** n = 198

(((2型糖尿病 OR T2DM) AND (强化胰岛素治疗 OR 胰岛素强化治疗 OR 短期强化胰岛素治疗 OR 胰岛素泵 OR 持续皮下胰岛素输注 OR CSII OR 多次胰岛素注射 OR MDI OR 基础餐时胰岛素)) AND (二甲双胍 OR 盐酸二甲双胍 OR DPP-4抑制剂 OR 西格列汀 OR 沙格列汀 OR 维格列汀 OR 利格列汀 OR 阿格列汀 OR SGLT2抑制剂 OR 达格列净 OR 恩格列净 OR 卡格列净 OR 艾托格列净 OR 伊格列净 OR GLP-1受体激动剂 OR 艾塞那肽 OR 利拉鲁肽 OR 利司那肽 OR 度拉糖肽 OR 司美格鲁肽 OR 贝那鲁肽)) AND 出版日期=[* TO 2025-11-07]
