# Supplementary material for: Effects of intensive insulin therapy combined with non-insulin antihyperglycemic agents on glycemic control, variability, and metabolic outcomes in patients with type 2 diabetes mellitus: a systematic review and meta-analysis
Source: Front Endocrinol (Lausanne). 2026 Oct 5;17:1948361. doi: 10.3389/fendo.2026.1948361 (PMC13677461; doi:10.3389/fendo.2026.1948361)
Supplement: Supplementary file 6 [file Table1.docx]

Supplementary Table 1

**Characteristics of included studies and participants**

All paired data are intervention / comparator. Continuous data are mean ± SD unless otherwise stated; age and diabetes duration are in years unless another unit is specified, HbA1c is in %, and BMI is in kg/m². The HbA1c eligibility criterion is the numerical study-entry threshold and is distinct from randomized-group baseline HbA1c. NR, not reported in the reviewed source; NC, not confirmed from the available source (not imputed).

| **Study / country** | **Design / population** | **Allocated n** **I / C** | **Age** **I / C** | **Diabetes duration / relevant treatment history** **I / C** | **HbA1c eligibility criterion** | **Baseline HbA1c** **I / C** | **BMI** **I / C** | **Intervention vs comparator** | **Treatment / follow-up** |
| --- | --- | --- | --- | --- | --- | --- | --- | --- | --- |
| **DPP-4i** | | | | | | | | | |
| Yuan_2014 China | Parallel-group; reported randomized P1 | 40 / 40 | 49.37 ± 11.82 / 53.13 ± 12.24 | Newly diagnosed; untreated | NR | 10.35 ± 1.76 / 10.81 ± 1.91 | 24.56 ± 3.01 / 24.43 ± 4.78 | Sitagliptin 100 mg/day + CSII vs CSII (groups B/A) | 2 weeks |
| Yuan_2015 China | Parallel-group; reported randomized P1 | 109 / 108 Baseline: 108 / 104 | 48.56 ± 13.09 / 49.27 ± 13.04 | Author-reported newly diagnosed; prior treatment NC | NR | 10.54 ± 1.41 / 10.67 ± 1.90 | 24.78 ± 2.93 / 24.21 ± 2.72 | Sitagliptin + lispro CSII vs CSII | 2 weeks; reassessment 48 h after stopping |
| Li_Jiang_2016 China | Single-centre, open-label RCT P1 | 31 / 38 | 48.65 ± 10.48 / 47.11 ± 9.85 | Author-reported newly diagnosed; prior treatment NC | NR | 9.52 ± 1.88 / 9.42 ± 1.16 | 25.36 ± 2.38 / 24.93 ± 2.23 | Saxagliptin 5 mg/day + aspart CSII vs CSII | 4 weeks |
| Wan_2016 China | Parallel-group; reported randomized P1 | 30 / 30 | 46.40 ± 5.14 / 45.18 ± 7.10 | Author-reported newly diagnosed; prior treatment NC | NR | 10.47 ± 1.33 / 10.06 ± 1.82 | 23.76 ± 3.01 / 23.56 ± 1.48 | Sitagliptin 100 mg/day + aspart CSII vs CSII | 2 weeks |
| Wang _Chen_2019 China | Parallel-group; reported randomized P2 | 52 / 49 | 56.3 ± 4.3 / NC | Duration NR; uncontrolled T2DM | NR | 9.3 ± 2.8 / 9.0 ± 2.7 | 23.5 ± 2.2 / 24.1 ± 2.5 | Saxagliptin + aspart CSII vs CSII | 2 weeks |
| Wang_Lv_2019 China | Three-arm controlled study P2 | 37 / 34 | 55.43 ± 10.93 / 52.33 ± 10.11 | 6.14 ± 5.77 / 6.58 ± 5.83 years; prior twice-daily premixed insulin; switched to aspart CSII | HbA1c 7.0%-10.0%, or FPG 7.5-16.0 mmol/L | 8.03 ± 1.84 / 8.45 ± 2.03 | 24.76 ± 6.77 / 23.67 ± 7.12 | Sitagliptin 100 mg/day + insulin-aspart CSII vs insulin-aspart CSII (groups C/A) | 2 weeks |
| Lyu_2020 China | Single-centre, open-label RCT P2 | 100 / 100 Baseline: 92 / 99 | 49.46 ± 11.64 / 50.94 ± 11.58 | 4 ± 0.17 / 3 ± 0.83 years; hospitalized uncontrolled T2DM | HbA1c ≥8.5% | 11.03 ± 1.87 / 10.94 ± 2.07 | 24.34 ± 3.19 / 24.80 ± 3.14 | Vildagliptin + lispro CSII vs CSII | 7 days |
| **GLP-1RA** | | | | | | | | | |
| Lin_2015 Taiwan, China | Parallel-group; reported randomized P2 | 30 / 21 | 52.8 ± 2.4 / 53.5 ± 3.3 (SEM) | 11.6 ± 1.3 / 12.8 ± 1.9 years (SEM); twice-daily premixed insulin before entry; switched to CSII during 3-day run-in | HbA1c 8.0%-12.0% | 10.5 ± 0.2 / 10.6 ± 0.3 (SEM) | 28.9 ± 0.8 / 27.0 ± 1.1 (SEM) | Exenatide 5 µg twice daily + aspart CSII vs placebo + CSII | 3-day CSII run-in + 3-day add-on phase |
| Lind_2015 Sweden | Multicentre, double-blind RCT P3 | 64 / 60 | 63.7 ± 8.2 / 63.5 ± 7.7 | 17.3 ± 7.6 / 17.0 ± 8.1 years; maintenance MDI ≥6 months | HbA1c 7.5%-11.5%; lower limit originally 8.0%, later amended to 7.5% | 9.0 ± 1.0 / 9.0 ± 1.1 | 33.7 ± 4.3 / 33.5 ± 4.0 | Liraglutide + MDI vs placebo + MDI | 24 weeks |
| Ke_2016 China | Parallel-group; reported randomized P1 | 20 / 19 | 42.3 ± 9.9 / 42.1 ± 7.6 | Author-reported newly diagnosed; prior treatment NC | NR | 10.0 ± 2.1 / 10.2 ± 2.0 | 25.4 ± 2.8 / 25.5 ± 2.4 | Liraglutide + CSII vs CSII | CSII: target + 14 days; follow-up 12 weeks |
| Li_2017  China | Single-centre, open-label RCT P2 | 18 / 18 | 47.56 ± 13.15 / 49.92 ± 9.46 | ≥0.5 years (eligibility); established uncontrolled T2DM | HbA1c 7.5%-12.0% | 9.67 ± 1.34 / 8.84 ± 1.38 | 26.59 ± 3.33 / 26.25 ± 4.72 | Exenatide 5→10 µg twice daily + aspart CSII vs CSII | 5 weeks |
| Li_2019  China | Parallel-group; reported randomized P1 | 30 / 30 | 50.00 (42.50-66.00) / 46.00 (42.50-59.00) | Author-reported newly diagnosed; prior treatment NC | NR | 10.40 (10.30-11.80) / 11.20 (9.30-12.80) | 27.30 (25.90-28.72) / 29.74 (26.33-29.98) | Liraglutide 0.6-1.8 mg/day + CSII vs CSII | 14 days |
| Joubert_2020 France | Multicentre, double-blind RCT P3 | 28 / 18 | 61 ± 7 / 58 ± 8 | 19.3 ± 7.9 / 18.4 ± 7.7 years; maintenance intensive insulin (CSII/MDI) | HbA1c 7.5%-10.0% | 8.8 ± 0.8 / 8.9 ± 0.9 | 35.5 ± 4.2 / 35.1 ± 4.4 | Exenatide 5→10 µg twice daily + insulin vs placebo + insulin (CSII/MDI) | 6 months |
| Kang_2020 China | Parallel-group; reported randomized P1 | 99 / 99 | 51.97 ± 6.78 / 52.35 ± 6.98 | Author-reported newly diagnosed; obese; prior treatment NC | NR | 8.24 ± 0.87 / 8.19 ± 0.83 | 29.51 ± 1.48 / 29.87 ± 1.69 | Liraglutide 0.6 mg/day + aspart CSII vs CSII | 2 weeks |
| Fu_2022 China | Parallel-group; reported randomized P2 | 61 / 60 | 66.42 ± 7.38 / 66.31 ± 7.45 | 5.45 ± 1.24 / 5.36 ± 1.27 years; established T2DM | NR | 9.65 ± 1.82 / 9.87 ± 1.72 | NR | Liraglutide 0.6-1.8 mg/day + prandial insulin/glargine MDI vs MDI | 3 months |
| Li_Shuang _2022 China | Parallel-group; reported randomized P2 | 40 / 40 | 60.24 ± 6.78 / 59.51 ± 6.82 | 8.05 ± 1.39 / 7.89 ± 1.41 years; established T2DM | NR | NR | 28.10 ± 3.48 / 27.75 ± 3.51 | Liraglutide 0.6-1.8 mg/day + aspart CSII vs CSII | 2 weeks |
| Ma_2022 China | Parallel-group; reported randomized P1 | 46 / 46 | 55.39 ± 6.45 / 55.72 ± 6.54 | 6.69 ± 1.07 / 6.37 ± 1.11 months; author-reported newly diagnosed; obese | NR | NR | 28.49 ± 1.26 / 28.53 ± 1.38 | Liraglutide 0.6→1.2 mg/day + aspart/detemir MDI vs MDI | 1 month |
| Luo_2025 China | Single-centre, open-label RCT P1 | 15 / 15 | 37.8 ± 9.5 / 43.1 ± 8.2 | Author-reported newly diagnosed; prior treatment NC | NR | 9.9 ± 1.7 / 9.9 ± 1.8 | 27.5 ± 5.2 / 28.2 ± 1.6 | Continuous beinaglutide, titrated to 0.6 mg/day, + aspart CSII vs CSII | 1-week lead-in; 4 randomized weeks |
| **Metformin** | | | | | | | | | |
| Xu_2009 China | Parallel-group; reported randomized P2 | 24 / 18 | 54 ± 0.2 / 52 ± 1 | ≤3 years; short-duration uncontrolled T2DM | NR | NR | NR | Metformin 500 mg three times daily + aspart/NPH MDI vs MDI | 2 weeks |
| Liang_2012 China | Parallel-group; reported randomized P1 | 25 / 25 | 46 ± 10 / 44 ± 12 | 1.3 ± 0.7 / 1.2 ± 0.9 years; called newly diagnosed; untreated | NR | NR | 27.1 ± 1.4 / 25.9 ± 2.1 | Metformin ER 1,000 mg/day + aspart CSII vs CSII | 15 days; OGTT day 16 |
| Huang_2013 China | Four-arm RCT; prespecified eligible pairwise contrast P1 | 40 / 40 | 49.2 ± 11.3 / 50.8 ± 9.7 | Newly diagnosed; untreated. Other randomized arms: rosiglitazone and α-lipoic acid (outside review intervention criteria) | NR | 11.1 ± 1.8 / 10.9 ± 1.8 | 25.8 ± 3.1 / 25.0 ± 3.1 | Metformin + rapid-acting insulin CSII vs CSII (eligible selected contrast) | CSII: target + 2 weeks; metformin: 3 months |
| Tong_2014 China | Parallel-group; reported randomized P1 | 40 / 40 | 53.6 ± 11.8 / 51.8 ± 12.0 | Newly diagnosed; untreated | NR | 10.7 ± 1.8 / 10.8 ± 1.8 | 26.0 ± 2.8 / 25.8 ± 3.6 | Metformin + aspart CSII vs CSII | Target + 2 weeks |
| Nuerkamali_2017 China | Parallel-group; reported randomized P1 | 56 / 56 | 54.72 ± 4.29 / 54.69 ± 4.25 | 15.62 ± 2.23 / 15.59 ± 2.15 years; previously untreated/treatment-naïve. Original source used 'newly diagnosed' | NR | NR | NR | Metformin ER 1,000 mg/day + CSII vs CSII | 15 days |
| Hu_2018 China | Parallel-group; reported randomized P2 | 40 / 40 | 48.02 ± 6.31 / NC | 14.72 ± 2.11 / 15.03 ± 1.52 years; established T2DM | NR | NR | 27.51 ± 2.02 / NC | Metformin ER 1,000 mg/day + CSII vs CSII | 15 days |
| Jin_2018 China | Parallel-group; reported randomized P1 | 100 / 100 | 58.84 ± 4.68 / 58.89 ± 4.62 | 1.35 ± 0.65 / 1.34 ± 0.62 years; called newly diagnosed; treatment history NC | NR | NR | NR | Metformin ER 1,000 mg/day + CSII vs CSII | 15 days |
| Li_2018 China | Pooled secondary report of two open-label RCTs P1 | Pooled sample: 95 / 93 | 52.32 ± 8.24 / 50.17 ± 11.57 | Author-reported newly diagnosed; underlying RCTs: CliCTR-TRC-10001218 and NCT03226210 | NR | 9.67 ± 1.34 / 8.84 ± 1.38 | 24.42 ± 3.27 / 25.15 ± 3.16 | Metformin ≥1,000 mg/day + aspart CSII vs CSII | Approximately 2 weeks |
| Ye_2018 China | Parallel-group; reported randomized P2 | 35 / 35 | 56.2 ± 15.3 / 55.2 ± 11.8 | 9.53 ± 8.43 / 11.5 ± 8.6 years; established T2DM | NR | NR | 27.14 ± 1.83 / 27.22 ± 1.74 | Metformin ER 500 mg/day + CSII vs CSII | 2 weeks |
| Cao_2019 China | Parallel-group; reported randomized P2 | 50 / 50 | 46.41 ± 3.85 / 46.39 ± 3.87 | 13.05 ± 3.19 / 13.08 ± 3.21 years; established T2DM | NR | NR | NR | Metformin ER 1,000 mg twice daily + aspart CSII vs CSII | 3 weeks |
| Hu_2021 China | Parallel-group; reported randomized P1 | NC Baseline: 35 / 35 | NC | Newly diagnosed; drug-naïve men | NR | NC | NC | Metformin 1,500 mg/day + continued insulin pump vs pump alone | 3-5-day run-in; 30 ± 2 days after allocation |
| Lei_2023 China | Parallel-group; reported randomized P2 | 59 / 59 | 60.23 ± 10.24 / 60.48 ± 10.35 | 5.13 ± 1.46 / 5.27 ± 1.53 years; established T2DM | NR | NR | NR | Metformin 250 mg three times daily + CSII vs CSII; insulin formulation unclear | 1 month |
| **SGLT2i** | | | | | | | | | |
| Okajima_2017 Japan | Four-arm RCT; selected contrast P2 | 15 / 15 | 57 ± 8 / 55 ± 14 | 9 ± 11 / 6 ± 6 years; established uncontrolled T2DM; no prior insulin | HbA1c ≥10.0% | 12.4 ± 2.5 / 12.3 ± 1.9 | 26.0 ± 4.8 / 24.5 ± 3.2 | Ipragliflozin + glulisine/glargine MDI vs MDI (selected arms) | Approximately 14 inpatient days |
| Du_2019 China | Parallel-group; reported randomized P2 | 65 / 65 | 56.7 ± 7.1 / 54.6 ± 6.3 | Duration NR; prior insulin ≥30 IU/day with FBG >9.0 mmol/L; prior regimen not established as eligible IIT | HbA1c 9.0%-16.0%; prior insulin ≥30 IU/day and FBG >9.0 mmol/L | 11.5 ± 2.3 / 11.4 ± 3.2 | 26.5 ± 2.1 / 26.8 ± 2.3 | Dapagliflozin 10 mg/day + lispro/detemir basal-bolus MDI vs placebo + MDI | 12 weeks |
| Peng_2019 China | Parallel-group; reported randomized P2 | 40 / 40 | 54.5 ± 10.3 / 54.7 ± 8.3 | 6.9 ± 2.3 / 7.0 ± 1.9 years; overweight/obese uncontrolled T2DM | HbA1c 9.0%-16.0% | 11.9 ± 2.4 / 11.4 ± 2.3 | 25.7 ± 2.7 / 25.8 ± 2.6 | Empagliflozin 10 mg/day + glulisine/detemir MDI vs placebo + MDI | 12 weeks |
| Xin_2019 China | Parallel-group; reported randomized P2 | 40 / 40 | 55.65 ± 7.17 / 57.40 ± 8.30 | 7.75 ± 2.03 / 7.05 ± 2.42 years; established uncontrolled T2DM | HbA1c ≥9.0% | 12.21 ± 1.45 / 11.93 ± 1.63 | 28.42 ± 2.08 / 27.76 ± 1.77 | Dapagliflozin 10 mg/day + regular/NPH MDI vs MDI | 12 weeks |
| Hao_2021 China | Parallel-group; reported randomized Mixed P1/P2 | 33 / 33 | 44.41 ± 12.96 / 47.38 ± 13.74 | 48.86 ± 56.06 / 56.83 ± 53.77 months; newly diagnosed 8/30 vs 6/30 | P1: HbA1c >9.0% or FPG >11.1 mmol/L; P2: HbA1c >7.0% with stable oral therapy ≥2 months | 10.11 ± 1.36 / 10.41 ± 1.65 | 26.66 ± 2.89 / 27.35 ± 3.36 | Empagliflozin 10 mg/day + aspart/degludec MDI vs MDI | 14 days |
| Jiang_2021 China | Single-centre, open-label RCT P1 | 34 / 32 | 50.0 ± 10.4 / 46.3 ± 10.1 | Author-reported newly diagnosed; prior treatment NC | NR | 10.2 ± 1.77 / 10.5 ± 1.6 | 25.6 ± 3.0 / 24.1 ± 3.4 | Dapagliflozin + CSII vs CSII | 5 weeks |
| Lin_2022 China | Parallel-group; reported randomized P1 | 30 / 30 | 41.9 ± 9.1 / 39.3 ± 7.9 | Author-reported newly diagnosed; prior treatment NC | NR | 11.6 ± 2.0 / 11.9 ± 2.2 | 24.83 ± 2.61 / 24.19 ± 3.47 | Dapagliflozin + basal-bolus insulin vs insulin alone | Target + 2 weeks; 12-week off-drug follow-up |
| Fushimi_2025 Japan | Single-centre, open-label RCT Mixed P1/P2 | 18 / 17 | 63.8 ± 15.2 / 65.6 ± 16.8 | 7.9 ± 9.5 / 10.8 ± 13.5 years; eligibility allowed diet/untreated, oral agents, or once-daily insulin | HbA1c ≥10.0% if diet-treated/untreated; ≥8.0% if receiving oral agents or once-daily insulin | 11.2 ± 1.8 / 11.1 ± 2.1 | 24.5 ± 4.2 / 27.4 ± 5.8 | Empagliflozin + lispro/glargine U300 MDI vs MDI | 7 days |
| Wang _2025 China | Parallel-group; reported randomized P1 | 30 / 30 | 52.52 ± 8.24 / 51.83 ± 7.81 | Author-reported newly diagnosed; prior treatment NC | NR | 9.20 ± 1.12 / 9.22 ± 1.24 | 25.14 ± 2.98 / 25.32 ± 3.14 | Dapagliflozin 10 mg/day + aspart CSII vs CSII | 12 weeks |

**Interpretation notes**

Population categories: P1, treatment-naïve or previously untreated patients with T2DM who initiated eligible IIT as part of the study protocol; P2, patients with established T2DM and inadequate glycemic control who initiated eligible IIT (CSII or basal-bolus MDI) as part of the study protocol, including initiation during a short prerandomization run-in or stabilization period; P3, patients with established T2DM who were already receiving stable maintenance IIT (CSII or basal-bolus MDI) before study enrollment but remained inadequately controlled at randomization. P1 classification is based on prior treatment status rather than diabetes duration; patients with established disease duration may therefore be P1 if they were previously untreated. Mixed populations are labeled accordingly.

'Newly diagnosed' is retained only as an author-reported descriptor and is not used as a surrogate for numerical diabetes duration. Where numerical duration conflicts with this descriptor, reported duration and documented treatment history take precedence. HbA1c eligibility criteria reproduce numerical thresholds explicitly reported for the original trials; NR does not imply that no glycemic entry criterion was applied.

Fushimi 2025 is classified as Mixed P1/P2 at the eligibility level because the entry criteria permitted diet-treated/untreated participants and participants receiving oral agents or once-daily insulin; the randomized sample's treatment-status distribution was not separately confirmed. Hao 2021 is Mixed P1/P2 because both newly diagnosed and previously treated patients were represented.

Allocated n is distinct from the sample represented by the reported baseline table. When different, both are displayed. A reported/completer n is not substituted for an unconfirmed allocation n. Baseline data for Hao 2021, Lyu 2020, Okajima 2017, and Yuan 2015 represent the reported analyzed/completer sample, not all allocated participants. Lind 2015 uses all randomized participants.

For multi-arm trials, only prespecified pairwise comparisons meeting the review eligibility criteria were included in quantitative synthesis, without double-counting participants. Huang 2013 retained metformin + CSII versus CSII alone; its rosiglitazone and α-lipoic acid arms were outside the review intervention criteria.

Previous use of premixed insulin before randomization was not an exclusion criterion when the randomized intervention used eligible IIT. Lin 2015 is P2 because participants used twice-daily premixed insulin before study entry and switched to CSII during the trial run-in. Du 2019 is P2 because prior insulin use (≥30 IU/day) did not establish prior eligible maintenance IIT; the randomized treatment introduced lispro/detemir basal-bolus MDI.

Li 2018 is one pooled secondary publication derived from two previously conducted open-label RCTs (CliCTR-TRC-10001218 and NCT03226210). Neither underlying RCT was otherwise represented by another included report. Because trial-specific outcome data were not separately reported, Li 2018 contributes one pooled analytical dataset to each relevant meta-analysis while representing two underlying randomized trials.

Lin 2015 reports mean ± SEM throughout. Li 2019 (liraglutide) reports median (Q1-Q3) for age, HbA1c, and BMI. CSII, continuous subcutaneous insulin infusion; DPP-4i, dipeptidyl peptidase-4 inhibitor; ER, extended release; FBG/FPG, fasting blood/plasma glucose; GLP-1RA, glucagon-like peptide-1 receptor agonist; IIT, intensive insulin therapy; MDI, multiple daily injections; NPH, neutral protamine Hagedorn insulin; OGTT, oral glucose tolerance test; SGLT2i, sodium-glucose cotransporter-2 inhibitor; T2DM, type 2 diabetes mellitus.
