# Supplementary material for: Effects of intensive insulin therapy combined with non-insulin antihyperglycemic agents on glycemic control, variability, and metabolic outcomes in patients with type 2 diabetes mellitus: a systematic review and meta-analysis
Source: Front Endocrinol (Lausanne). 2026 Oct 5;17:1948361. doi: 10.3389/fendo.2026.1948361 (PMC13677461; doi:10.3389/fendo.2026.1948361)
Supplement: Supplementary file 7 [file Table3.docx]

Supplementary Table 3. GRADE Summary of Findings

**Population:** Adults with type 2 diabetes receiving intensive insulin therapy (IIT), according to the included randomized trials. **Intervention:** IIT plus a non-insulin antihyperglycaemic agent within the specified drug class. **Comparator:** IIT alone or matching placebo plus IIT.

| **Outcome / drug class** | **No. of studies** | **Effect estimate (95% CI)** | **Certainty of evidence** | **Plain-language interpretation** | **Main reasons for downgrading** |
| --- | --- | --- | --- | --- | --- |
| **Efficacy and glycaemic-variability outcomes** | | | | | |
| **DPP-4i – MAGE** | 4 | MD −1.39 mmol/L (95% CI −1.77 to −1.01) | **Moderate** | DPP-4i added to IIT likely reduces MAGE. | Risk of bias (−1). |
| **GLP-1RA – MAGE** | 3 | MD −0.61 mmol/L (95% CI −1.19 to −0.02) | **Very low** | The evidence is very uncertain about the effect of GLP-1RA on MAGE. | Risk of bias (−1), inconsistency (−1), imprecision (−1). |
| **DPP-4i – SDBG** | 2 | MD −0.46 mmol/L (95% CI −0.75 to −0.17) | **Very low** | The evidence is very uncertain about the effect of DPP-4i on SDBG. | Risk of bias (−1), inconsistency (−1), imprecision (−1). |
| **SGLT2i – SDBG** | 3 | MD −0.28 mmol/L (95% CI −0.44 to −0.11) | **Moderate** | SGLT2i added to IIT likely reduces SDBG. | Risk of bias (−1). |
| **DPP-4i – LAGE** | 3 | MD −1.20 mmol/L (95% CI −1.66 to −0.75) | **Moderate** | DPP-4i added to IIT likely reduces LAGE. | Risk of bias (−1). |
| **SGLT2i – TIR** | 3 | MD +8.23 percentage points (95% CI 4.13 to 12.32) | **Moderate** | SGLT2i added to IIT likely increases TIR. | Risk of bias (−1). |
| **GLP-1RA, longer duration – Body weight** | 3 | MD −3.63 kg (95% CI −4.94 to −2.32) | Low | GLP-1RA may reduce body weight in the post hoc longer-duration subgroup, but this subgroup-specific finding should be interpreted cautiously. | Risk of bias (−1); indirectness/credibility due to post hoc duration subgrouping (−1). |
| **DPP-4i – Total insulin dose** | 2 | MD −0.05 (95% CI −0.10 to −0.01; pooled insulin-dose scale) | **Low** | DPP-4i may slightly reduce total insulin dose. | Risk of bias (−1), imprecision (−1). |
| **GLP-1RA – Total insulin dose** | 5 | MD −0.10 (95% CI −0.19 to −0.02; pooled insulin-dose scale) | **Low** | GLP-1RA may reduce total insulin dose. | Risk of bias (−1), inconsistency (−1). |
| **Safety outcomes** | | | | | |
| **DPP-4i – Hypoglycemia** | 4 | RR 0.53 (95% CI 0.31 to 0.91) | **Low** | DPP-4i may reduce the risk of hypoglycemia. | Risk of bias (−1), imprecision (−1). |
| **SGLT2i – Hypoglycemia** | 5 | RR 0.30 (95% CI 0.09 to 0.94) | **Very low** | The evidence is very uncertain about whether SGLT2i reduces hypoglycemia risk; this should not be interpreted as an established class benefit. | Risk of bias (−1), very serious imprecision (−2). |
| **GLP-1RA – GI-AEs** | 7 | RR 5.28 (95% CI 1.81 to 15.40) | **Low** | GLP-1RA may increase GI-AEs, but the magnitude of the increase is uncertain. | Risk of bias (−1), imprecision (−1). |

**Abbreviations:** CI, confidence interval; DPP-4i, dipeptidyl peptidase-4 inhibitor; GI-AEs, gastrointestinal adverse events; GLP-1RA, glucagon-like peptide-1 receptor agonist; IIT, intensive insulin therapy; LAGE, largest amplitude of glycaemic excursions; MAGE, mean amplitude of glycaemic excursions; MD, mean difference; RR, risk ratio; SDBG, standard deviation of blood glucose; SGLT2i, sodium-glucose cotransporter-2 inhibitor; TIR, time in range.
